# The Role of Electrospun Nanomaterials in the Future of Energy and Environment

**DOI:** 10.3390/ma14030558

**Published:** 2021-01-25

**Authors:** Mitra Baghali, W.A.D.M. Jayathilaka, Seeram Ramakrishna

**Affiliations:** Center for Nanotechnology and Sustainability, Department of Mechanical Engineering, National University of Singapore, Singapore 117576, Singapore; dr_mbaghaie20@yahoo.com (M.B.); E0147055@u.nus.edu (W.A.D.M.J.)

**Keywords:** electrospinning, nanofibrous materials, energy conversion, energy storage, environment

## Abstract

Electrospinning is one of the most successful and efficient techniques for the fabrication of one-dimensional nanofibrous materials as they have widely been utilized in multiple application fields due to their intrinsic properties like high porosity, large surface area, good connectivity, wettability, and ease of fabrication from various materials. Together with current trends on energy conservation and environment remediation, a number of researchers have focused on the applications of nanofibers and their composites in this field as they have achieved some key results along the way with multiple materials and designs. In this review, recent advances on the application of nanofibers in the areas—including energy conversion, energy storage, and environmental aspects—are summarized with an outlook on their materials and structural designs. Also, this will provide a detailed overview on the future directions of demanding energy and environment fields.

## 1. Introduction

The world’s population is growing annually, raising concerns for energy consumption and its impact on the environment. According to recent publications [1,2,3], 80% of energy consumption derives from fossil fuels like oil, coal, and natural gas. Furthermore, the use of these finite resources leads to severe environmental problems such as air pollution, global warming, and water pollution [4,5]. As the depletion of fossil fuels and environmental pollution continues, there is an urgent need for the discovery and development of new competitive sustainable materials for energy power sources, instead of traditional fossil fuel counterparts. These ceaseless efforts have led to prominent energy conversion and storage devices such as solar cells, fuel cells, lithium-ion batteries, lithium-sulfur batteries, sodium-ion batteries, lithium-air batteries, and supercapacitors [6,7,8,9]. Besides, to overcome the environmental problems ensuing from fossil fuels consumption and to reduce water and air pollution, many novel techniques have been devised and some of them have already been used [10,11,12].

In current years, nanomaterials are quite known in being potential candidates for energy materials and for removing the pollutants due to their intrinsic properties like high porosity, large surface area, and reduced dimensions [13]. Herein, developing nano sized materials is one of the effective methods to cope with energy and environmental demands throughout the world.

Until now, one-dimensional (1D) nanomaterials—such as nanofibers (NFs), nanowires (NWs), nanotubes (NTs), nanoneedles (NNs), and nanorods (NRs)—have been synthesized and the potential of their applications in diverse fields associated with energy and environmental crisis including ion infused batteries, biosensors, high performance filtration, heavy metal adsorption have been reported. Many synthetic approaches have been employed to produce nanomaterials, where electrospinning technique has outshined as a promising route. Electrospun nanomaterials with unique properties—including high surface-to-volume ratio, low weight, high porosity, tunable morphologies, and strength—can be synthesized through a simple and cost-effective process. They can be finely tuned depending on the solution and electrospinning process parameters [14]. A wide range of synthetic and natural polymers and polymer blends, composites, and ceramics have been fabricated and a broad spectrum of micron to nanosized electrospun materials have been reported [15,16].

Electrospinning offers opportunities for designing and manufacturing new materials for improving the energy generation, conversion, and storage devices. Application of electrospun nanofibrous materials for energy and environmental issues have been studied in several reviews [17,18,19,20]. Still, with the rising demand on energy resources and environmental remediation, an updated and systematic review is a necessity to identify the future directions of the field. Hence, this review is focused on providing an overview of recent research on electrospun nanofibrous materials and the role of these materials in the future of energy and environment.

## 2. Energy Conversion

The world is facing the exponentially increasing energy demands. Apart from the unprecedented decline in global energy demand (3.8%) in 2020 owing to the impacts of the Covid-19 crises and lockdowns, world total energy demands are forecasted to increase by 1.3% each year according to the international energy agency’s annual projections (the current policies scenario) [21]. Therefore, it is imperative that the energy policies move toward renewable energy power sources, more effective energy storage and conversion devices. Electrospinning has played a role in triggering innovations in the field of electrospun nanomaterials for energy-related applications.

### 2.1. Dye-Sensitized Solar Cells

**Figure 1:**
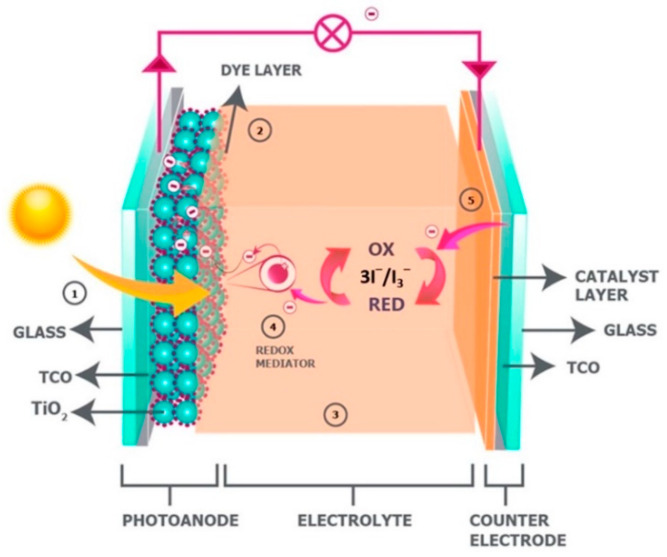
Structure of typical dye-sensitized solar cell. Reprinted from [25] with open access license.

Electrical energy conversion from renewable sources—such as wind, sun, and tide—is one of the considerable concerns in the 21st century world. Converting sunlight directly into electrical energy using photovoltaic technology have been considered as one of the low-cost and high efficiency methods. For the first time, B. O’Regan and M. Grätzel reported dye-sensitized solar cells (DSSCs) in 1991 for obtaining electricity [22]. During the last few decades, DSSC technology has been developed and power conversion efficiency of this technology has been reported as greater than 14% [23]. Typical DSSC is comprised of a photoanode, a counter electrode (CE), and an electrolyte as a separator as shown in Figure 1 [24].

In dye-sensitized solar cells, the photoanode has a vital role in the performance of photovoltaic cells as it carries dye and transports the photo-generated electrons of excited dye molecules to the collection electrode. The photoanode consists of a transparent conducting oxide covered by a layer of semiconductor, hence, the structure and morphology of the semiconductor are very important in cell fabrication [26,27,28,29]. Nanoparticulate photoanodes were commonly used because they can provide many active sites for dye adsorption, but inter-particle boundaries lead to reduce electron transport rate and increase electron trapping. In order to tackle this problem, other nanostructures such as nanofibers have been employed [30,31] and they were widely used as photoanode materials for DSSCs [32,33,34,35].

Metal oxide nanofibers, such as ZnO [36], TiO_2_ [32,35,37,38,39], and SnO_2_ [40] have been utilized as photoanode materials in solar cells. Up to now, TiO_2_ nanofibers are most successful for fabrication of DSSC due to their advantages like porosity, charge transfer, and dye absorption. Mali et al. and Song et al. used TiO_2_ nanofibers to fabricate DSSCs. They reported that employing TiO_2_ nanofibers improved the photovoltaic properties in comparison with TiO_2_ nanoparticles [41,42]. Motlak et al. produced Cd-doped TiO_2_ nanofibers which were effectively used to improve the performance of a DSSC due to an increase in the density of the electrons in the conduction band and a reduction in the optical energy gap [43]. A DSSC based on a Cd-doped TiO_2_ nanofiber had 2.945% power conversion efficiency. In DSSCs, the counter electrode (CE) is responsible for transmitting and collecting electrons. In the conventional CE, Pt is the common material that enables catalysis of the I_3_^−^ reduction and leads to overpotential reduction during the process. However, it is known as an expensive and scarce metal, while iodide (I_3_^−^/I^−^) redox couple in the electrolyte decreases its electrocatalytic activity that diminishes the stability of DSSCs [44,45]. Recently, numerous efforts have been made to explore various materials such as conducting polymers [46,47], metal nitrides [48,49], inorganic semiconductor materials [50,51,52,53], metal sulfides [54], and carbon materials [55,56,57] and use them as CE material instead of expensive Pt. Among these materials, carbon materials like carbon nanofibers (CNFs) fabricated using electrospinning have become promising candidates as the counter electrode in DSSCs. In 2010, Joshi et al., reported a typical research, in which carbon nanofibers CE caused fast I_3_^−^ reduction rate, large capacitance and low charge-transfer resistance and the efficiency was lower than Pt counter electrode [58].

Park et al. [59] used coaxial electrospinning to prepare CNFs with hollow core/highly mesoporous shell structure (Meso-HACNF) using poly(methyl methacrylate) (PMMA) as a pyrolytic core precursor with either polyacrylonitrile (PAN) or PAN/PMMA blended polymer as a carbon shell precursor to promote the electron and ion transfer. Also, energy conversion efficiency of DSSC was obtained 7.21% compared with 7.69% of Pt. Song et al. [60] developed flexible dye-sensitized solar cells with well electro-catalytic activity. For this purpose, the carbon nanotubes (CNTs) were employed to modify the flexible TiO_2_/C nanofibrous film. In addition, the conductivity, the specific surface area and the electro-catalytic activity were increased, and the conversion efficiency was about 3.4%. Cobalt-titanium carbide nanoparticles (Co-TiC NPs) embedded on carbon nanofibers were fabricated, the results showed that the obtained composite was effective, providing stable electrocatalytic activity and conductivity and improved catalytic activity in triiodide reduction [61].

### 2.2. Perovskite Solar Cells

**Figure 2:**
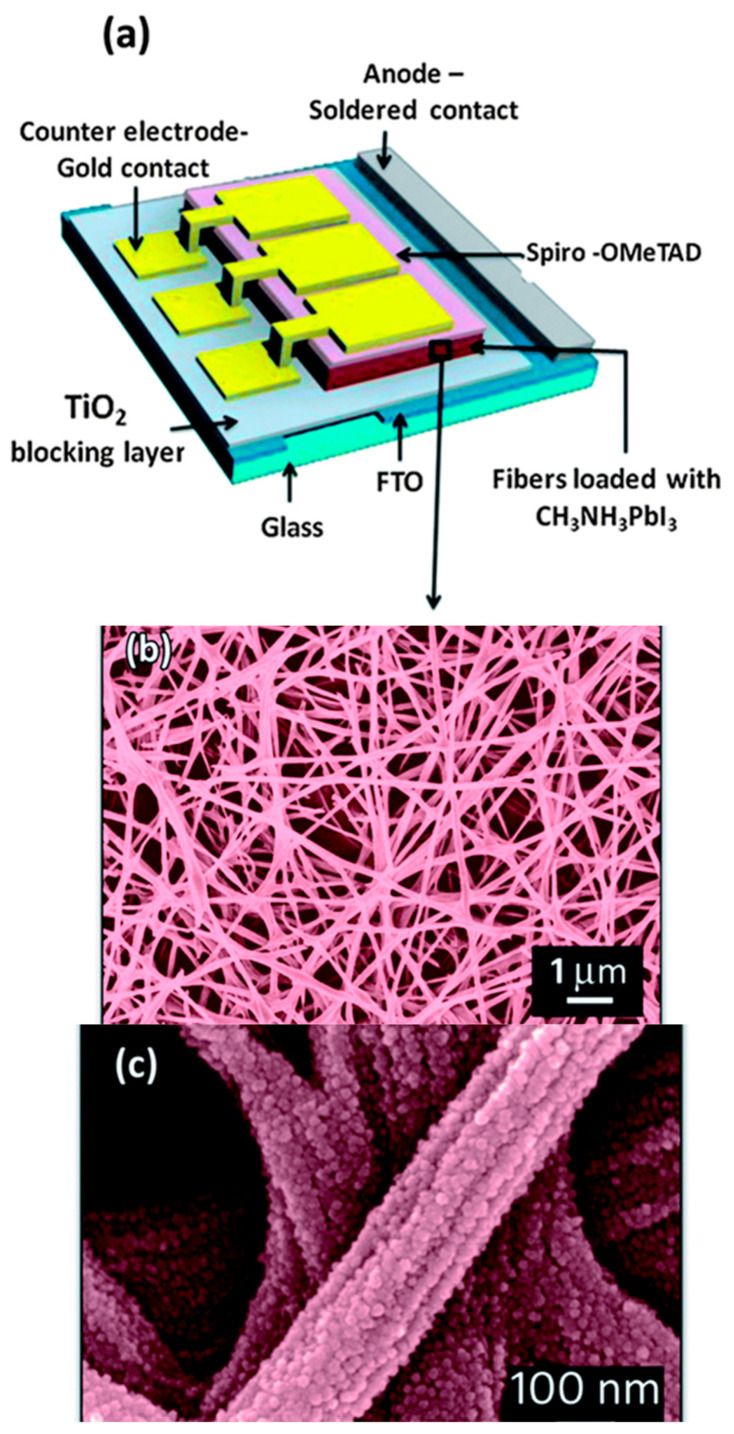
(**a**) Schematic illustration of the various components of nanofiber-based perovskite solar cells. FESEM (field emission scanning electron microscope) of (**b**) the annealed nanofiber film at 450 °C for 5 h and (**c**) of TiCl_4_ treated rough nanofibers, which were employed in the perovskite solar cells. Reproduced from [70] with permission from The Royal Society of Chemistry.

Perovskite solar cell (PSC) is one of the specific DSSC which has been paid special attention in energy devices for its good performance and high application potential and has experienced considerable improvement with the efficiency from 3.8% to 25% [62,63,64]. Perovskite has a structure of ABX_3_ (A: CH_3_NH_3_^+^, B: Pb/Sn, X: I^−^/Br^−^/Cl^−^) and some properties of the perovskite material such as broad wavelength light absorption (up to a wavelength of 800 nm) [65], adjustable band gap, high charge carrier mobility (25 cm^2^/(V s)) [66], and long-range carrier diffusion length (>1 μm) [67] mainly led this rapid growth of efficiency. In 2009, Miyasaka et al., used CH_3_NH_3_PbI_3_ (methylammonium lead iodide) for the first time in DSSCs as the sensitizer, but it had a conversion efficiency of 3.8% and CH_3_NH_3_PbI_3_ has poor stability in the liquid electrolyte [68]. In 2012, solid-state perovskite solar cells were developed which had excellent properties [69]. Dharani et al. [70] combined electrospun TiO_2_ nanofibers with CH_3_NH_3_PbI_3_ perovskite to produce a solar cell with a conversion efficiency of 9.8% which is determined by the open porosity of the electrospun nanofiber network which varies with TiO_2_ nanofiber photo-anode thicknesses and fiber diameters. Figure 2 illustrates the device architecture of the nanofiber-based perovskite cell.

**Figure 3:**
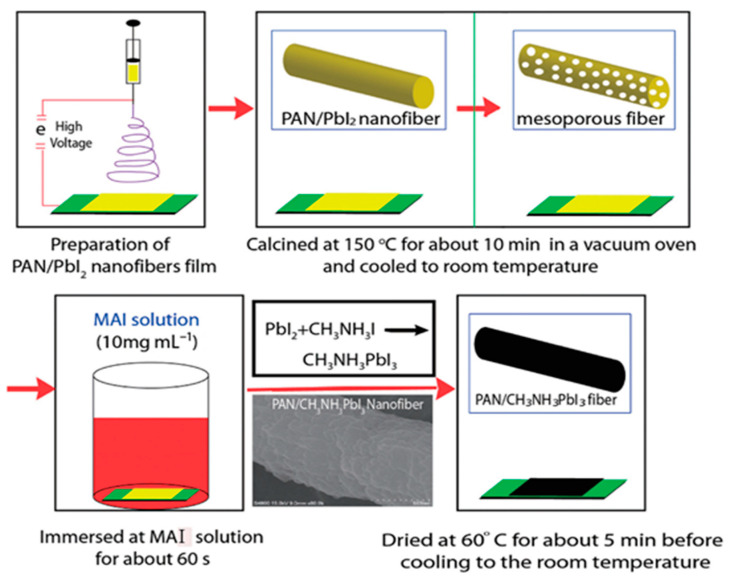
Preparation process of PAN/CH3NH3PbI3 composite nanofiber film. Reprinted from [71] with open access license.

Besides, in 2016, Lu et al. [71], prepared a polymer based CH_3_NH_3_PbI_3_ perovskite composite nanofiber film by electrospinning method. The diameter of the PAN/CH_3_NH_3_PbI_3_ nanofibers was around 500 nm and good light-harvesting capabilities were demonstrated with the UV–vis absorption spectrum. Figure 3 shows the preparation process of PAN/CH_3_NH_3_PbI_3_ composite nanofiber film.

In 2019, Hong et al., studied mixed-halide mixed-cation perovskite solar cells based on rGO-TiO_2_ composite nanofibers. They employed the electrospun reduced graphene oxide-titanium oxide composite nanofibers as an electron transporting materials to fabricate mixed-cation lead mixed-halide (FAPbI_3_)_0.85_(MAPbBr_3_)_0.15_ perovskite solar cells. As a result, the optimized rGO_4_-TiO_2_ composite NFs based electron transporting materials (ETM) exhibited 17.66% CE [72]. Ramakrishna et al. prepared flexible solar yarns with 15.7% photo conversion efficiency (PCE), based on electrospun perovskite composite nanofibers. In this study, under various conditions of electrospinning voltage and humidity, it demonstrated to be an effective technique of controlling CH_3_NH_3_PbI_3_ crystal size, quality, and distribution on poly(vinylpyrrolidone) (PVP) nanofibers. The obtained CH_3_NH_3_PbI_3_-PVP nanofibers, which were engineered under optimized conditions, showed enhanced properties, promoting excellent photovoltaic results [73].

### 2.3. Fuel Cells

Fuel cell-based technologies based on environmentally friendly and sustainable energy conversion devices are at the frontline toward reducing human reliance on fossil fuel resources. Typically, fuel cells are consisted of an electrolyte material filled between a negative anode and positive cathode employing chemical energy in fuels (often hydrogen or hydrogen-rich compounds) into electricity through an electrochemical redox reaction. The fuel stream is inserted at the anode side and dissociated into electrons and ions through a catalytic reaction. An oxidant stream (usually oxygen (air)) is fed at the cathode side. Migration of ions through the electrolyte toward the opposite charged electrode and flow of electrons in the external circuit provides electricity. Electrons, oxygen, and ions are recombined at the cathode site, as is the case of hydrogen-powered fuel cell water being produced as the basis of zero-emission hydrogen powered electric vehicles. Based on the electrolyte composition, different types of fuel cells have been developed including polymer electrolyte membrane (PEM) fuel cells (or proton exchange membrane fuel cells), direct methanol or direct ethanol fuel cells, alkaline fuel cells, phosphoric acid fuel cells, bio and microbial fuel cells, etc. During the past decades, polymer electrolyte membrane fuel cells have become the focus of attention in developing electric powered vehicles. Membrane, anode, and cathode catalyst layers and porous gas diffusion layers are main parts of membrane electrode assembly in polymer electrolyte membranes fuel cells.

Electrospun nanofibrous membranes have offered opportunities for manufacturing and designing new electrocatalyst layers (electrode) and catalyst supporting materials. Moreover, in comparison to current approaches based on carbon paper and carbon cloth materials in gas diffusion layers (GDLs), application of electrospun nanofibrous supporting materials renders innovative conductive materials for electrons to reach the electrodes more easily and provides a better gas diffused passage for gaseous reactants and products to the catalyst surfaces.

#### 2.3.1. Electrocatalysts and Supports by Electrospinning

Porous architecture of nanofibrous-supported electrodes enhances fuel cell performance by facilitating mass and charge transport in proton-exchange-membrane fuel cells. Additionally, there have been many attempts toward replacing conventional use of high-priced platinum-based nano-catalysts (Pt nanoparticles/C or Pt black) in commercial polymer electrolyte membrane fuel cells with cost-effective alternatives while maintaining fuel cell performance. For instance, low-cost platinum free nanofiber-based cathodes proposed by Kabir et al. [74]. They reported active electrocatalysts by consistent distribution of ionomer on carbon-based catalyst particles within the fibers using an electrospinning procedure. Nanofiber-based cathode was prepared by electrospinning a solution of pyrolyzed Fe-N-C catalyst (as carbon-based electrocatalyst), Nafion ionomer (as proton conducting ionomer), and poly acrylic acid (PAA) as the carrier polymer. Nanofiber electrode performance, mass-transport properties and accessibility to electrochemical active sites was improved in comparison to the painted electrodes in single-cell hydrogen/air fuel cell tests. Improved ionic accessibility to active sites and the increased proton or gas phase transport of the nanofiber electrode were attributed to the porous and interrelated structure of fibers and abundant of intra/inter-fiber voids.

In another work, He et al., developed Fe-free metal–nitrogen–carbon based catalysts for preventing destruction of carbon, ionomer, and the active sites of catalyst by Fe (Fenton reaction) in proton-exchange-membrane fuel cells [75]. Zinc-based zeolitic imidazolate frameworks were used for distribution of active CoN_4_ sites into carbon fibers. Active CoN_4_ sites were uniformly embedded in carbon nanofibers by electrospinning a dispersed mixture of cobalt-doped zeolitic imidazolate nanoparticles in polyacrylonitrile and poly(vinylpyrrolidone) polymers followed by a two-step heat treatment process. Carbonization of PVP polymer and evaporation of Zn from zeolitic imidazolate frameworks through thermal processes contribute to meso and micropores across the fibers. Enhanced electrode performance and durability in H_2_/air fuel cell tests were attributed to intrinsic activity and stability of single cobalt sites and to the porous structure of carbon nanofibers that facilitates access to active sites of the catalyst and improved mass transport of reactants (oxygen, proton, and water).

In another work, Pt-metal/carbon nanofibers and Pt-metal oxide/carbon nanofiber catalytic systems were proposed by Ponomare’s group as gas diffusion electrodes [76]. Platinated carbon nanofiber (CNF) composites were successfully used as cathodes in the membrane-electrode assembly under high-temperature polymer electrolyte membrane fuel cell experiment. In contrast to carbon black-based cathode durability and fuel cell performance being improved by distributing platinum-metal or platinum metal oxide electrocatalysts into carbon nanofibers due to the facilitated mass transport in highly porous CNF-based electrodes.

#### 2.3.2. Electrospun Electrolyte Membrane

Membranes are the main components in fuel cells. High electrical efficiencies in terms of thermal and mechanical stability and ion conductivity have been reported with electrospun nanofibrous-based proton conductive solid polymer electrolytes (proton exchange membranes) for effective proton transportation while maintaining fuel permeability at the lowest extent. As a representative example, Nafion ionomer-based electrospun nanofibers were fabricated by electrospinning polyvinylidene fluoride (PVDF) polymer solution followed by alkaline treatment that changes hydrophobic PVDF nanofiber surface to a hydrophilic surface suitable for Nafion impregnation [77]. In contrast to pure Nafion membranes mechanical properties were improved by distribution of Nafion in PVDF polymer matrix. The authors indicated that an appropriate content of modified PVDF polymer is crucial for reaching the maximum proton conductivity because proton transporting channels were blocked by increasing the content of modified PVDF. Prepared membranes with 15.1 wt.% modified PVDF reached the same proton exchange conductivity as plane Nafion membranes.

Liu et al. studied the role of hydrophobic electrospun nanofibers as substrate for boosting mechanical stability of anion exchange membranes for fuel cell application [78]. Silica coated electrospun PVDF nanofiber mats (SiO_2_@PVDF) were prepared and functionalized by grafting trimethyl-3-(trimethoxysilyl) propyl ammonium chloride. The positively charged mats were further modified with quaternized chitosan. Chitosan-based membranes has proven promising application in polymer electrolyte-based fuel cells and biofuel cells as biocompatible electrodes or solid polymer electrolytes [79]. Polymer electrolytes like quaternized chitosan with abundant of ammonium functional groups are capable of improving anionic exchange capacity of the membrane. The positively charged composite nanofibers provided conducting pathways for facile hydroxyl anions and exhibited higher hydroxide conductivity than that of pristine quaternized chitosan (0.041 S cm^−1^ (80 °C)) in single alkaline direct methanol fuel cell experiments (power density of 98.7 mW cm^−2^).

#### 2.3.3. Microbial Fuel Cells and Biobatteries

Energy generation from biomass is an alternative green energy source [80]. Microbial fuel cells are new generation of sustainable power supplies in which electricity is generated by enzymes or electrochemically active microorganisms exploiting stored energy in organic compounds [81].

Recently, bacteria-powered batteries or bio-batteries based on the principles of microbial fuel cells have gained attention for medical applications such as power sources for implantable medical devices (pacemakers, hearing aids, …), generating electricity from physiological fluids (blood, sweat, …), and for environmental applications especially in wastewater treatment. Electrode material and membrane interface with the bacteria biofilm play an important role in the proliferation of bacteria and effectiveness of microbial fuel cells [82]. Electrospun nanofibers with enhanced conductivity features due to their high specific surface areas and high porosities have the potential for improving electrode characteristics in bio-batteries. As a representative example, an ultrathin and flexible biobattery comprising highly porous electrospun monolithic cellulose acetate membrane with the ability to supply a power density higher than 3 μW/cm^−2^ was prepared by Baptista et al., for biomedical devices and biosensors [83].

Modification of carbon-based anode (carbon paper) with electrospun nanofibers of polyethylene oxide was carried out for boosting interfaces of biofilm with anode electrode [84]. Biofilm adhesion to the anode and proliferation of microorganisms was significantly improved compared to bare carbon paper anode because of the high porosity of the fibers and nano architecture of the pores capable of entrapping microorganisms. A high current density, (23.2 ± 0.1 mA/m^−2^), was obtained when honey was used as fuel in a single-chamber microbial fuel cell.

## 3. Energy Storage

### 3.1. Lithium-Ion Batteries

Li-ion batteries (LIBs) are the most popular rechargeable batteries and have been widely used in portable electronic devices over the past twenty years. The advantages of these batteries, such as gravimetric and volumetric energy density, slow self-discharge rates, good shape versatility, and no memory effects make them suitable in electric vehicles [85,86]. However, LIBs have limited application due to their large volume changes during cycling, unstable solid electrolyte interface layer, and poor power capability [87]. In order to improve the performance of LIBs for example increasing rate capability and cycle stability, 1D nanostructured materials have been introduced. Among 1D materials, electrospun fibers have attracted great interest for their significant advantages, such as controllable morphology, low cost, and high specific surface area [88]. LIBs are composed of anode, electrolyte, a cathode, and separators. In this section, the review is mainly focused on recent advances in LIB components, including nanofibrous anodes and cathodes.

#### 3.1.1. Anodes

Among numerous nanomaterials, carbon NFs are widely used as the anode in LIBs due to their easy availability, low cost, and long cycle life. However, carbon NFs have some drawbacks such as low energy density and rate capability and hence have limited their application. Therefore, various synthesis methods and materials are employed in anode materials for obtaining higher specific capacity and better electrochemical performance of LIBs. Yang et al. had prepared C/PAN NFs by the combination of electrospinning and thermal treatments. High reversible capacity and high rate capability of the nanofibers made them ideal candidate for the anode material of high-power LIBs [89].

In 2013, Zhang et al. studied the effect of nitrogen-containing groups on the performance of Li-ion storage and the capacities of carbon-based anodes in Li-ion batteries. For this purpose, they used polyacrylonitrile precursor to fabricate carbon nanofiber films by electrospinning as binder-free electrodes. The result showed that that Li ions can be stored not only between the graphene layers, but also at the defect sites created by nitrogen functionalization. An optimized carbonization temperature of 550 °C led to high nitrogen content and thus a high capacity of the electrode [90].

Recently, a simple coaxial electrospinning approach was adopted for the fabrication of Fe_3_O_4_@CNFs by using lyotropic cellulose acetate as the carbon nanofiber phase. Fe_3_O_4_@CNFs electrode showed high reversible capacities (RCs) of 773.6 and 596.5 mAh/g^−1^ after 300 cycles can be widely used for high performance energy storage materials [91]. In addition, Li et al. prepared a flexible TiO_2_@C/N composite nanofibers through electrospinning, sol-gel transcription, low-temperature solution precipitation and subsequent carbonization process. The results showed high initial capacity, good cycling stability and rating capability [92].

#### 3.1.2. Cathodes

**Figure 4:**
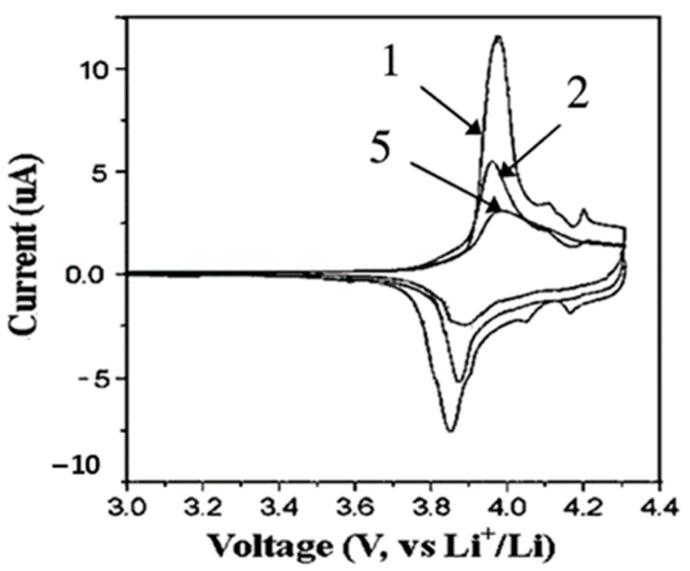
Cyclic voltammogram of diffusion and migration of Li ion in the LiCoO_2_ nanofiber electrode. Adapted from [95]. Copyright (2005), American Chemical Society.

Similar to anode materials, there are many cathode materials which have been synthesized by electrospinning method. Among these materials, LiCoO_2_ has a typical layered structure and it is widely used in commercial LIBs due to its high voltage, long cycle life, and high specific capacity [93]. LiCoO_2_ nanofiber electrode produced by electrospinning could enhance Li ion and electron conductivity due to shortening diffusion distance [94]. In 2005, LiCoO_2_ nanofibers were prepared by the combination of sol-gel and electrospinning method. The cyclic voltammogram curves (Figure 4) indicated faster diffusion and migration of Li ion in the LiCoO_2_ nanofiber electrode [95].

They then used MgO for encapsulating the LiCoO_2_ NFs through coaxial electrospinning. After 40 cycles, the core-shell fiber electrode retained 90% of its initial charge capacity but this value was only 52% for uncoated LiCoO_2_ nanofiber [96]. Liu et al. prepared LiCoO_2_ nanofibers with diameters 100 nm to 200 nm using electrospinning technique from a viscous solution of lithium acetate/cobalt acetate/PVP (polyvinylpyrrolidone). The LiCoO_2_ exhibited a high capacity of 140 mAh/g under high charge-discharge current density 0.6 C [97]. In another article, ZhiHui et al., have presented electrospun cellulose nanofibers with LiCl activation [98].

Other cathode materials that have been extensively studied were LiFePO_4_ and V_2_O_5_ because of their good cycling stability and high theoretical specific capacity. However, both of them lead to low rate capability and low electronic conductivity. In order to solve these problems, the use of electrospinning to produce nanofibrous LiFePO_4_ and V_2_O_5_ or their composites is regarded as the better way [99,100,101,102].

### 3.2. Lithium-Sulfur Batteries

**Figure 5:**
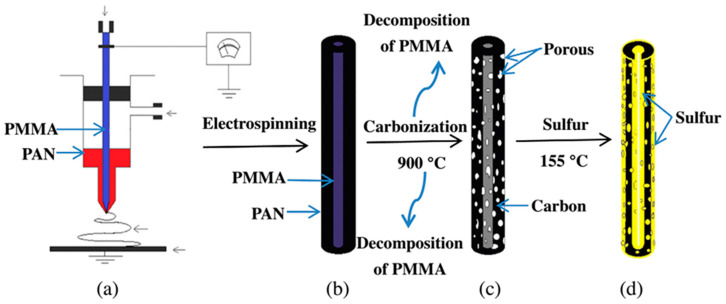
Schematic illustration of the synthesis processes of the S/MhMpCFs composite: (**a**) electrostatic spinning process; (**b**) as-spun fiber of PAN/PAMMA; (**c**) carbonized mesohollow and microporous nanofiber; (**d**) S incorporated hierarchically porous carbon nanofiber composite. Reprinted from [107], copyright (2014), with permission from Elsevier.

Lithium-sulfur (Li-S) batteries are the next generation of energy devices with low cost and environmental safety [103]. However, three major problems cause the poor commercial application of Li-S batteries. Firstly, sulfur and its products are ionic and electrical insulated that lead to the low power capability. Secondly, the diffusion of polar intermediate lithium polysulfides into the electrolytes. Finally, the high volume of sulfur cathode during cycling resulting in the decrease of cycling stability and durability [104,105]. In order to solve these problems, CNFs have been used as sulfur cathodes. Wang et al. has prepared ordered mesoporous carbon fiber for the first time by using resol as the carbon source and triblock copolymer Pluronic F127 as the template. The resulting ordered mesoporous carbon fiber sulfur (OMCF-S) composite with 63% S exhibited high reversible capacity, good capacity retention, and enhanced rate capacity when used as cathode in rechargeable lithium-sulfur batteries and provided good electrical and ionic pathways [106]. In 2014, mesohollow and microporous carbon nanofibers (MhMpCFs) were produced by a coaxial electrospinning with polyacrylonitrile (PAN) and polymethylmethacrylate (PMMA) as outer and inner spinning solutions (Figure 5).

PMMA decomposed in the carbonization process and induced the mesohollows and micropores. S fills fully in the micropores and fills mostly in the mesohollows via the thermal treatment, forming hierarchical structured-S/carbon fiber composite with 60 wt.% S. This sulfur/porous carbon fiber composite shows a maximum capacity of 815 mAh/g at 0.1 C after several activation cycles as a cathode material for Li-S batteries, and a capacity retention of 88% is obtained after 70 cycles with respect to the maximum capacity [107].

Gao et al., fabricated MCPs-PAN nanofibers by electrospinning the mixture of microporous carbon polyhedrons (MCPs) and PAN and used as cathode material in lithium sulfur batteries. The obtained S/MCPs-PAN nanofibers, with a suitable sulfur content as amorphous state inside MCPs and PAN nanofibers, presented the optimized electrochemical performance, including a high sulfur utilization, good capacity retention, and excellent rate capability [108]. Recently, a novel Bio-inspired poly(3,4-ethylenedioxythiophene):poly(styrene sulfonate)-sulfur@polyacrylonitrile electrospun nanofibers in the cathode of lithium-sulfur batteries have been reported. The electrochemical performance of lithium-sulfur batteries using poly(3,4-ethylenedioxythiophene): polystyrene sulfonatesulfur@polyacrylonitrile nanofiber incorporated sulfur hybrid cathode showed a significantly better cycle stability and excellent rate capability compared to the bare sulfur cathode owing to the adsorption effect of the nanofibers [109]. Tong et al. summarized the recent development and proposed the existing challenges and future prospects of carbon-containing electrospun nanofibers (CENFs), for the design and architecture of electrochemical components in Li-S energy storage systems [110].

### 3.3. Lithium-O_2_ or Lithium-Air Batteries

Among the energy storage devices rechargeable lithium-air batteries with the capability of higher theoretical energy density delivery than lithium-ion batteries have gained substantial attention [111]. Lithium-air battery technology works with a similar basic principle of hydrogen-powered fuel cells where proton replaced with Li^+^ ions. Lithium-air batteries consist of a lithium metal anode where metallic lithium is oxidized to lithium ions then lithium ions migrate through an aqueous or solid electrolyte toward a porous cathode and combined with oxide anions (produced by oxygen reduction) to form lithium peroxide (Li_2_O_2_) inducing electric current in the external circuit. Formation and decomposition of Li_2_O_2_ occurs during the discharge/charge cycles. The performance of lithium-air batteries is definitely affected by catalysts in air electrode. Developing effective bifunctional catalysts capable of efficient oxygen evolution and oxygen reduction reaction have caused diverse studies on cathode design. It has been shown that electrospinning is an appropriate method for embedding oxygen reductive materials into porous carbon-based fibers. Additionally, mesoporous structure of electrospun nanofibers provides facile gas diffused channels for increasing oxygen–electrolyte interface [20,112]. According to Tsou et al., electrochemical performance of oxygen electrode catalyst in Li-O_2_ batteries was improved by combining the bifunctional catalytic property of active catalyst with interconnected structural features of carbon nanofibers using iron phthalocyanine as the bifunctional catalyst covalently bonded to pyridine-functionalized electrospun N-doped carbon nanofibers as the carbon supports [113]. Zhao et al. showed that RuO_2_·nH_2_O clusters immobilized on the carbon nanofibers deliver higher specific capacity (4680 mAh/g^−1^) than pure carbon nanofibers and notable structural cycling stability were obtained [114].

In another work, perovskite type oxide LaCo_0.6_Ni_0.4_O_3_ incorporated carbon nanofibers was fabricated by electrospinning a PVP mixture contained La(NO_3_)·6H_2_O, Co(NO_3_)_2_·6H_2_O and Ni(NO_3_)_2_·6H_2_O (La/Co/Ni molar ratio of 5:3:2) followed by calcination [115]. Mesoporous LaCo_0.6_Ni_0.4_O_3_ nanofibers decorated with Co_3_O_4_ nanoparticles were prepared by a post hydrothermal procedure. Chemisorption of oxygen and desorption of O^−2^ were improved with the aid of uniformly Co_3_O_4_ nanoparticles distributed on the surface of nanofibers resulting in a better oxygen evolution and oxygen reduction performance when used as cathode in lithium air batteries. The prepared catalyst displayed high specific capacity of 11,288 mAh/g^−1^ and high coulombic efficiency of 85%, while retaining cycle stability after 116 cycles at high current density of 1000 mA/g^−1^.

### 3.4. Supercapacitors

**Figure 6:**
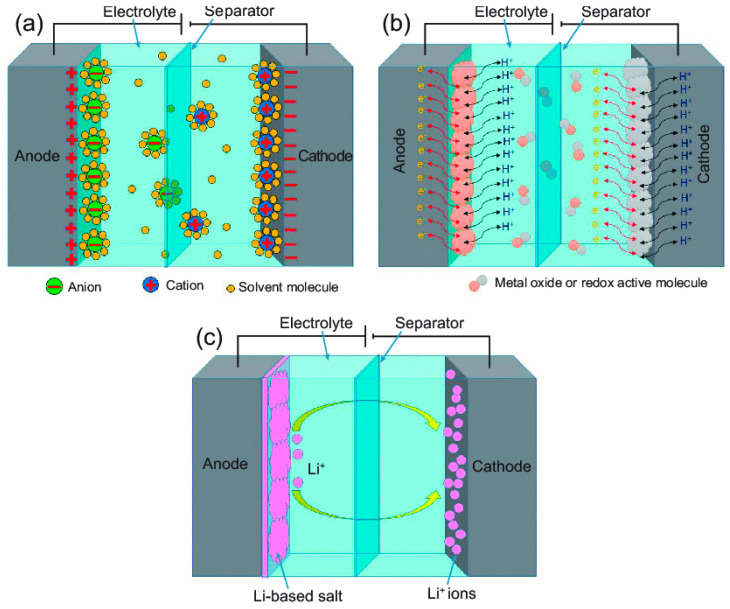
Schematic illustration of comparison between (**a**) an electrical double-layer capacitor, (**b**) pseudo-capacitor, and (**c**) hybrid supercapacitor. Reprinted from [116], under open access license from Oxford University Press.

Supercapacitors are among electrochemical energy storage/delivery devices, with high power densities and longer cycle efficiency in comparison to rechargeable lithium-ion batteries though their lower energy densities. They have been used in portable electronic devices, memory backup systems and auxiliary power units. The configuration is based on two electrodes immersed in an electrolyte and an ion permeable separator that divides the system in two parts (Figure 6). Regarding the mechanism of charge storage and delivery, supercapacitors were classified as electric double-layer capacitors (EDLCs), electrochemical pseudo-capacitors (or redox electrochemical capacitors) and hybrid supercapacitors. Electric double-layer capacitors (EDLCs) rely on electrostatic interaction between electrolyte ions and active materials of electrodes depending on surface area, dielectric constant of the electrolyte, the electrode material and the extent of separation between electrolyte ions and the active component of the electrode. Carbon based materials frequently used as active electrode materials in supercapacitors because of good electrically conductive features; electrochemical stability in harsh conditions and high surface area [116]. In pseudo-capacitor redox reactions between electrolyte ions and electrode materials (often metal oxides or electrical conducting polymers) at the interface between electrolyte and electrode surface is based on fast Faradaic charge transfer. Charge storage in hybrid supercapacitors is based on a combination of two mechanisms exhibiting higher energy densities without compromising energy power.

Electrospinning is a prospective technique for fabricating electrode materials that meet the requirements of nanomaterial electrodes for supercapacitors. Tunable surface area, pore size, thickness, and their potential for incorporation of active redox or pseudocapacitive materials—such as redox metal centers, metal oxides, metal sulfides, conducting polymers, etc.—have offered solutions for development of light-weight supercapacitors with high capacitance and low resistance [117,118]. In a comprehensive review, Liang et al. studied recent advancements in carbon nanofibers, carbon nanofiber-based composites, and transition metal oxide fibers as electrode materials in supercapacitors [118]. For instance, electrochemical performance of carbon nanofibers was improved as supercapacitor electrode materials by incorporating Fe_1−x_S-TiO_2_ nanoparticles into fibers. Fe_1−x_S-TiO_2_-CNFs composite material was fabricated by electrospinning and post carbonization process [119]. Fe_1−x_S was used as pseudo-capacitance material and its electrochemical properties coupled with double-layer capacitive characteristics of carbon nanofibers as well. Owing to the increased porosity and conductivity of the capacitive hybrid material, higher specific capacitance (138 F/g at the current density of 1 A/g^−1^) and a capacitance retention about 83% were achieved after 2000 cycles compared with plane TiO_2_ nanofibers.

**Figure 7:**
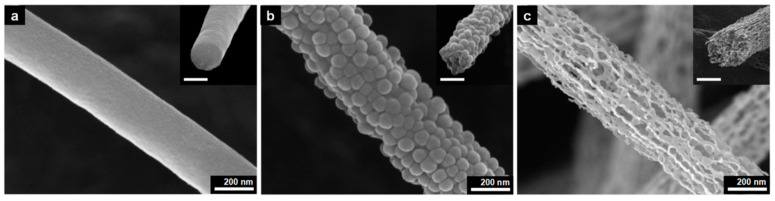
FE-SEM images of (**a**) pure carbon nanofiber, (**b**) PAN/silica composite carbon nanofiber, and (**c**) porous carbon nanofibers. Reprinted from [120], with open access use license.

In another study, Kim et al. demonstrated the role of porosity of carbon nanofibers on electrochemical performance of capacitive materials [120]. Silica nanoparticles embedded carbon nanofibers were prepared by oxidation and carbonization of electrospun composite PAN nanofibers incorporated different contents of silica nanoparticles then silica component was removed by hydrogen fluoride treatment (silica etching) (Figure 7). The final nano-porous carbon products showed increased specific surface area proportional to PAN/silica ratio. High areal capacitance (13 mF/cm^−2^ at a current of 0.5 mA/cm^−2^) for the highest porous carbon nanofibers (specific surface area of 391 m^2^/g^−1^, 500 wt.% PAN) was ascribed to facile mass transport (ions and electrons) through the interconnected pore structures which provides higher electrical double layer capacitance.

Jeon et al. designed fabrication of capacitive nanofibers by growth of RuO_2_ nanorods on electrospun carbon nanofibers via precipitation and recrystallization [121]. The hybrid composite was investigated as electrode materials in supercapacitors. Super-hydrophilicity induced in hydrophobic carbon nanofibers by RuO_2_ incorporation which is beneficial for electrolyte ion diffusion. Large surface area and mesopore/micropore structures were achieved along with uniform distribution of RuO_2_ electroactive sites which result in a higher specific capacitance (188 F/g^−1^ at a current density of 1 mA/cm^−2^) compared to plane carbon nanofibers.

High specific capacitance of 237 F/g^−1^ at the current density of 1 A/g^−1^ was observed for electrospun carbon nanofibers loaded with ZnFe_2_O_4_. Pseudocapacitive ternary metal oxide ZnFe_2_O_4_ incorporated carbon nanofibers were prepared by electrospinning followed by controlled annealing [122]. Improved in electrical conductivity due to the small diameters of carbon nanofibers coupled with consistent distribution of electroactive ZnFe_2_O_4_ nanoparticles into nanofibers were reported.

## 4. Environmental Application

Nowadays much attention has given to the reduction of ecological contaminations and removal of pollutants particularly from aquatic environment. Organic, inorganic, and biological pollutants discharging by natural and anthropogenic processes—including volcanic eruption, wildfires, biogenic sources, industrial and agricultural wastes, fertilizers and pesticides, animal manures, sewage sludge, and mining—impose serious health threat to humans, animals, and plants. Therefore, protection and remediation methods are urgently necessary for removing pollutants and handling environmental problems. Chemical separation and purification methods using electrospun nanofibrous membranes and implementation of these nanofibers has been attracting great interest with regard to devices requiring high performance filtration—such as water treatment filters, medical devices such as artificial kidneys, diagnosis devices, and biosensors.

Porosity, pore size, mechanical, and chemical stability of the nanofibrous materials play significant role in effective performance of these adsorbents. Chemical modification of the surface by incorporation of cationic or anionic polymers, nanoparticles, metal-organic frameworks (MOFs), and coordination polymers into nanofibers or by post treatments methods have been applied for enhancing the functional adsorption sites.

### 4.1. Oil Sorption

Oil spills along with other pollutants immiscible with water such as chemical leaks have caused serious environmental damage. Developing modified and selective adsorbent materials which meet the environmental requirements for separation of oil, grease, or other pollutants from water is receiving much attention since number of chemical, physical, and biological strategies have been reported for water treatments [123].

Fabrication of electrospun sorbents and membranes by electrospinning taking the advantage of surface chemistry and controllable pore diameter could be promising in oil-water separation in mixtures and emulsions. Electrospun nanofibrous materials including sorbents, membranes, and aerogels have been introduced for oil-water treatments [123]. Short and long-term effects of oil spill incidents on marine ecosystem have led to attempts for developing improved materials for efficient oil sorption. Oil spill treatment methods including oil spill containment booms, skimmers, in situ burning (for thin slicks), dispersants, bioremediation and sorbents have been developed for managing the spilt oil. Sorbents often used in final stages of oil spill cleanup. They are important materials in terms of complete removal of oil from the spilt area. It has been shown that sorbents with simultaneous oleophilic and hydrophobic properties displayed larger sorption capacity and lower tendency to generate the second contaminant.

**Figure 8:**
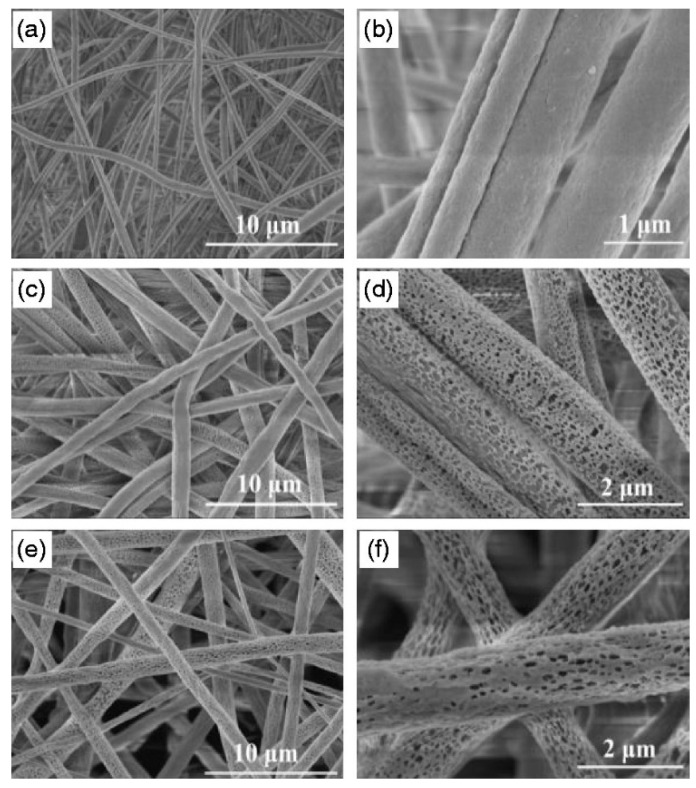
SEM images of nanoporous fibrous mats: (**a**,**b**) for polysulfone, (**c**,**d**) for polysulfone/poly (lactic acid) nanoporous fibrous mats, (**e**,**f**) for poly (lactic acid). Reprinted from [126], with open access license from SAGE.

Electrospinning is a suitable alternative technique for its easy and cost-effectiveness procedure in embedding special wettability resulting in well-controlled surface energy among the fibers, tuning the surface morphology and interconnectivity of inner voids. In recent years, electrospun nanofibrous polymeric sorbents have gained interest in oil sorption and water treatment techniques [124]. Oil sorption by fibrous sorbents predominantly is a combination of adsorption and capillary action (few are absorbents) [125]. Selective wettability, oil sorption capacity, morphology and buoyancy of the porous nanofibers are important factors affecting the efficiency of oil sorption. Nanometer porous morphology of the fibers and increased specific surface area has a significant effect on oil sorption capacity [126]. Figure 8 shows the scanning electron microscope (SEM) images of nanoporous fibrous mats of polysulfone/poly (lactic acid) in comparison with nanoporous fibrous mats of polysulfone and poly (lactic acid). Polysulfone/poly (lactic acid) and polysulfone nanoporous fibrous mats with higher specific surface areas showed better oil sorption capacity than poly (lactic acid) nanoporous fibrous mats.

Additionally, physical and chemical properties of the oil, temperature, climatic, and natural conditions of the affected site may also be considered for the sorbent to be practical in a real spillage [125,127]. Addressing the ecological safety of the sorbents, reusability, and effective recovery of the spilt oil should be considered. In a recent study, Liu et al., also investigated the effect of oil retention capacity (keeping oil encapsulated in the sorbent) of electrospun fibrous mats on the efficiency of the fibers [126]. Hydrophobic-oleophilic polymer-based nanofibers, carbon-based nanofibers, and composite nanofibers have been developed for oil spill remediation.

#### 4.1.1. Hydrophobic-Oleophilic Polymeric Nanofibers

Control of surface wettability by using special wettable polymers have been employed for oily water treatment methods. Hydrophobic (water contact angle (WCA) of >90°) and superhydrophobic materials (WCA of >150° and sliding angle of lower than 5–10°) are those with low surface energy and low affinity to water whilst super oleophobic surfaces (oil contact angle (OCA) of >150° and WCA <10°) have high affinity to water. Superhydrophobic-super oleophilic materials have the ability to repel water molecules while allowing oil to be passed or absorbed into their porous structures. Numerous studies focused on the fabrication of electrospun superhydrophobic–super oleophilic polymeric surfaces and nanofibrous membranes with the selective wettability to filter or absorb oil from oil/water mixtures under low pressure [128,129]. PTFE Teflon (polytetrafluoroethylene)-coated stainless-steel mesh having simultaneously superhydrophobic (156.2° ± 2.88°)-super oleophilic (diesel oil CA of about 0° ± 1.3°) property is one of the pioneering studies in this area reported by Feng’s group in 2004 [130]. The superhydrophobicity–superoleophilicty was ascribed to nanostructured craterlike surface morphology as well as chemical composition of the material.

#### 4.1.2. Composite Nanofibers for Oil Sorption

In composite polymeric nanofibrous materials characteristics of the polymeric sorbent such as mechanical properties, surface wettability, surface roughness, energy, and surface-to-volume ratio can be improved or modified by incorporation of other materials into polymeric nanofibrous structure [123].

**Figure 9:**
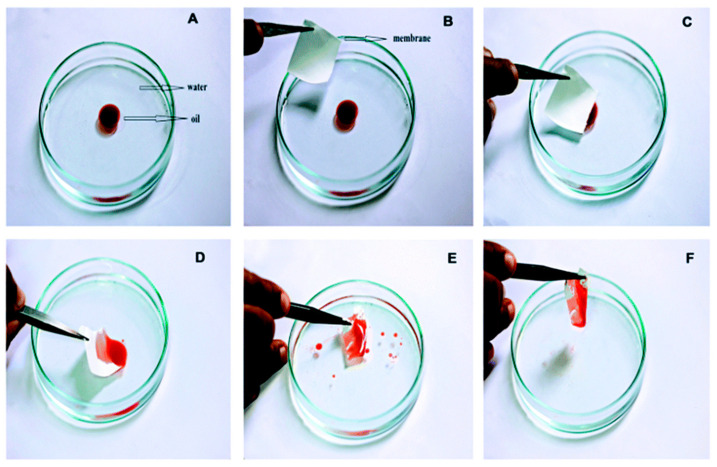
(**A**–**F**) Selective sorption of oil from contaminated water surface using hybrid membrane of bee’s wax and polycaprolactone [132] published by The Royal Society of Chemistry.

Polyethylene and polyvinyl chloride-blended polystyrene composite nanofibrous sorbents have synthesized by Alabi’s group by mixing low-density polyethylene and poly (vinyl chloride) components with a polystyrene (PS) matrix. The prepared hydrophobic sorbents exhibited high affinity towards the removal of oil spills with five times higher sorption efficiency than that of commercial polypropylene fibrous sorbents (112 and 119 g/g oil uptake capacity, respectively) [131]. In another study aiming at fabrication of reusable and environmentally friendly sorbents, nanofibrous membrane from beeswax and polycaprolactone fabricated by electrospinning (25 wt.% beeswax (water contact angle of about 153° ± 2° and oil sorption capacity of 16.95–31.05 g/g^−1^ in different oils). The superhydrophobic-super oleophilic electrospun nanofibrous membrane showed high efficiency in selective oil/water separation. Bee’s wax as a natural polymer incorporated into electrospun polycaprolactone membrane for inducing superhydrophobicity (Figure 9) [132].

Z. Jiang et al. prepared magnetic composite nanofibrous mats using oleophilic and hydrophobic polymers such as polystyrene/polyvinylidene fluoride for oil-in-water separation (oil sorption capacity of 35–46 g/g) [133].

Incorporation of magnetic Fe_3_O_4_ nanoparticles on or in the nanofibers enables magnetic recovery of the mats using an external magnet and increases the mechanical strength of the composite nanofibrous mats compared to that of neat polystyrene mat.

#### 4.1.3. Carbon-Based Nanofibers CNFs

**Figure 10:**
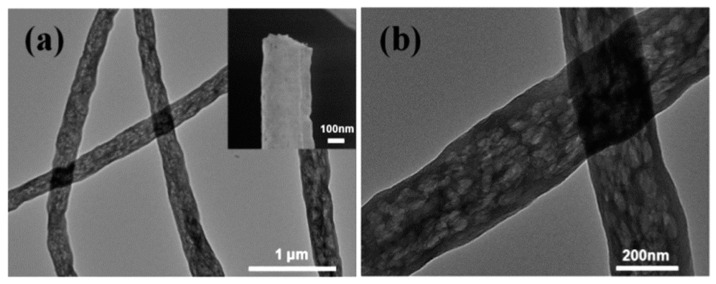
(**a**) Low-magnification TEM image of macroporous electrospun carbon nanofibers; the inset shows the SEM image of the surface of the single fiber; (**b**) high-magnification TEM image indicating inter-connected macropores. Reproduced from [136] with permission from The Royal Society of Chemistry.

**Figure 11:**
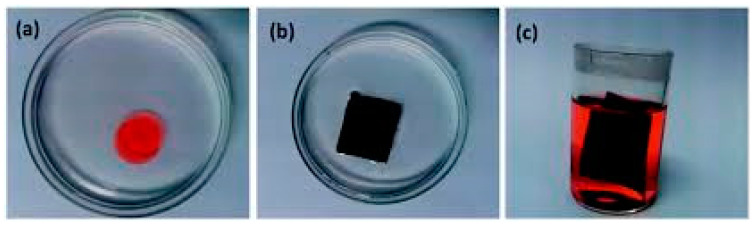
Oil sorption by macroporous electrospun carbon nanofibers, (**a**) before and (**b**) after successful sorption. Pump oil was colored with Sudan III for clear visual observation. (**c**) Recovery of the absorbed oil by immersing into dichloromethane. Reproduced from [136] with permission from The Royal Society of Chemistry.

Carbon nanofibers (CNFs) are a subgroup of one-dimensional nanomaterials with the average diameters in the range of 50–100 nm. They are one of the most effective reinforcement materials for improving the mechanical properties of polymeric composites [134]. Electrospinning is the most common procedure for the fabrication of carbon nanofibers. Electrospun carbon nanofibers have smaller diameters than those produced by the other methods. Application of carbon nanofibrous films in oily/water treatments is advantageous over organic polymer-based sorbents for being stable in harsh conditions (acidic or basic media). However, very few studies have been reported on fabrication of carbon nanofibers for oily water treatments [135]. A pioneering work using electrospun nanofibers conducted by Liu et al., introduced a flexible and recyclable electrospun carbon nanofibrous film for oil-water separation (water contact angle of 155.3°, oil uptake capability of 138.4 g/g^−1^ for silicone oil) [136]. Electrospun carbon nanofibers with macroporous structure inside the nanofibers was synthesized by a post thermal treatment and sublimation of terephthalic acid within electrospun terephthalic acid-polyacrylonitrile composite nanofibers. The macroporous structure inducing flexibility and superhydrophobic–superoleophilic property of the nanofibers (Figure 10 and Figure 11).

In another work, Y.-Z. Lin et al., prepared carbon nanofiber/graphene oxide aerogels (CNF/GOAs) as an efficient sorbent for oil uptake with high sorption capacity (120–286 wt./wt.) and excellent recyclability that ascribed to low density (2–3 mg/mL) of the aerogel. A liquid assisted collection-electrospinning procedure combining with freeze drying and thermal stabilization was employed for the preparation of mentioned nanofibrous aerogel [137]. Carbon-based aerogels are promising materials for oil/water treatments owing to their ultra-low density and highly porous structures.

### 4.2. Oil/Water Separation

Traditional methods for oily water treatment are gravity separation, adsorption-based technologies, flocculation, coagulation and biological treatments. Fouling, secondary pollutants, low selectivity and inefficiency for separation of emulsified oil/water mixtures are limitation of these procedures. A possible solution could be the use of filters and membrane technology especially in the field of demulsification [138,139]. Nanosized pore diameters in membranes makes them capable of separating emulsions while membranes have lower efficiency in separation of large scaled amounts of oily water compared to meshes (pore size in meshes ~50 µm). Electrospinning has been used as a successful fabrication approach for efficient oil/water separation membranes [140,141].

Selective adsorption (or in a few cases absorption) of either oil or water while repelling the other one is considered to be promising for energy considerations. Developing oil-selective and water-selective membranes or stimuli-responsive (pH, salt concentration, or temperature-responsive) membranes with switchable control for oil/water separation [142] are efficient approaches that drawing attention in recent years. Many methods based on developing superhydrophilic/superoleophobic membranes (water removing type), superhydrophobic/superoleophilic membrane (oil removing type), superamphiphilic membrane with the ability to separate oil-in-water and water-in-oil emulsions, smart controllable separation membranes (responsive membranes) with a switchable superwettability have been developed in this regard [143,144]. It has been demonstrated that superhydrophilic/superoleophobic membranes are suitable for separating large-scaled oil-in-water emulsions due to their lower fouling performances [139], while superhydrophobic/superoleophilic membrane separates water-in-oil emulsions. It should be mentioned that permeability, hydrophobicity, surface charge, functionality, morphology, and interconnected voids among the network are important factors affecting the separation efficiency.

**Figure 12:**
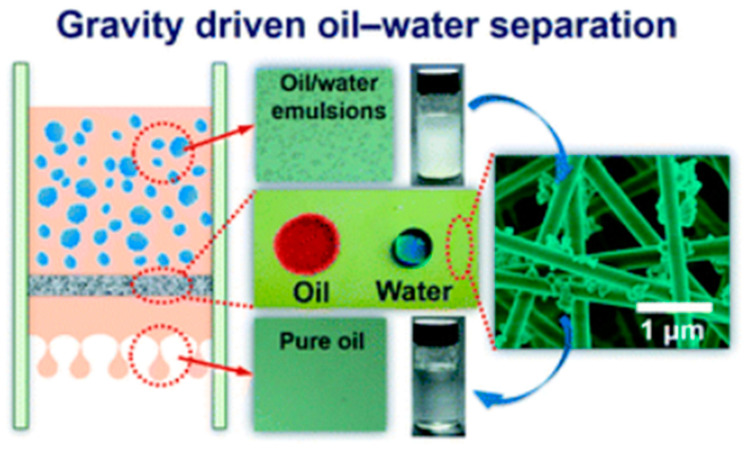
Gravity-driven separation of surfactant-stabilized water-in-oil microemulsions by fluorinated polybenzoxazine (F-PBZ) coated functionalized silica nanofibrous membranes (F-SNF/Al_2_O_3_). Reproduced from [145] with permission from The Royal Society of Chemistry.

In this context, modified electrospun fibrous filters and membranes offer potential application for the separation of oil/water mixtures and emulsions due to interconnected pore network. Fluorinated polybenzoxazine (F-PBZ) coated functionalized silica nanofibrous membranes (F-SNF/Al_2_O_3_) has been developed for effective gravity-driven separation of surfactant-stabilized water-in-oil microemulsions (high flux of 892 L/m^−2^/h^−1^, WCA of 161° and an OCA of 0°) with a great antifouling tendency (Figure 12). The superhydrophobicity and superoleophilicity of the reported membrane was attributed to the hierarchical structure and increased surface roughness and surface area arising by Al_2_O_3_ NPs [145].

In a recent study, a magnetite intercalated polystyrene nanofibrous hybrid membrane was prepared by electrospinning. The prepared nanofibrous membrane proposed by Kim and El-deen’s group [146] showed superhydrophobic/superoleophilic property for a gravity driven oil/water separation (ultrahigh flux 5000 L/m^−2^/h^−1^, water contact angle 162°, 98.5% reusability for 50 cycles). Recently, electrospun polyacrylamide/polystyrene (PAM/PS) composite fiber with porous surface was utilized for fabrication of a fibrous membrane showing high efficiency for oil–water separation (water contact angle of 157.6° ± 2.5°, high oil-uptake capacity for edible and industrial oils about 154.5–202.2 g/g and good stability in 20 cycles) [147].

### 4.3. Heavy Metal Ion Adsorptions (Metal Ion Removal)

Environmental contamination with heavy metal ions leads to adverse changes due to their non-degradability and bio-accumulative nature. Even trace amounts of many heavy metal ions could cause serious health problems. For instance, inorganic arsenic, cadmium, lead, and mercury are among the ten chemicals/groups of chemicals of major public health concern according to World Health Organization (WHO) [148]. Studies have shown that these metal/metalloids associated with numerous diseases such as development of autism spectrum disorder in children [149], skin pigmentation change and lesions [150], liver and renal diseases [151], neural diseases and anemia [152].

Heavy metal ion remediation including precipitation, adsorption, biosorption, and membrane-based technologies have been employed for removal of heavy metal ions from water [153]. Adsorption-based technologies are considered as the most efficient method regarding the cost and the potential of desorption and recovery of the adsorbed ions. Moreover, they failed for large scale treatments. As a representative study, a superhydrophilic membrane (WCA of 0°) was prepared by cellulose acetate and poly-(methacrylic acid) via the electrospinning procedure. The core-shell nanofibers comprised hydrophilic poly-(methacrylic acid) shell and a hydrophobic cellulose acetate core were capable of selective removal of Pb (II) ions in a mixed ion solution (adsorption capacity of Pb (II) 146.21 mg/g^−1^) [154].

In other study, combined properties in MOF/nanofiber hybrid membranes was used by Peng et al., for removal of Cu^2+^ ions in a dynamic adsorption. ZIF-8 (zeolitic imidazolate framework) nanoparticles were growth on electrospun polyacrylonitrile (PAN) nanofibrous membrane (ZIF-8/PAN NF) by hot pressing method. The hybrid membrane showed good performance (flux rate, 12,000 L/(m^2^/h)) for elimination in a dynamic adsorption (29.2% removal rate of Cu^2+^ in 4 min in adsorption process and 34.1% with combination of adsorption and electrochemistry functions). In this work, the effective removal of Cu^2+^ was ascribed to improved surface chemistry caused by high specific surface area, and abundant active metal sites of ZIF-8 nanoparticles on nanofibrous surface [155].

### 4.4. Adsorption of Organic Compounds

Persistent organic pollutants (POPs) as a group of xenobiotic compounds [156] are of special health concern because of their highly toxic potential. These noxious chemicals are resistant to natural degradation processes, capable of bio-magnifying and bio-accumulating in ecosystems. They are byproducts of industrial practices or release through natural sources like forest fires and volcanoes. Most common persistent environmental pollutants are polycyclic aromatic hydrocarbons, flame retardants, surfactants, dioxins, and dioxin-like compounds such as polychlorinated biphenyls and organochlorine pesticides (like DDT), polychlorinated dibenzo-p-dioxins and polychlorinated dibenzofurans [157,158]. In recent years, progress in electrospun nanofibrous materials have resulted in several efficient adsorbent-based methods for removal of diverse organic pollutants from environmental compartments (soil, water, and air) [159,160,161].

As an example, Ehteshami et al., synthesized electrospun polyamide/graphene oxide nanocomposite as a fiber coating for a solid-phase microextraction (SPME) of polycyclic aromatic hydrocarbons from water. The nanofibrous composite was coated on stainless steel wire by electrospinning a solution of polyamide (PA) polymer containing dispersed graphene oxide (GO) nanoparticles. Graphene oxide has been widely investigated as an appropriate coating material due to its specific properties like porosity, high surface area, and hydrophilicity. Incorporation of GO nanoparticles in the coating resulted in enhanced surface area of nanocomposite and adsorption ability. The extraction mechanism proposed to be a π–π and hydrophobic interaction between benzene rings of polycyclic aromatic hydrocarbons with the PA/GO nanocomposite [162].

Another study presented reusable electrospun polycyclodextrin (poly-CD) nanofibrous membrane as an efficient sorbent for extraction of five polycyclic aromatic hydrocarbons (acenaphthene, fluorene, fluoranthene, phenanthrene, and pyrene) from water (equilibrium sorption capacity of (q_e_) 0.43 ± 0.045 mg/g). Poly-CD nanofibrous membrane was prepared by the electrospinning a hydroxypropyl β-CD solution in the presence tetracarboxylic acid-functional cross-linker and sodium hypophosphite as an initiator followed by a heat treatment step [163].

Borhani et al. synthesized molecularly imprinted sol-gel nanofibers using sol-gel process and the electrospinning method for adsorption of bisphenol A (BPA) in a water sample. Bisphenol A is an endocrine disruptor with possible health hazards. Effect of the amount of 3-aminopropyltriethoxysilane (functional monomer), acid (initiator), water and nylon 6 (polymer solution) were investigated for the adsorption capacity of molecularly imprinted NFs. The optimum molar ratio of 3-aminopropyltriethoxysilane: acid: water was 1:2:9 and 12 wt.%, nylon 6 polymer solution for extraction of BPA from water (saturated binding capacity of 115.1 mg/g^−1^ for molecularly imprinted NFs, higher than that of non-imprinted polymer nanofibers (46.82 mg/g^−1^)). The nanofibers were successfully reused for five times without any changes in adsorption capacity [164].

### 4.5. Photocatalysis

Environmental pollution caused by industrialization and urbanization threaten the ecosphere and have caused serious ecological and environmental difficulties. Treatment of environmental pollution harnessing light irradiation is a sustainable route to eliminate or reduce the adverse impact of pollution on the environment. Nanocomposite materials with photo-catalytically active moieties embedded into/onto electrospun nanofibers improve the stability and reusability of the catalysts [165] and have demonstrated great role on supporting and preventing agglomeration of photocatalyst nanoparticles during photocatalysis [166]. In one study, for example, recovery and photocatalytic performance of semiconductor nanoparticles of silver phosphate were enhanced via surface functionalization of electrospun PAN nanofibers with sulfate anions-doped silver phosphate nanoparticles [167]. The application of nanofiber-based semiconductors for degradation of methylene blue (MB) and rhodamine B (RhB) dye solutions were evaluated under visible light irradiation. In another representative study, a highly visible-light responsive photocatalyst was successfully synthesized by Liang’s group [168]. Electrospun PAN nanofibers incorporated-1.9%-MoS_2_/g-C_3_N_4_ was prepared via electrospinning process and exhibited well distribution of nano catalyst particles on the PAN surface compared to agglomerated non-supported MoS_2_/g-C_3_N_4_ composite catalyst. Compared with bulk g-C_3_N_4_ and bulk MoS_2_/g-C_3_N_4_ photocatalysts, enhanced photocatalytic degrading of Rhodamine B (RhB) and methylene blue (MB) dye solutions under visible light irradiation (λ > 420 nm) along with good stability and reusability of the fibers was achieved. The improved photocatalytic performance was ascribed to synergistic effect between three components and the inhibited recombination of photogenerated charge carriers due to the well distribution of MoS_2_/g-C_3_N_4_ particles on one-dimensional structure of the nanofibrous mat which helps separation of photoelectrons and holes.

**Figure 13:**
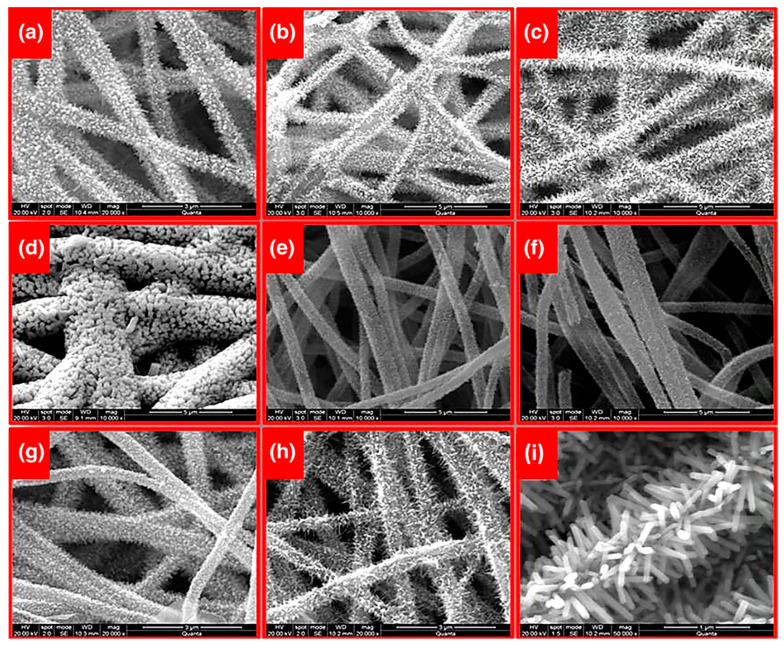
SEM images of hexagonal nanorods of La-doped ZnO grown on the surface of PAN nanofibers under different conditions (**a**–**c**) changing treatment time, (**d**–**f**) changing treatment temperature and (**g**–**i**) different La contents. Reprinted from [170], with permission from WILEY, copyright (2019).

Another study in this area reported the thermal and mechanical stability improvement of the nanofibers when electrospun polyurethane nanofibrous membranes loaded with reduced graphene oxide-titanium dioxide composite nanoparticles (rGO-TiO_2_) [169]. Visible-light-driven photocatalytic degradation of methylene blue dye was investigated and increased degradation efficiency with increasing the rGO-TiO_2_ content in the membranes was attributed to the facile diffusion of dye molecules to the photo-catalytically active moieties on the membrane due to the lower fiber diameter and higher surface area of the resulted rGO-TiO_2_-decorated membranes. Pure water flux and fouling resistance of the membrane compared to pure polyurethane membranes was improved as well and ascribed to the hydrophilic property of rGO-TiO_2_ component incorporated in nanofibers. Lakshmi et al. studied the growth of lanthanum-doped ZnO nanocrystals on electrospun PAN nanofibers via hydrothermal post modification process [170] (Figure 13). Effective performance of the catalyst in photocatalytic degradation of methyl parathion (an organophosphorus-based pesticide) was proved.

### 4.6. Nanofibrous Membranes in Water Treatment

Water and wastewater treatment using conventional gravity-driven separation methods including polymer-coated meshes and membranes, porous polymeric membranes and inorganic materials [142] have been used wieldy during the past two decades. The common drawbacks associated with these materials despite their good mechanical and chemical properties, is fouling and failure in the long-term water treatment processes, limited flux and low selectivity. Membrane treatments have emerged as a prominent way for filtration-based methods. Pressure driven membrane-based technologies employ a hydrostatic pressure difference to force water through a semi-permeable membrane. Nanofibrous mats and membranes by electrospinning benefits from the potential of fabricating designed materials with improved performance in filtering applications.

**Figure 14:**
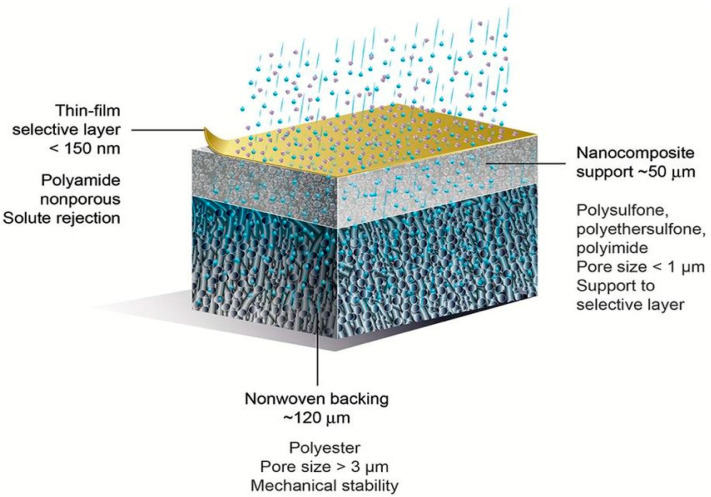
Schematic representation of a thin-film composite (TFC) membrane, and typical characteristics of its layers. Reproduced from [176], with open access license.

Regarding the membrane pore size and the measure of the operating pressure, microfiltration, ultrafiltration, nanofiltration, reverse and forward osmosis processes have been developed in water treatment processes for obtaining water that can be used in various zones such as agricultural irrigation [171], or municipal or potable use. Additionally, due to their large equivalent/apparent pore sizes (>0.5 µ), as-prepared electrospun nanomaterials are generally used as microfiltration media for pretreatment applications. Thinner diameters of nanofibers may sacrifice the mechanical strength of the membrane especially at higher pressure driven methods [172]. Functionalization of conventional polymeric membranes with nanomaterials (nanoparticles, nanofibers, nanotubes, nanosheets, or) leads to mechanical improvements, selective solute rejection, improved antifouling, and flow rate in nanocomposite membranes. Thin film nanocomposite membranes (TFNs) are a new class of thin film composite membranes (TFCs) composed of three layers: a nonwoven fabric as backing and an active layer that is a thin selective polymeric film (<150 nm) supported on a middle layer comprising a porous substrate. A schematic representation of a thin-film composite membrane is shown in Figure 14. Electrospun nanofibrous materials have been used as the mid-support layer in TFN membranes and offer a number of advantages over their nonwoven counterparts [173,174]. Thin film nanocomposite membranes (TFN) have gained significant academic attention in high pressure driven filtration like reverse and forward osmosis and nanofiltration [175].

Preparation of surface-functionalized and modified nanocomposite nanofibrous membranes can perform well in developing water treatment solutions [177]. Considering the environmental aspects, it is also apparent that significant attention should also be made to the recyclability of this membranes.

#### 4.6.1. Microfiltration

Microfiltration membrane with a mean pore size of 0.1–10 micron [178] and an applied pressure of 0.2–5 bar are capable of rejecting particulates that are greater than their pore size (including many microorganisms) [178,179] through a size-sieving mechanism [178]. Microfiltration membranes are usually used in pretreatment processes due to their lower operational pressure and low energy consumption. Menkhaus and Fong’s group emphasized the promising role of electrospun nanofiber membranes regarding flux value and antifouling property in microfiltration. They investigated the effect of fiber diameter, porosity, and thickness of the electrospun polyacrylonitrile (PAN) nanofiber membranes on removal of contamination in the ranges of 0.1–0.2 micron. It was demonstrated that hot-pressing of as-electrospun nanofiber membranes leads to less porous membranes and as a result improved rejection fraction and higher microfiltration performance were achieved [180].

**Figure 15:**
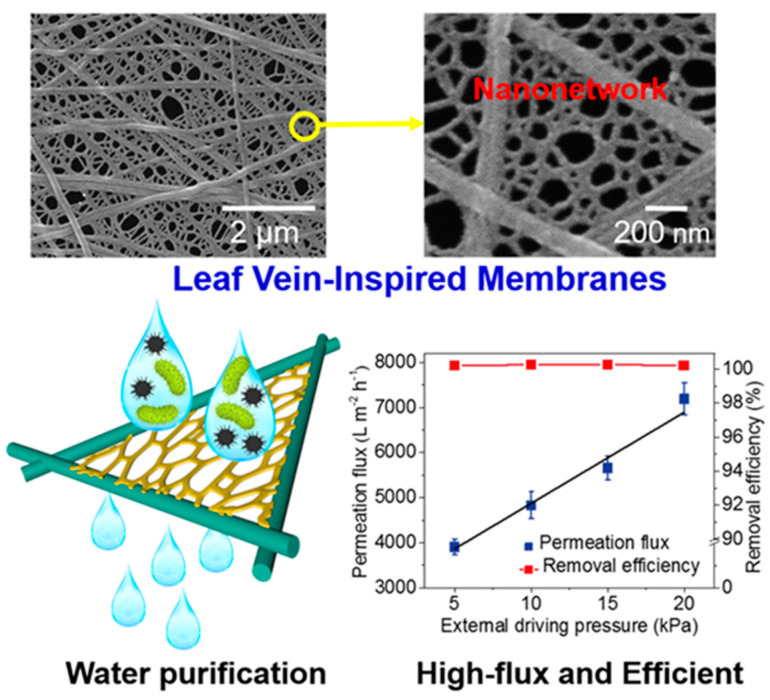
Leaf vein-inspired microfiltration membrane comprising nanonetwork polyacrylonitrile on electrospun polyamide nanofibers. Reproduced from [181] with permission from The Royal Society of Chemistry.

Si and Ding et al. synthesized a nanonetwork microfiltration membrane resembling leaf vein films by combining vapor-induced phase separation with electrospinning. They suggested a tightly, hydrogen bounded nanoscale network of polyacrylonitrile (PAN) (~45 nm fiber diameter) as minor veins on electrospun nanofibers of polyamide (PA) as main veins (Figure 15) [181]. Prepared porous membrane with small pore size of 0.19 μm, successfully retained pollutants including submicron sized TiO_2_ particles (size of ~0.3 μm, permeation flux 3907 L/m^−2^/h^−1^, applied pressure 5 KPa) and *Escherichia coli* (*E. coli*) as model pollutants.

**Figure 16:**
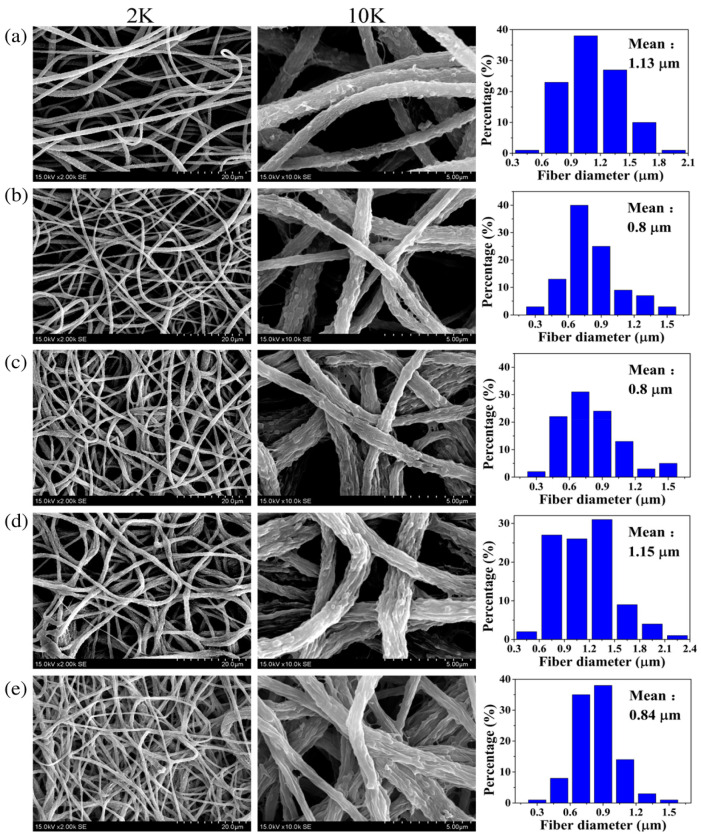
SEM images of (**a**) pristine PVDF membrane P0 and PVDF/PVA-blended membranes with different PVA contents of 5, 10, 15, and 20 wt.% denoted as (**b**) P1, (**c**) P2, (**d**) P3, and (**e**) P4 membranes at 2 K (left) and 10 K (middle) magnification and the fiber diameter and its distribution (right). Reprinted from [182], with permission from WILEY, copyright (2019).

Studies showed that antifouling property in hydrophobic polymer membranes could be improved by blending with hydrophilic polymers. In one study, intrinsic fouling features of hydrophobic polyvinylidene fluoride (PVDF) was solved by hydrophilic modification of PVDF membrane [182]. Li et al. reported the fabrication of polyvinylidene fluoride/poly (vinyl alcohol)-blended nanofiber membranes via a conventional electrospinning followed by a glutaraldehyde crosslinking treatment (average pore size 3–4 μm, thickness 200 ± 20 μm). Water flux and antifouling characteristics of PVDF membrane was improved by blending PVA in PVDF matrix. Figure 16 shows the SEM images of pristine PVDF membrane P0 and PVDF/PVA-blended membranes with different PVA contents of 5, 10, 15, and 20 wt.% denoted as P1, P2, P3 and P4, respectively. The PVDF/PVA-blended membrane containing 15% PVA showed a high pure water flux of 4.59 × 10^−4^ L/m^−2^/h^−1^ and an equilibrium particle suspension flux of 0.545 × 10^−4^ L/m^−2^/h^−1^ under extremely low pressure (0.025 MPa).

#### 4.6.2. Ultrafiltration (UF)

As the permeability decreases from microfiltration to ultrafiltration membranes, the operating pressure increases to guarantee a sufficient flow of liquid stream (the flux). Ultrafiltration membranes (pore size around 0.1 micron to 0.01 micron) requiring an applying pressure of 1–10 bar [178,179] are suitable for separation of macromolecular sized particulates such as dissolved large natural organic molecules, bacteria, protozoa, and some viruses from water. This kind of filtration have been successfully used for the separation of small amounts of emulsions that cannot be treated by conventional treatment procedures [183].

**Figure 17:**
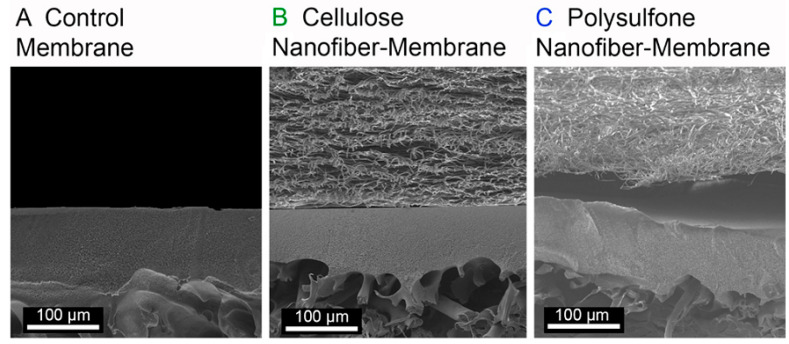
SEM micrographs of cross-section of a (**A**) control membrane, (**B**) cellulose nanofiber-membrane, and (**C**) polysulfone nanofiber-membrane. Reprinted with permission from [184], copyright (2017), American Chemical Society.

**Figure 18:**
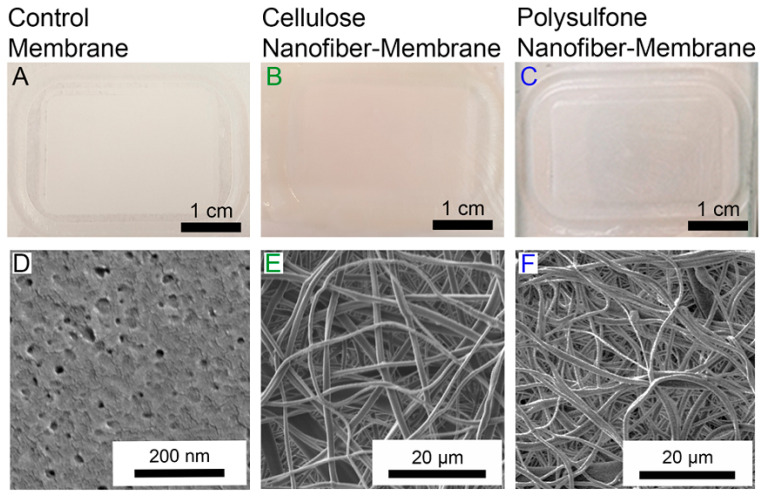
Digital images and SEM images of the (**A**,**D**) control, (**B**,**E**) cellulose nanofiber-membranes, and (**C**,**F**) polysulfone nanofiber-membranes. Reprinted with permission from [184], copyright (2017) American Chemical Society.

Schiffman et al. displayed the enhanced performance of ultrafiltration membranes by electrospun nanofibers. Composite ultrafiltration membranes with highly porous nanofiber layers of hydrophilic cellulose (WCA 0°) or robust hydrophobic polysulfone (WCA 110 ± 5°) [184] were evaluated in their research. An electrospun cellulose layer or a polysulfone nanofiber layer physically pressed on top of a commercial Biomax™ polyethersulfone ultrafiltration membranes (EMD Millipore, Billerica, MA, molecular weight limit of 100 kDa, WCA 41° ± 20°) as the base membranes. SEM images of cross-section of the control, cellulose nanofiber-membranes, and polysulfone nanofiber-membranes are shown in Figure 17 and Figure 18. The assembled composite ultrafiltration membranes showed improved permeability and fouling resistance in dead-end stirred cell experiments on an applied pressure of 2 bar. The size selectivity of the membranes did not change by incorporation of either cellulose or polysulfone nanofiber layers.

In another article, Wang et al. proposed the improved potential of membranes made of thermoplastic nanofibers and skeleton nanofibers in ultrafiltration upon hot-pressing [172]. Hot-pressing of polyacrylonitrile/poly (vinylidene difluoride) PAN/PVDF electrospun hybrid nanofibers at temperatures above the melting point of PVDF (10 MPa and 180 °C) resulted in a decrease in porosity of the nanofiber membrane from ~85.5 vol.% to ~16.7 vol.%. The decreased porosity was due to the partially melting/softening of nanofibers. The synthesized membranes exhibited high flux values higher than that of commercially available ultrafiltration membranes due to the reduced porosity. The hybrid membranes showed complete rejection of polystyrene particles larger than 20 nm and 40–80% for proteins (bovine serum albumin and bovine γ-globulin) depending on the protein type and the hot-pressing pressure under the applied pressure of 4 bar. After hot-pressing the overall morphology of membrane retained because of PAN resistance to melting/softening during the hot-pressing process. Antifouling features in ultrafiltration membranes can be improved using thin-film composite (TFC) membranes with hydrophilic coating layers. Bahmani et al., used a thin-film composite (TFC) membrane with an ultrafiltration membrane for removal of arsenate ions from water. The membrane composed of an electrospun polyacrylonitrile nanofibrous scaffold on a nonwoven polyethylene terephthalate (PET) substrate as support and a thin hydrophilic polyacrylonitrile (PAN) coating as the top layer. Highly porous TFC membrane showed a high flux about 172–520% higher than the UF membrane and a superior efficiency in arsenate rejection more than UF membrane (1.1–1.3 times) [185].

#### 4.6.3. Nanofiltration

Pressure-driven membranes with a mean pore size around 0.001 micron and applied pressure of 10–15 bar [178,179] retain dissolved small organic molecules, all viruses, synthetic dyes, mono- and multi-valent ions [178]. Therefore, nanofiltration have been applied widely for desalination of low salt contents [186], water softening, pretreatment of feed stream in high performance desalinations and antimicrobial processes in food and pharmaceutical applications [187]. Nanofiltration separation mechanism is based on sieving mechanism [188] and charge effects (relying on Donnan exclusion) [178]. The mechanical strength of the membranes decreases along with the decrease in diameters of the nanofibers especially in high pressure driven methods like ultra-, nanofiltration, and reverse and forward osmosis [172]. In order to achieve mechanical stability as well as higher water flux and higher fouling resistance in high-pressure driven filtrations, thin film nanocomposite membranes (TFN) are gaining attention as a new trend. Electrospun nanofibers hold great potential for improving the performance of TFC membranes rather than conventional substrate as the support. It has demonstrated that smaller fiber diameter and pore size of membranes lead to higher performance though at the expense of water flux [189].

Liu et al. synthesized a novel TFC membrane consisting of electrospun polyvinylidene fluoride (PVDF) nanofibers as the support layer with a thin polyamide (PA) film on the top of nanofibers through interfacial polymerization [190]. Authors showed that the pore size and porosity of the support membrane play an important role on the formation of top active layer. Therefore, during the electrospinning tetrabutylammonium chloride (TBAC) was used for modifying the porosity and morphology of the PVDF nanofibers and a tree-like electrospun polyvinylidene fluoride nanofibers were produced. The significant water flux and high salt rejection rates (97% against MgSO_4_ solution and 76% against NaCl solution) were attributed to formation of an interface between the PVDF and PA matrix due to small amounts of tree-like branched structures that inserted into the polyamide layer and acting as directed water channels. In another study, composite electrospun polyvinylidene fluoride (PVDF)/poly(vinylpyrrolidone) (PVP) nanofiltration membranes containing chitosan/activated carbon/silver nanoparticles were synthesized by Nakhowong’s group. They showed that incorporation of Ag nanoparticles with intrinsic antibacterial characteristics into composite membranes improve the antibacterial activity of membranes against *E. coli* and *S. aureus* [191]. Zhijiang et al. [192] prepared calcium alginate hydrogel coated on electrospun polyhydroxybutyrate/carbon nanotubes combining electrospinning technique and film casting as effective nanofiltration membrane. Carbon nanotubes were included into electrospun supporting layer for increasing the tensile strength (1 wt.% CNT). Composite membranes composed of a porous electrospun polyhydroxybutyrate/carbon nanofiber membrane as the support layer cross-linked by calcium ions to a hydrophilic thin film of calcium alginate hydrogel as the coating and barrier layer. Calcium alginate hydrogel film improved hydrophilicity of the surface as demonstrated by a decrease in WCA from 83.6° to 17.8°after coating. The flux and rejection rate of (CaAlg-c-PHB/CNT) nanofiltration membrane for dyes with molecular weight higher than 600 g/mol were 150.72 L/m^2^/h and 90%, respectively.

#### 4.6.4. Reverse Osmosis

As population growth increases, the global communities are facing water scarcity and increased demand for safe potable water. Desalination is the process of obtaining pure and drinkable water by separating inorganics and minerals from seawater or brackish groundwater. Since the first desalination plant in 1903 [193], many countries put more effort into developing desalination technologies and trends as alternative options to guarantee water supply sources, especially in countries with limited natural water resources. Regarding the energy and environmental concerns thermal desalination methods (evaporation of saline water followed by condensation of pure water vapor) relies on fossil fuels or electricity and suffers from higher energy consumption compared to membrane desalination processes. Membrane desalination technologies are drawing interest despite the economic cost and brine disposal concerns of desalination plants [194]. Reverse osmosis (RO) is the generally accepted procedure in modern desalination plants [195].

Reverse osmosis technologies are driven by gradients of pressure overcoming the normal osmotic pressure in salty water. Reverse osmosis membranes have the narrowest pore size, lowest permeability, and highest applying pressure among other pressure driven membranes (pore size of 0.001–0.0001 micron, 10–150 bar) [179]. The applying pressure drives the feed water to flow in the opposite direction of natural osmosis in organisms via a solution-diffusion mechanism. RO membranes remove a wide range of solutes such as all microorganisms, almost all the minerals including the nutrient ones (calcium, magnesium, and fluoride). There are significant considerations regarding the corrosivity of the water treated by reverse osmosis and adverse impacts of long-term drinking of poor-mineral desalinated water including dietary deficiency, raised osteoporosis risks, and metabolic syndrome (WHO, 2005a). Therefore, high-quality RO plants, often require post treatments units for adding some minerals to the demineralized water to meet the standards of safe drinking water and even agricultural irrigation standards [196]. Remineralization often carries out by blending with surface or ground water or calcium carbonate (calcite) bed filtration [197].

An example of using nanofibrous membranes for reverse osmosis desalination is a composite membrane composed of a 3-triethoxysilylpropylamine-functionalized cellulose acetate as the substrate and an electrospun tetraethyl orthosilicate-crosslinked poly (vinyl alcohol) (PVA) nanofibrous active layer. The modified active fibrous layer was prepared by blending zinc oxide (ZnO) nanoparticles and sodium alginate in polymeric electrospinning solution (0.1 wt.% and 0.01 wt.%, ZnO nanoparticles and NaAlg (sodium alginate), respectively)) in order to improve the antimicrobial property and permeability of the membrane. Dead-end reverse osmosis filtration showed an improved permeation flux and salt rejection. The enhanced ion exclusion is ascribed to negatively charged carboxyl groups of NaAlg on the surface [198].

In another work, a modified thin-film nanofibrous composite (TFNC) reverse osmosis membrane was designed for desalination by Wang’s group [199]. They used an electrospun nanofibrous scaffold for supporting the interfacial polymerized barrier layer. They indicated a relationship between pore size and TFNC performance. Higher defects in produced membranes accompanied with larger pore size in electrospun nanofibrous scaffold. Synthesized TFNC membranes composed of a non-woven poly (ethylene terephthalate) (PET) mat as the support, electrospun polyacrylonitrile (PAN) nanofibrous scaffold as the mid-layer and an ultra-fine cellulose nanofiber layer (CNs) as the top mechanically stable barrier. Hydrophilicity, and consequently permeation flux for RO, was improved by inclusion of piperazine or ionic liquid 1-octyl-3-methylimidazolium chloride using interfacial polymerization (96.5% rejection against NaCl, flux rate of 28.6 L/m^2^/h at 0.7 MPa).

#### 4.6.5. Forward Osmosis

**Figure 19:**
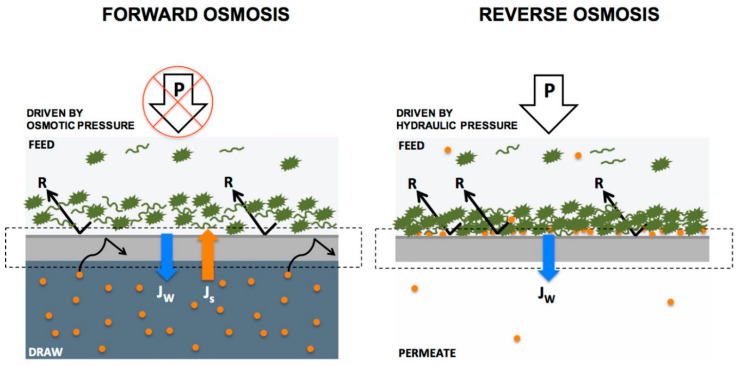
Schematic representation of differences in reverse and forward osmosis. Reprinted from [202], copyright FORWARDOSMOSISTECH.

Contrary to reverse osmosis principal, forward osmosis is driven by the osmotic pressure differences between feed and draw solution. Water diffuses spontaneously through a semipermeable membrane from the diluted solution (or feed side with low osmotic pressure) to concentrated solution (called the draw side with high osmotic pressure) [200]. A suitable draw solution is often selected for generating sufficient osmotic pressure for the process (solution of mono- or divalent salts, seawater, hydrogels, etc.) [201]. Finally, the concentrated draw solution gets diluted by the water permeate and this diluted draw solution is the product of forward osmosis. Differences between reverse and forward osmosis processes is depicted in Figure 19.

Diffusion of draw solutes in the opposite direction of water stream is the cause of enhanced internal concentration polarization (ICP) inside the membranes or porous support layer in TFC membranes which hinders forward migration of water in the draw side (retarded forward diffusion phenomenon). Hence, the thickness of forward osmosis membranes should be well designed for minimizing ICP [175] that adversely affects the performance. The active layer of TFC membranes may be faced either to the feed stream (ALFS (active layer facing the feed stream) mode) or the opposite configuration with the active layer faced to draw stream (ALDS (active layer facing the draw solution) mode). ALFS modes have commonly used in forward osmosis process in terms of ICP and fouling removal [203].

Forward osmosis is a well-stablished method in pre-treatment for reverse osmosis, wastewater treatment, decontamination, and food processing and the potential application of this membrane-based technology in treatment of medical radioactive liquid waste [204], hemodialysis [205], removal of heavy metal ions from water [206] have been studied. Recent advances on developing high performance TFC forward osmosis (FO) membranes using porous functionalized electrospun nanofibers have increased as a platform for modification of support layer [207]. Pana’s group synthesized a flat-sheet TFC membrane [208]. Electrospun PAN nanofibers were fabricated as a hydrophilic support followed by paper lamination for increasing the mechanical strength (WCA of 32.3 ± 1.3°). Interfacial polymerization was utilized for placing an active polyamide (PA) layer onto the nanofibrous support. FO process-accompanied with membrane distillation was used for treatment of antibiotic wastewater. Rejection ratio of 99.8% for tetracycline, lower ICP and an improved water flux compared to commercial FO membrane were achieved.

In another study aiming at improving support layer of TFC membranes, a polyacrylonitrile (PAN) nanofiber sheet was prepared by electrospinning then an interfacial polymerized polyamide selective layer was deposited on the PAN support layer. The resulting thin film composite polyamide membrane showed improved performance in a forward osmosis ALFS mode, compared to commercial polyethersulfone (PES) nanofiber membranes (water flux of 16 L/m^−2^/h^−1^ and reverse salt flux of 4 g/m^−2^/h^−1^) [209].

Electrospun nanofiber-supported TFC membrane was introduced by Gonzales et al. [175]. The electrospun polyvinylidene fluoride-polyacrylic acid (PVDF-PAA) nanofibrous support prepared via electrospinning followed by a post-heat treatment, for improving mechanical features. Hydrophilicity was induced by incorporation of polyacrylic acid (PAA) into the support (WCA of 74.82° in PVDF-PAA nanofibers compared to 136.38° in a pristine PVDF nanofibers). Modification of the TFC support was carried out by layer-by-layer polyelectrolyte deposition followed by interfacial polymerization on electrospun nanofibrous sheet. A polyelectrolyte layer deposited on the nanofiber surface acts as the selective polyamide layer of TFC membranes. This TFC membrane showed high performance as a FO membrane in terms of water permeability (4.12 L/m^−2^/h^−1^/bar^−1^, compared to commercial FO membrane) and mechanical strength as well.

#### 4.6.6. Membrane Distillation

**Figure 20:**
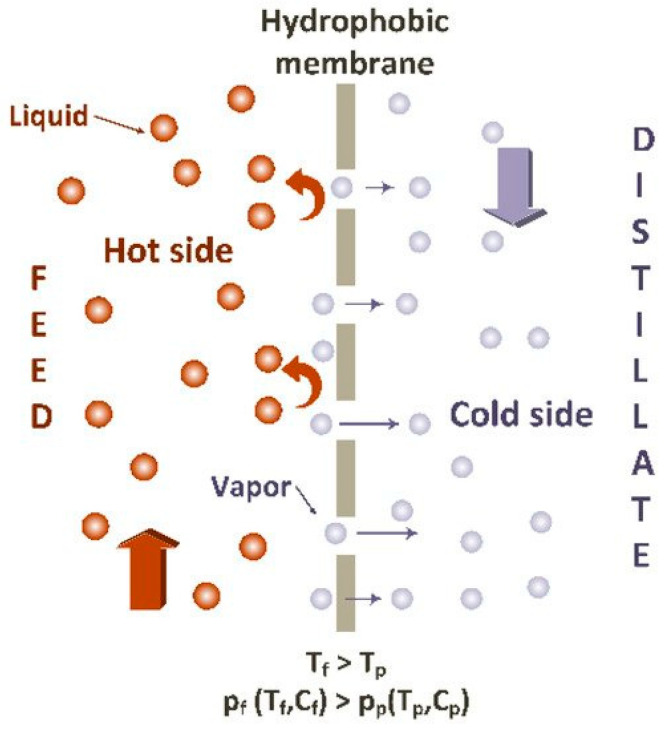
General illustration of a membrane distillation (direct contact mode). Reprinted from [210], with open access license.

Membrane distillation (MD) is a breakthrough technology mimicking the natural water cycle in nature. It is a membrane-based process in which a physical phase change derived by thermal treatment of the feed stream, giving rise to a vapor pressure gradient between two sides of a hydrophobic porous membrane. The selective rejection of liquid phase allows vapor diffuses through the membrane pores from the hot side (the warm feed with higher vapor pressure) to the colder side with lower vapor pressure. The vapor phase condenses in the cold distillate side or on the surface of a cold plate (air-gap membrane distillation) (Figure 20).

Membrane distillation has been considered as a promising alternative technology in desalination of seawater, treatment of concentrated hypersaline brines [211], wastewater treatments, separation of nonvolatile compounds from fluids and concentration of solutions such as urea recovery from urea synthesis plants [212,213]. Membrane distillation is a cost-effective procedure compared to other pressure-driven membrane technologies in view of working at low pressures and low temperature ranges that can be harnessed from solar energy, geothermal resources, or industrial waste heat. Hydrophobic membranes are less susceptible to fouling in this process. However, this technology has not yet been implemented as an established method for large scale industrial techniques. Recently, pilot studies have been investigated economical estimation of air gap and water gap MD for seawater desalination [214].

**Figure 21:**
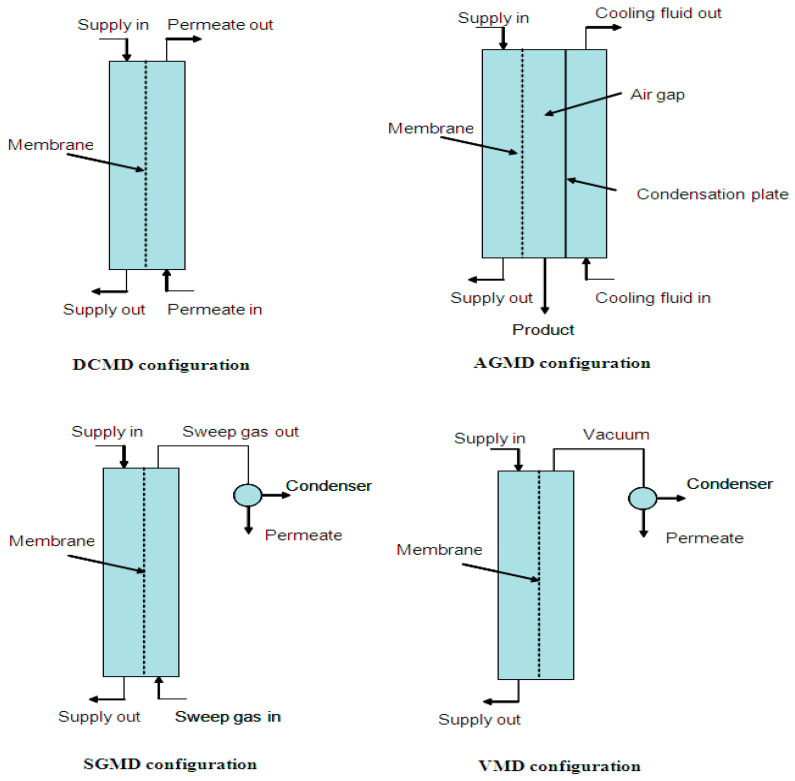
Different types of membrane distillation. Reprinted from [215].

Regarding the configuration of distillate side, different modes of membrane distillation processes have been developed (Figure 21) [215]. In direct contact membrane distillation (DCMD), the permeate gas is condensed at the distillate channel in a direct contact with the membrane. A vacuum pump or an inert gas is employed for carrying the permeate gas to an external condenser in vacuum membrane distillation (VMD) and sweeping gas membrane distillation (SGMD). The permeate vapor condenses on a cold surface separated by an air vent in air-gap membrane distillation (AGMD). The hydrophobic feature of distillation membrane has shown to be a key issue for rejecting liquid and reducing the membrane wettability. Nevertheless, dual-layered electrospun nanofibrous membranes composed of hydrophobic and the hydrophilic polymers have been proposed to be advantageous over hydrophobic single-layered membranes in terms of performance and mechanical strength [216,217,218,219]. In this regard, a dual-layered electrospun nanofibrous membranes composed of the hydrophobic polyvinylidene fluoride (PVDF) layer and the hydrophilic polysulfone (PSF) as the top layer was prepared by Khayet et al. [218]. The efficiency of the membranes was tested in desalination experiments by direct contact membrane distillation (DCMD). This membrane exhibited an increase in the permeate flux compared to single-layered electrospun nanofibrous equivalents that is attributed to the less packed structure of the prepared nanofibers. It was claimed that the permeate liquid penetrated inside the hydrophilic polymer layer reduces the path between the liquid/vapor interfaces and increases the DCMD performance. Additionally, lower electrical conductivity of the PSF polymer compared to PVDF is caused repulsion of the formed fibers during the deposition on the metallic collector and a loosely packed structure with higher void volume fraction is generated.

Very good performance in an AGMD, reported to be achieved by modifying the hydrophobicity and the surface structure of the membranes. Attia et al. synthesized composite nanofibers by embedding alumina nanoparticles in nanofibrous structures [220]. The hierarchical composite membrane consisted of electrospun nanofibers of PVDF as the support layer and a beaded layer on top was evaluated for treatment of contaminated water with AGMD. Improved AGMD performance in terms of higher permeate flux compared to commercial membrane (HVHP) was assigned to the enhanced membrane hydrophobicity and surface roughness. The surface beaded structure increases the air pocket area between the surface and water droplets on the membrane and consequently the liquid–vapor interfacial area increases which is the cause of superhydrophobicity (WCA of 154°) of the membrane and higher permeate efficiency in air gap membrane distillation process (AGMD).

Potential applicability of electrospun nanofibers as supporting material in dual layer membrane distillation was demonstrated by Deka et al. [221]. They showed that super-hydrophobicity can be enhanced by deposition of a hydrophobic aerogel on electrospun nanofibers using electrospraying. Polyvinylidene fluoride-co-hexafluoropropylene (PVDF-HFP) membrane coated aerogel/polydimethylsiloxane (PDMS)/polyvinylidene fluoride (PVDF) was prepared. Hydrophobic silica-based aerogel forms a rough texture on the base membrane which increases interfacial liquid-water vapor ratio that leads to flux improvement in DCMD (WCA of ~170° and a stable flux of 34.6 ± 1.9 L/m^−2^/h^−1^ for 7 days DCMD duration).

#### 4.6.7. Membrane Bioreactor

Biological membrane bioreactors are alternative to challenging conventional activated sludge processes. Integration of membrane-based technologies in conventional anaerobic biological treatments has been caused replacing large clarifiers (settling basins) and therefore reducing the cost. Moreover, high quality effluents and lower quantity of sludge are superior features of this method. Membrane bioreactor (MBR) technology in water reclamation and recycling is promising for saving large quantity of water from industrial and municipal wastewater that can be used for gardening, car washing, and cooling processes. Potential applications of this technology have also been evaluated for removing compounds of emerging concerns [222,223] and nutrient recovery as a substitute for nitrification/denitrification process [224,225].

**Figure 22:**
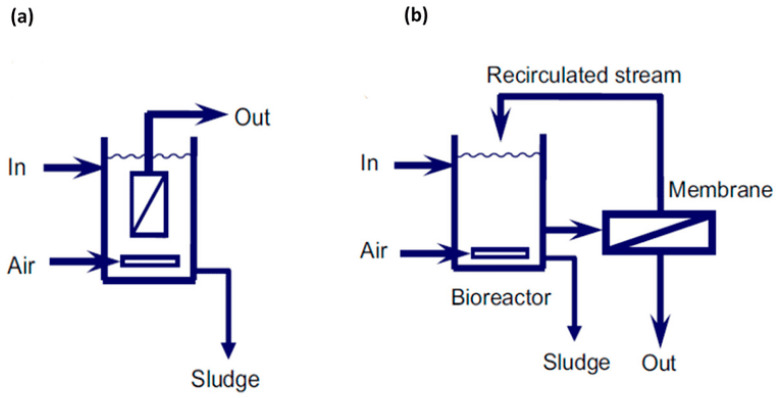
System configurations of MBRs: (**a**) immersed MBR and (**b**) side-stream MBR. Reprinted from [223] under open access license.

Membrane bioreactor technology relies on biodegradation of a mixed liquid (a mixture of raw or settled wastewater and activated sludge [226]) employing a microbial community. The process is consisted of a bioreactor tank where wastewater degraded by microorganisms under aerobic or anaerobic condition. A micro/ultrafiltration membrane module with different layouts is used for suspended solids removal. Regarding the aerobic or anaerobic process, an aeration or biogas diffuser assembled in bioreactor to mitigate membrane fouling. Considering the energy consumed for gas diffusion in bioreactors, developing strategies for fouling control have drawn significant attention. Mechanical cleaning using porous and nonporous scouring agents, non-adsorbing media such as polyethylene and polypropylene granules and activated carbon, have been applied in bioreactors as well [227]. Various membrane modules with multi-tubes, hollow-fibers, or flat-sheets have been used in MBRs. Process configuration of MBRs primarily included submerged or immersed MBRs and side-stream MBRs (Figure 22). In internal immersed MBRs, separation is carried out by an immersed membrane in the aeration tank and vacuum drives the flow of permeate inside the membrane. The wastewater is pumped from the aeration tank to an external membrane module in the external or side-stream configuration. New configurations for fouling control and cost management have been designed including biofilm membrane bioreactors (fixed or moving bed biofilms) [228]. Inclusion of particles or chemical carriers in biofilm membrane bioreactors (BF-MBR) reduces the transmembrane pressure (TMP) and fouling rate due to the attached biomass on biofilm carriers circulating in the aeration tank [229,230]. Higher effluent quality can be achieved using high retention membrane bioreactors (HRMBRs) where NF, FO, and MD membranes were combined with conventional MBRs because of the longer biodegradation time [223,231].

Electrospun membranes draw growing interest in water treatment bioreactors as well. Functionalized nanofibrous membrane with special wettability for irreversible fouling control, chemical, and physical strength of membrane have been used in bioreactors [232]. L.F. Ren et al., observed that superhydrophobic/superorganophilic membranes [233] can be utilized in external extractive membrane bioreactors (EMBR) for simultaneous organic permeation/inorganic rejection. External extractive membrane bioreactors have been introduced for treating industrial wastewater with extreme pH, high inorganic concentrations comprising toxic or recalcitrant organics which cannot be treated by conventional biological treatments because the growth and function of microorganisms is hampered by these harsh saline conditions [234,235]. In this configuration, a membrane module assembled in the bioreactor separates organic species to a biozone where they can be degraded in the absence of inorganics. Electrospun polydimethylsiloxane/polymethyl methacrylate (PDMS/PMMA) membrane with special wettability provided phenol permeation and salt rejection (phenol permeation efficiency (99.2–100.0%) for treating phenol-laden saline wastewater (WCA of 160.9° and phenol CA of 0°)).

Moradi et al. [236] reported that reversible and irreversible fouling is reduced by embedding fumarate-alumoxane (Fum-A) nanoparticles to electrospun PAN microfiltration membranes for MBR applications. Compared with pristine electrospun PAN membranes, anti-fouling efficiency and improved pure water flux (1840 L/m^2^/h) was related to the surface hydrophilicity due to the extra hydroxyl and carboxylate groups on the surface of Fum-A. Hydrophilic membranes form a water layer on their surface that acts as a barrier for foulants. Modification of tubular electrospun polyacrylonitrile nanofibers was carried out by heat treatment followed by coating a polyamide layer on electrospun backing layer [237]. The modified membranes showed improved water flux (500 L/m^2^/h) and salt rejecting performance compared to conventional osmotic membranes at external and submerged bioreactors under pressure. The low salt flux value of 0.5 g/m^2^/h may be a beneficial feature for suppressing salt accumulation in bioreactors. Furthermore, unusual results were obtained compared to osmotic membranes. Increasing the applied pressure on the feed side accompanied by improving the water flux with no salt accumulation in the feed side. The salt content of feed side is kept constant compared to osmotic membranes because of the increased flow of salt to the concentrate side with no reverse salt stream.

#### 4.6.8. Antimicrobial Treatment

**Figure 23:**
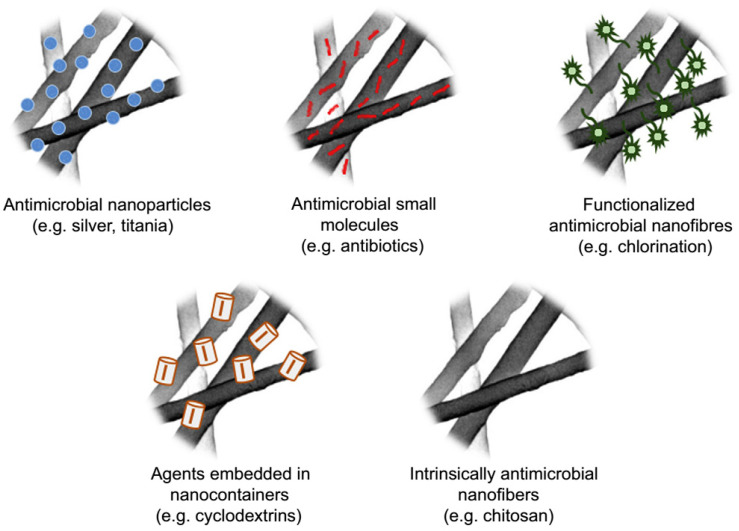
Different designs of antimicrobial nanofibers. Reprinted from [241]. Copyright (2021) with permission from Elsevier.

Nanofiltration and reverse osmosis are specific procedures for eliminating microorganisms from water [187]. Nevertheless, biofilm formation as a result of deposition and growth of bacteria and viruses onto the membrane reduces their permeability and filtration performance. The demand of highly efficient technologies for complete rejection of pathogenic pollutants from water have stimulated numerous studies on increasing anti-biofouling characteristics and improving water flux in membrane-based separations [238]. As reviewed by Botes et al., surface functionalization of electrospun nanofibers by nano-biocides provides nanofibers enabled targeting pathogenic microorganisms in a broad range of applications including air filtration, water and wastewater purifications and medical applications [239,240]. Manipulated electrospun nanofibers either as antimicrobial active layer or as the backing can be employed in membrane-based technologies. Antimicrobial properties can be intrinsically in the polymer or be achieved by blending or post treatment procedures such as plasma treatment and surface graft polymerization of nanofibers. Figure 23 illustrates different approaches of designing electrospun nanofibers for antimicrobial treatments. Nanofibers control the release of their biocidal components and improve the efficiency of antimicrobial remediation [241].

Biocidal agents including metal or metal oxide nanoparticles (silver, iron, copper, iron oxides, copper oxide, titanium dioxide, etc.), synthetic or bio-based disinfectants like N-halamines, quaternary ammonium compounds, and chitosan have been studied widely [177,241,242,243]. Single-, bi-, or multi-layered electrospun membranes for separation processes have developed. For instance, Kwon et al., reported an antimicrobial bilayer membrane for treating oily wastewater [244]. Silver nanoparticles embedded into the electrospun hydrophobic polyimide support layer presented antifouling tendency against bacteria and proteins. A hydrophilic PVA nanofibrous layer attached to the support layer as a barrier for preventing fouling by oily foulant. Ceramic nanofibrous membranes have also been designed against pathogenic waterborne viruses. As an example, nanofibers of polymer solutions containing acetates of iron (II) and copper (II) were fabricated by electrospinning. Calcination of fibers removed the organic contents and ceramic fibers of hematite and copper oxide (CuO) were prepared [242]. Hematite fibers showed superior adsorption over copper-based fibers in batch adsorption experiments. The better performance of hematite fibers is attributed to higher specific surface area for MS_2_ bacteriophage removal. In another article, a three-layered multifunctional portable device was suggested for water purification by Taheran et al. [245]. The first layer comprised an electrospun polyacrylonitrile-chitosan membrane (PAN-CTN, 85:15) with antimicrobial activity. Polyacrylonitrile-activated biochar-laccase composite membrane (PAN-BC-LAC) was used as the biocatalytic middle layer for eliminating micropollutants (99% efficiency) and finally an adsorptive membrane enabled reducing color and turbidity. In another study, modification of natural polysaccharide Sesbania gum (SG) was carried out through epoxidation, amination, and chlorination [246]. Nanofibers of chlorinated SG was then fabricated by electrospinning using polyacrylonitrile (PAN) as the support matrix. Nanofibers of chlorinated SG consisted of N-Cl covalent bonds resembling N-halamines showed antibacterial activity against *Escherichia coli* and *Staphylococcus aureus*.

### 4.7. Gas Sensor

**Figure 24:**
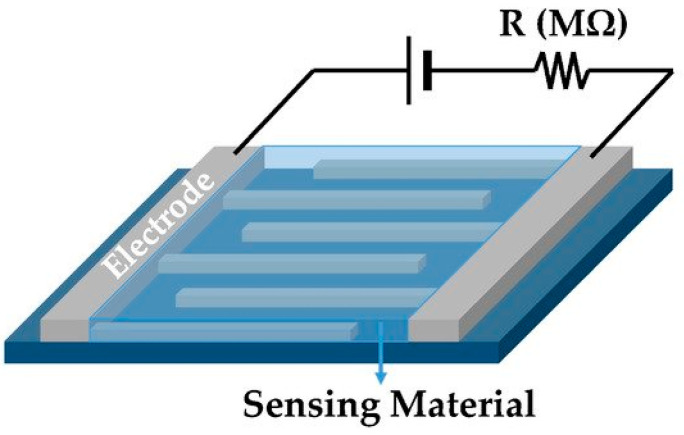
General representation of a chemiresistor sensor. Reprinted from [248], with open access license.

Gaseous pollutants and volatile organic compounds (VOCs) are two major contributors to air pollution. Gas sensors or gas detectors have been designed for sensing odorless gases or trace concentration of vapors which cannot be detected by human nose [247]. The most common fields of application are air quality monitoring, healthcare monitoring, and gas detection systems like smoke, humidity, or carbon monoxide sensors. Various sensing procedures have been developed by employing an active sensing material where the interaction of a target gas induces a change in physical (resistance, frequency, current, or voltage), chemical, optical, or thermal properties of the sensing component and then the respond is detected either optically or electrically by a transducer that converts induced changes to a measurable electric signal. For instance, a chemiresistive gas sensors measure the changes in electrical conductance/resistance of a sensing material when exposed to a target gas (Figure 24). Metal oxide-based gas sensors particularly SnO_2_, ZnO, and TiO_2_ and organic-based chemiresistive sensors are two main groups of this category. Mechanisms of sensing and resistive responses depends on the oxidizing or reducing nature of the target gas and charge carriers on the surface of semiconductors (electrons or holes) [247].

**Figure 25:**
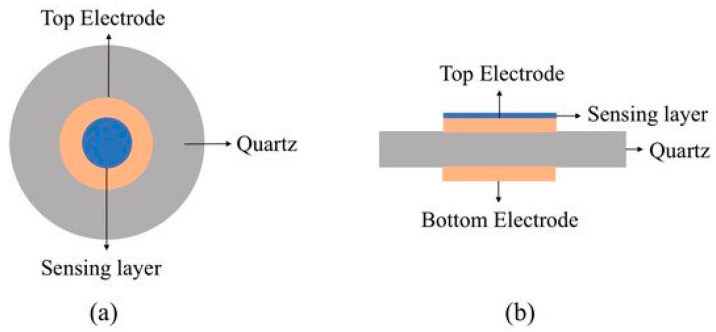
The (**a**) top and (**b**) side view of a quartz crystal micro-balance sensor. Reprinted from [248], with open access license.

Besides, other types of sensors have been developed. Optical gas sensors measure the changes of light property like wavelength in the sensing material. For example, IR point sensors measures the absorption and reflection of IR waves through the interaction of analyte gas. In calorimetric sensors heat is generated through the interaction of target gas with the sensing materials. Quartz crystal microbalances (QCM) as a major type of acoustic wave sensors has been widely applied for chemical sensing. Quartz crystal microbalance technology is principally based on the changes in resonance frequency of piezoelectric quartz crystals with the mass uptake on the sensing layer. A schematic view of these sensors is depicted in Figure 25.

**Figure 26:**
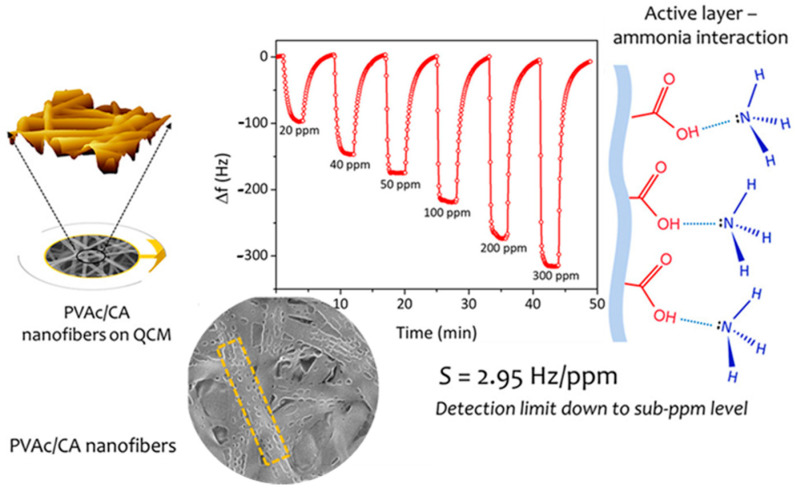
Electrospun PVAc/CA nanofibers as the active layer of quartz crystal microbalances ammonia sensing. Reprinted with permission from [251]. Copyright (2020), American Chemical Society.

Various kinds of materials including conductive polymers, metal-oxide semiconductors and composite materials have been applied as sensing materials. Gas sensors based on electrospun membranes have proven higher sensitivity and reduced the energy requirements of metal-oxide based sensors, thanks to their larger specific surface area, highly porous structures, and special electrochemical characteristics [249,250]. QCM sensors coated with electrospun nanofibers have revealed higher sensitivity and fast responses than solid film-coated counterparts. Roto et al. used electrospun polyvinyl acetate (PVAc) nanofibers doped with organic acids for functionalization of QCM electrodes as the active layer. Oxalic acid, tartaric acid, and citric acids were investigated as dopants. PVAc nanofibers doped with citric acid (PVAc/CA) showed more porous structures than the two other examined samples. High selectivity and sensitivity (2.95 Hz/ppm) with very low detection limit (LOD, 550 ppb) of PVAc/CA nanofibers for ammonia sensing was attributed to the increased acid–base interaction between carboxyl groups of citric acid and ammonia owing to the highly porous structure of the fibers (Figure 26) [251].

Al-Hazeem et al., fabricated an n-type semiconductor hydrogen detector based on electrospun TiO_2_/PVP nanofibers (NFs) (mean diameter of 41–281 nm) [252]. Sensing performance in terms reversibility and response time was investigated. The effective sensitivity of 63% at 1000 ppm for H_2_ gas with very low power consumption (60 mW) was attributed to highly porous structure and large surface to volume ratio of one-dimensional nanofibers of TiO_2_/PVP.

Doping electrospun nanofibrous membrane with pH sensitive dyes have been used for fabrication of colorimetric sensors [253] as well. Teli and Nadathur prepared a bromothymol blue doped silica and silica/copolymer membrane for detection of ammonia and HCl vapors [254]. Color change is the response of a pH-sensitive chromophore in bromothymol blue when exposed to acidic or basic environment. They demonstrated a correlation between porosity and hydrophobic/hydrophilic nature of the matrix on sensing response of the active dye component. Additionally, research interests on improving the selectivity of these materials to meet the requirement of practical sensing materials have grown significantly. Selective sensors capable of quantifying target gas selectively in the presence of other analytes have been developed [255]. Nanomaterial-incorporated nanofibers with high surface area, composite nanofibers, or embedding filters in chemical gas sensors for target gas sensing without interfering with similar compositions have been reported. Filters play an adsorptive role in retaining the interfering compounds [255]. As an example, Liu et al. successfully fabricated an acetone sensor for diagnosis of diabetes via exhaled breath. The active semiconductor shell of In_2_O_3_ nanowires with Pt core was synthesized by co-electrospinning method and coated with a silica mesoporous SBA15 (santa barbara amorphous-15) molecular sieve layer as the moisture barrier [256]. The sensor showed good selectivity, stability, and high sensing performance with a detection limit of 10 ppb for acetone in the presence of high humid condition of the exhaled breath.

### 4.8. Liquid Sensor

Electrospun nanofibrous-based sensors have also been entered in the field of detection of liquid analytes due to their excellent properties. Developing selective and sensitive sensors and biosensors with low detection limits and selective detection of a desired molecule among interfering molecules have great importance for disease detection (detection of special biomarkers such as glucose, peptides, proteins, and antibodies), immune-sensing (detection of serum or other body fluids), hydrogen peroxide, and toxin detection in food and cosmetic industry and for environmental monitoring applications [257]. It is needless to say that sensitivity of liquid sensors linked to the amount of species interacting with the active sensing materials. Electrospun nanofibrous-based transducers with high surface area and interconnected structures for facile diffusion and mass transfer of biochemical or chemical species have demonstrated prospective application in improving sensing function. Recently, in separate reviews, Al-Dhahebi et al. and Krishnan et al. discussed the efficacy of modified electrospun nanofibers by incorporating graphene-based nanomaterials and their application in electrochemical biosensors and sensors [258,259]. Cabral et al. reported luminescent graphene quantum dots incorporated electrospun poly (vinyl alcohol) nanofibers as suitable substrates for enzyme immobilization [260]. Glucose oxidase enzyme immobilized nanofibers exhibited efficient glucose detection in solution (range of detection: 1 mM to 10 mM; detection limit of 12 µM) and were proposed as biocompatible sensing materials in optical sensors capable of detecting blood glucose levels. Guan et al. reported a non-enzymatic sensor mimicking redox catalytic function of horseradish peroxidase (HRP) for selective reduction of hydrogen peroxide in the presence of interfering compounds [261]. The role of structure and conductivity of the composite material on improving the performance of sensors was investigated. They synthesized a liquid sensor to detect hydrogen peroxide based on porous N-doped carbon nanofibers embedded with PtNi alloy nanoparticles and CeO_2_ nanoplates via electrospinning combined with post heat treatments of polyvinyl pyrrolidone incorporated metal salts (H_2_PtCl_6_·6H_2_O, NiCl_2_·6H_2_O, Ce(NO_3_)_3_·6H_2_O). PtNi alloy/CeO_2_ plates/N-doped carbon nanofibers showed stable structure and superior sensing functions (high sensitivity of 345.0 µA/mM^−1^/cm^−2^) for hydrogen peroxide detection in cosmetic products (range of detection: 0.5 µM–15 mM; detection limit of 0.025 µM). Electrocatalytic activity of the fibers was attributed to highly porous structure, high surface area of the fibers and the small size and uniform distribution of PtNi alloy nanoparticles and CeO_2_ plates because of the facilitated electron transfer.

### 4.9. Air Filtration

**Figure 27:**
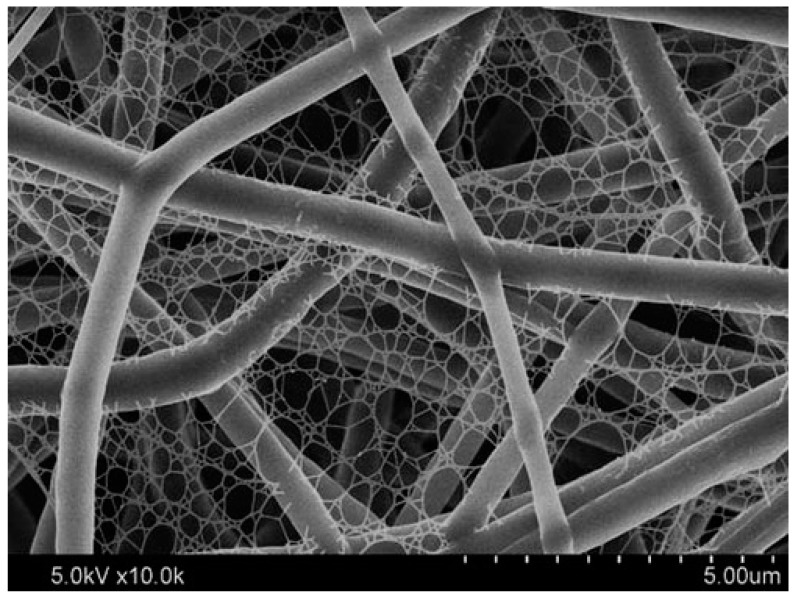
Representative view of nanofiber/net membrane. Reprinted with permission from [268]. Copyrights (2011), John Wiley and Sons.

Both short-term and long-term exposures to airborne particulate matter (PM) pollution contributes to respiratory and cardiovascular disease and cancers (WHO air quality guidelines 2005). PM10 (with an aerodynamic diameter smaller than 10 µm) and PM2.5 (fine particles with an aerodynamic diameter smaller than 2.5 µm) are two major components of particulate matter that pose health risks. Electrospun nanofibrous filters with nanosized fiber diameters, highly porous structures, large pore size, tortuous channels, along with their light weight are good candidates for PM filtration compared to conventional micro-sized fiber-based filters. However, their low mechanical strengths limit their application independently and therefore, they supported on nonwoven substrates or used as composite materials. Pure polymeric filters and composite polymer-based filters have been studied. Composite polymer-based filters promote the mechanical and tensile strength of the membrane [143]. Electrospinning is a fascinating method which allows the regulation of hydrophilicity and hydrophobicity of the filters, fine tuning of the porous structure and fiber diameters, endows enhanced air transfer and PM rejection of filtration media for various application including air cleaners, protective masks, appliances, and medical equipment [262,263]. The urgent need for surgical and respirator masks during the global concern of COVID-19 pandemic in 2020 have prompted numerous studies on investigating the superior features of electrospun nanofibrous mats in protective face masks [264,265,266]. Innovative nanofiber net structures inspired by the nature (spider webs) with superior features such as ultralight weights and ultrathin thicknesses have improved breathability without compromising the filtering performance (Figure 27) [263]. Electrospinning/netting method for the preparation of nanofiber net filters is an electro-netting process combined with liquid jet formation in conventional electrospinning techniques where a net-like structure forms by splitting of small, charged droplets via phase separation, ionic salts initiated splitting, hydrogen bond mechanisms, and intertwining between branching jets [267].

As an example, superlight weight air filters using superhydrophobic nanofiber net structures with sieving and surface adhesion property was reported by Liu et al., to filter particles under 0.3 microns (PM0.3) [269]. Polyvinylidene fluoride nanofiber/net membranes fabricated via in situ electret electrospinning/netting method. A DC voltage was applied to generate polarization charges in Taylor cone and given rise to surface-charged 2D nanonets. The surface potentials of nanonets increased due to the PVDF crystal phase transition from nonpolar α-phase to polar β-phase in nanonets. By virtue of nanoscale dimeter (≈21 nm), small pore size (≈0.26 µm) coupled with high surface potential of 6.8 kV, polyvinylidene fluoride nanofiber/net membranes presented 99.998% efficiency for capturing small NaCl PM0.3 aerosol.

Another study conducted by the same authors reported the fabrication of surface-modified nanofiber net filters inspired by the nature [270]. Wet-adhesive nanofiber-net filters were prepared inspired by polymeric structure of Byssus in marine mussels (a creature capable of attaching to wet rocks). Polyacrylonitrile/dopamine hydrochloride nanofiber net membranes (PDA/DA) were prepared by electrospinning/netting on nonwoven fabrics followed by modification of the surface through self-polymerization of DA. A thin adhesive polydopamine (PDA) film (containing catechol and amine groups like the functional groups of Mussel Byssus) was formed on the surface of nanofibers. Highly porous nano-architecture networks (porosity, >92%), with small diameters (~27 nm), small pore size (~0.28 μm), and excellent breathability (low air resistance, 108 Pa) demonstrated high filtration efficiency of 99.99% for PM0.3. Nanofiber net filters exhibited good stability under extreme high humidity (>99.97% removal efficiency for PM0.3) due to sieving and wet adhesive features. Improved adhesion effect between pollutants and the nanofiber net filter was ascribed to the highly polar poly dopamine thin film on the surface (dipole moment of 3.5 D) giving rise to stronger dipole–dipole and induced dipole interactions between pollutants and the surface.

Organic/inorganic nanoparticle incorporated polymer-based filters improves their adsorptive capacities as well. Maddah et al. fabricated a series of electrospun polyurethane nanofibers based on composite carbon nanotubes incorporated Ni-Zn ferrite particles (PU-CNT, PU-CNT-Fe_3_O_4_, PU-CNT-NiFe_2_O_4_, PU-CNT-Ni_0.5_, Zn_0.5_Fe_2_O_4_) [271]. The capacity of these materials was assessed as nanofibrous composite media for H_2_S filtration. The superior performance of PU-CNT-Fe_3_O_4_ nanofibers in H_2_S removal (498 mgH_2_S/g) compared to pure PU nanofiber and other PU nanofibers containing CNT-ferrite composites was ascribed to sulfurization (sulfide formation from the reaction of iron ions with hydrogen sulfide).

Biocidal activity was induced into polyacrylonitrile nanofibers by incorporating silver nanoparticles [272]. Nanofibers deposited on a nonwoven micro-fibrous substrate by electrospinning. Filters with different silver nanoparticle contents were studied. Ag/PAN nanofibers displayed antibacterial efficiency against *E. coli* bacteria. Highly porous nanofibers with 1% and 10% AgNPs exhibited excellent filtration efficiency (≈100%) and good quality factor (0.04 Pa^−1^) for removing sodium chloride (NaCl) aerosol particles (9–300 nm diameters). Filters with 50% AgNPs showed the lowest pressure drop and high filtration efficiency (>98.6%) in elimination of nanoparticles (9–300 nm) from the air (E11 according to standards of EN 1822). The lowest pressure drops, high permeability and larger void space in nanofibers with 50% AgNPs content were related to the higher concentration of silver ions in the electrospinning solution that increases the viscosity of the solution, generating a lower rate of nanofiber deposition on the collector.

### 4.10. Catalyst Supports

Supported catalysts have demonstrated interesting benefits including facile recovery and reusability of the heterogenized catalyst either magnetically or gravitationally and preventing leakage of the catalyst especially in the case of precious metal catalysts. Application of electrospun nanofibers directly as catalyst or catalyst supporting materials are one of the most studied research areas [273]. Encapsulation of catalyst or catalyst precursor in polymeric matrix of nanofibers and coating of nanofibers with catalytically active components improve the physicochemical stability of the catalyst. Additionally, nanofibrous-based catalysts with their highly porous architectures and large surface area to volume ratio further extend specific surface area of the catalyst and accessibility of active sites of catalyst to the desired reactant, consequently increasing the reactivity of the catalyst at low catalyst loadings. Nanofibers incorporated enzymes, metal or metal-oxide nanoparticles, metallic complexes [274] and metal-organic frameworks (MOFs) [275], directly or through post-treatments procedures have been reported [273]. Chemical or physical attachments of catalytically active functional groups on the surface, self-polymerization, and calcination have been used for surface functionalization of nanofibers. Some of the recently reported works on using electrospun nanofibers as catalyst supports summarized in Table 1.

### 4.11. EMI Shielding

Electromagnetic radiation or interference (EMI) emitted from surrounding electronics is another concerning pollution along with water and air pollution in recent years. The great importance of protecting sensitive electronic equipment against electromagnetic interference in practical applications including portable devices, critical defense systems, and aerospace has provoked attempts for developing flexible high-performance EMI shielding materials with light weight and wide absorption bandwidth while maintaining mechanical strength. EMI shielding materials capable of absorption or reflecting electromagnetic interference playing a shielding role against electromagnetic waves. Conductive materials such as metals, conductive polymers, graphene [281], and composites coated on sheets and fabrics or in the form of foams and gaskets have been developed. Variety of fillers have been embedded into composite polymer-based materials for improving their electrical conductivity (e.g., metal nanoparticles, graphene, carbon nano/microfibers) [282,283,284]. Total effectiveness of absorption and reflection loss mechanism in EMI shields is affected by material characteristics including type of filler, electrical conductivity, size, shape, thickness, and morphology of the shields [285]. Multiple reflection loss of the electromagnetic wave is the major mechanism in porous materials with large surface area due to their highly inter-twined structures. Electrospun conductive polymer-based membranes provide with higher corrosion resistance and flexibility compared to poorly flexible metal-based materials with corrosive features. For example, metal nanoparticles (AgNP, CuNP, NiNP) were distributed on the surface of crosslinked electrospun PAN nanofibers via an electroless deposition procedure [286]. Thin PAN nanofibers coated silver nanoparticles (thickness of ~53 µm) exhibited a multilevel shielding behavior with the highest electrical conductivity, superior EMI shielding effectiveness (~90 dB), good mechanical strength and excellent flexibility compared to pure metal alternatives. The high shielding effectiveness of the hybrid membrane was ascribed to the conductive metal nanoparticles distributed on the surface and to the porous structure of the material providing higher interfacial interaction for electromagnetic waves to reflect and scatter inside the shielding material. It should be noted that metal additives with high thermal conductivity increase the thermal conductivity of the polymers as well and facilitate the thermal energy dissipation generated from absorbed energy of EM waves. For instance, Kim et al. aimed at improving thermal conductivity of electrospun nylon 66 NFs [287]. Multi-layer composite films of silver-nylon 66 nanofiber prepared by stacking Ag thin films deposited on nylon 66 nanofiber layers followed by hot pressing and condensing them into a single electrospun mat. Composite mats showed EMI shielding effectiveness of 60.6 dB, specific shielding effectiveness (SE) of 67.9 dB cm^3^/g and a high thermal conductivity (4.17 Wm^−1^/K^−1^ at room temperature). Multiple inter-layer reflections in multi-layer structure of the hot-pressed electrospun mats caused absorption of EMI wave to be the major energy loss mechanism, compared to bulk material. The authors attributed higher thermal conductivity of the hot-pressed electrospun mats to the more aligned arrangement of nylon 66 chains after electrospinning and increased crystallinity of the polymer. Huang et al. prepared a flexible composite film comprising electrospun TiO_2_/SiO_2_ (TS) nanofibers as the substrate [288]. Polypyrrole films were then deposited on the surface of TS fabrics and formed a double-continuous conductive network containing core of TiO_2_/SiO_2_ nanofiber and polypyrrole shell. Thin reduced graphene oxide sheets were further attached on the surface of the nanofibers. The fabricated films exhibited EMI shielding effectiveness of ~30 dB in X band region (8–12 GHz) and a higher specific EMI shielding effectiveness (SE) of ~13829 dB·cm^2^/g^−1^ compared to most of metallic EMI materials.

### 4.12. Carbon Dioxide Capture

Carbon dioxide with the ability to trap heat is the leading contributor to global warming and ocean acidification and mainly entered to the atmosphere through fossil fuel combustion according to IEA (International Energy Agency) [289]. Regarding the need to minimize overall CO_2_ emissions to the atmosphere, carbon capture and storage (CCS) technologies have been developed and referred to capture of emitted carbon dioxide from industrial processes and then releasing the captured CO_2_ and transportation to a secure storage. A vast number of chemical and physical based systems [290] have been used for large-scale carbon dioxide capture such as adsorption-based strategies; liquid amine absorption or gas sweetening; cryogenics CO_2_ liquefaction [291]; high pressure membrane filtration; pre/post or oxy-fuel combustion capture methods involving capture of generated carbon dioxide before or after the combustion process. Solid or liquid materials including polymer-based filters, ionic liquids (ILs), ionic polymers, porous crystalline metal-organic frameworks (MOFs), amine sorbents, and various forms of carbon have been proposed and evaluated as capturing materials, amongst them adsorption-based technologies employing solid porous substrates have demonstrated greater potential efficiency in industrial application because of the simple operational implementation and the high volume of gas captured per volume of solid material that reduces the total cost of procedure [292].

Designing and developing CO_2_ capture technologies and industrially practical CO_2_ separation materials for highly selective capture of CO_2_ from a mixed gas stream having good CO_2_-capturing capacity and stable adsorption/desorption performance has spawned significant effort around the world. Electrospun nanofibrous materials with their unique structures—including high surface area to volume, ease of functionalization, high flexibility, tailorable pore size, and surface chemistry—have motivated researchers to explore and assess the potential application and performance of such materials and their modified forms as CO_2_ capturing agents. Surface functionalization of the adsorbents endows active functional groups with intrinsic affinity to CO_2_ and provides more active sites for selective CO_2_ adsorption as well. Amine-bearing nanofibers have displayed high CO_2_-capturing capacity due to the high affinity of NH_2_ group toward CO_2_ molecules that provides further CO_2_ adsorbing sites and enhances selectivity of the material. For instance, Abbasi et al. reported grafting poly glycidyl methacrylate onto electrospun syndiotactic polypropylene nanofibers via radiation-induced grafting [293]. Nanofibers grafted with poly glycidyl methacrylate were further functionalized with different types of amines. They investigated effects of different parameters on the degree of amination. Amine immobilized-nanofibers showed high stability after four subsequent adsorption/desorption runs. Additionally, primary, secondary, and tertiary aminated nanofibers exhibited CO_2_ adsorption capacities of 2.87, 2.06, and 0.94 mmol/g respectively.

Various methods have been used for increasing specific surface area and porosity of the fibers. As an example, Heo et al. fabricated a porous carbonaceous material by carbonization of PAN/PVDF hybrid composites [294]. Carbonized PAN/PVDF nanofibers were physically activated by steam (5 mL/h, 60 min) and displayed increased specific surface area and a higher total pore volume after steam activation (925 m^2^/g and 0.404 cm^3^/g). Compared with non-activated composite, CO_2_ capture capacity was significantly improved (2.21 mmol/g, 1 bar; under flue gas condition (15% CO_2_ in N_2_). In a similar study, activated carbon nanofibers by carbonization and activation of reduced graphene oxide incorporated-PAN nanofibers were synthesized by Othman et al. [295]. Meso- and micropore volumes and specific surface area of the fibers were significantly increased by activation compared with non-activated composite materials. The effect of different loadings of reduced graphene oxide in nanofibers were investigated. Composite with 10% reduced graphene oxide showed high specific surface area and great CO_2_ capture capacity of 58 mmol/g at 15 bar and 25 °C. Electrospun materials containing MOF nanoparticles have developed as well and demonstrated mechanical properties along with improved CO_2_ adsorption capacities. Organized and open porous structure of MOFs coupled with high surface area of electrospun nanomaterials supply more active sites enables them to improve capturing performance. Choi et al. reported fabrication of polyacrylonitrile nanofiber immobilized with different loading of ZIF-8 MOF and HKUST-1 MOF particles through electrospinning [296]. Improved CO_2_ adsorption capacities for MOF-loaded nanofibers assigned to increased pore volume due to the additional porous structure of MOFs. Further functionalization of MOF-loaded nanofibers by impregnation of polyethyleneimine remarkably improved CO_2_ capture capacity and CO_2_/N_2_ selectivity of PAN ZIF-8 mats. Besides, CO_2_ adsorption capacities were decreased by increasing the polyethyleneimine content because of the decreased pore volume due to the pore blocking by polyethyleneimine molecules.

### 4.13. Ion Exchange

Ion exchange is a simple strategy for selective removal of cationic or anionic species depending on the charge of ionic active sites. Ion exchange nanofibrous membranes have revealed excellent features to be good candidates for removal of dye and dissolved poisonous heavy metal ions compared to precipitation, chelation, and adsorption-based technologies. For instance, zeolite [297] nanoparticles encapsulated in nanofibers improved their recovery and reusability.

Nanofiber-based ion exchange membranes have been studied in delivery systems, water treatments, medical applications, and catalysis. These materials have also been used in energy-related areas. Electrospinning of materials with intrinsic ion exchange ability or functionalized by grafting or encapsulation of ion exchange agents into polymeric matrix have indicated great potential of application in batteries, fuel cells, electrodialysis and reverse electrodialysis [298]. Low tortuous structures, interrelated pores, and high surface area of the fibers render higher ion exchange capacities and ion conductivity in energy conversion devices. It was found that cation and anion exchange nanofiber membranes [299] have displayed thinner thicknesses and better electrochemical and mechanical features in polymer electrolyte fuel cells than membranes without nanofibers [300]. Migration of ions between two electrodes is facilitated by highly aligned orientation of ionic sites on the ion conductive membranes [299]. For example, electrospun polystyrene membranes modified by post-sulfonation reaction reported by Jalal et al. for fuel cells displayed improved ion-exchange capacity (2.857 mmol/g) and proton conductivities (8.8 × 10^−4^ S/cm) depending on the sulfonation time [301].

Electrospun micro- and nanofibers of polystyrene loaded with metal-layered double hydroxide particles showed higher stability, provided by thermal stability of the metal-layered double hydroxide, coupled with chemical stability from embedding layered double hydroxides into PS polymer matrix [302]. Removal of Cd^2+^ ions were proposed with the adsorption of Cd^2+^ ions by replacing Al^3+^ and Mg^2+^ ions in layered double hydroxide component of the composite membrane. In another study, Johns et al. prepared functionalized polyacrylonitrile nanofibers deposited on a layer of PVDF nanofibers [303]. Surface functionalization were carried out by electrospinning of PAN precursor solution incorporated with different N- and P-containing binding agents or through post-functionalization procedures. Functionalized nanofibers were studied for removal of uranium (U) ions from simulated contaminated acid mine drainage (pH 2) and contaminated water sources (pH 6.8). Quaternary ammonium groups of Aliquat^®^ 336 on nanofibers appeared to have good U uptake at nearly neutral pH. PAN nanofibers bearing amidoxime group (–C(NH_2_) = N–OH) exhibited the highest uptake (at pH 6.8).

## 5. Conclusions

Electrospinning is the most successful techniques to fabricate nanofibrous materials with a great control of final outcome. Together with their intrinsic properties like high surface-to-volume ratio, micro/nano-sized fibers, process simplicity and process scalability, electrospinning can provide competitive advantages. As presented in this review, electrospinning has provided significant results in both DSSCs and perovskite solar cell applications, while it has shown its potential in fuel cell fabrication with electrospun nanofiber-based electrode and electrolyte membranes. In addition, larger surface area of nanofiber materials has become crucial in energy storage facilities, since rechargeable batteries and supercapacitors have utilized nanofiber membranes constantly. In this review, a number of electrospinning applications have been discussed with details on their materials and specially the design. On the other hand, controllable physical and chemical properties of nanofiber membranes have attracted environmental remediation field as well. Water treatment, filtration, contaminant removal, and related treatment approaches were reviewed thoroughly while focusing on the applications of nanofibers and various related designs. Also, applications of air filtration and environmental monitoring were considered and summarized.

There is no doubt that electrospinning technique has contributed the advancement of energy conversion, energy storage, and environmental applications throughout past decades. Its capability of fabricating nanofiber in composite with multiple materials has become one of key properties in recent years, it can fuel future directions as well. With the introduction of novel and innovative materials in the energy field, electrospinning provides unique material properties with cost-effective and scalable fabrication. With growing concerns about the environment, water treatment, and water and air filtration will become a key requirement in the future. Significantly, the recent COVID-19 outbreak has proved the necessity of having competitive advantage over dangerous pathogens, in all possible ways.

In the future, it will be crucial to keep working on the safety and well-being of the humans and other living species. Recyclability of nanofiber membranes should become a key concern in all application fields, together with the performance enhancement it can provide. Use of recycled materials will also be an interesting topic of discussion in the future, since it can be highly beneficial.

## Figures and Tables

**Table 1:** Some of the recently published studies relating heterogenization of catalysts on electrospun nanofibers.

Catalyst	Support Material	Catalytic Process	Catalytic Performance	Ref.
β-galactosidase immobilized on electrospun polystyrene nanofibers	Electrospun polystyrene nanofibers (PSNF)	Lactose conversion to galacto-oligosaccharides (GOS) in a disc reactor	High bioconversion performance	[276]
Pt nanoparticles supported on porous nanofibers of antimony-doped tin oxide	Antimony-doped tin oxide nanofibers	Electrooxidation of methanol in the anode of a direct methanol fuel cells	Lower loss of electrochemical surface area and higher stability of the supported Pt catalyst compared to conventional Pt/C catalysts was observed	[277]
Pd species incorporated crosslinked chitosan/poly(methacrylic acid) composite nanofibers	Crosslinked chitosan/poly(methacrylic acid) composite nanofibers	Heck reaction of aromatic iodides with acrylates	Facile separation from the reaction mixture for 10 subsequent runs without loss of activity despite 5.8% leaching of the palladium in each cycle	[278]
Phytase immobilized polyvinyl alcohol-sodium alginate (PVA-SA) electrospun nanofibers	Polyvinyl alcohol-sodium alginate (PVA-SA) nanofibers	Hydrolysis of phosphate moieties	Higher thermal and pH stability, enhanced catalytic activity of phytase after immobilization into PVA-SA based nanofibers compared to the free phytase	[279]
Nitrogen-doped carbon nanofibers supported FeO_x_-based nano catalyst	N-doped carbon nanofiber produced via pyrolysis of electrospun Fe-based MOF containing polyacrylonitrile and polymethylmethacrylate	Hydrogenation of nitroarenes to arylamines	Ease of recovery, reusability, higher activity, and chemoselectivity in reduction of functionalized nitroarenes compared to non-supported catalyst	[280]

## References

[1] Jamshidi M., Askarzadeh A. (2019). Techno-economic analysis and size optimization of an off-grid hybrid photovoltaic, fuel cell and diesel generator system. Sustain. Cities Soc..

[2] Zerta M., Schmidt P.R., Stiller C., Landinger H. (2008). Alternative World Energy Outlook (AWEO) and the role of hydrogen in a changing energy landscape. Int. J. Hydrogen Energy.

[3] Zhang X., Estoque R.C., Murayama Y. (2017). An urban heat island study in Nanchang City, China based on land surface temperature and social-ecological variables. Sustain. Cities Soc..

[4] Ahmad T., Zhang D. (2020). A critical review of comparative global historical energy consumption and future demand: The story told so far. Energy Rep..

[5] Wagner L., Ross I., Foster J., Hankamer B. (2016). Trading Off Global Fuel Supply, CO_2_ Emissions and Sustainable Development. PLoS ONE.

[6] Lu X., Wang C., Favier F., Pinna N. (2017). Electrospun nanomaterials for supercapacitor electrodes: Designed architectures and electrochemical performance. Adv. Energy Mater..

[7] Cheng X.-B., Zhang R., Zhao C.-Z., Zhang Q. (2017). Toward Safe Lithium Metal Anode in Rechargeable Batteries: A Review. Chem. Rev..

[8] Li L., Peng S., Lee J.K.Y., Ji D., Srinivasan M., Ramakrishna S. (2017). Electrospun hollow nanofibers for advanced secondary batteries. Nano Energy.

[9] Kumar P.S., Sundaramurthy J., Sundarrajan S., Babu V.J., Singh G., Allakhverdiev S.I., Ramakrishna S. (2014). Hierarchical electrospun nanofibers for energy harvesting, production and environmental remediation. Energy Environ. Sci..

[10] Khalid B., Bai X., Wei H., Huang Y., Wu H., Cui Y. (2017). Direct Blow-Spinning of Nanofibers on a Window Screen for Highly Efficient PM2.5 Removal. Nano Lett..

[11] Kang G.-D., Cao Y.-M. (2014). Application and modification of poly(vinylidene fluoride) (PVDF) membranes—A review. J. Membr. Sci..

[12] Homaeigohar S., Elbahri M. (2014). Nanocomposite Electrospun Nanofiber Membranes for Environmental Remediation. Materials.

[13] Gasparotto A., Barreca D., Maccato C., Tondello E. (2012). Manufacturing of inorganic nanomaterials: Concepts and perspectives. Nanoscale.

[14] Bhardwaj N., Kundu S.C. (2010). Electrospinning: A fascinating fiber fabrication technique. Biotechnol. Adv..

[15] Zhang L., Aboagye A., Kelkar A.D., Lai C., Fong H. (2014). A review: Carbon nanofibers from electrospun polyacrylonitrile and their applications. J. Mater. Sci..

[16] Zhang B., Kang F., Tarascon J.-M., Kim J.-K. (2016). Recent advances in electrospun carbon nanofibers and their application in electrochemical energy storage. Prog. Mater. Sci..

[17] Dong Z., Kennedy S.J., Wu Y. (2011). Electrospinning materials for energy-related applications and devices. J. Power Sources.

[18] Doyle J.J., Choudhari S.K., Ramakrishna S., Babu R.P. Electrospun Nanomaterials: Biotechnology, Food, Water, Environment, and Energy. Proceedings of the Conference Papers in Materials Science.

[19] Renugopalakrishnan E., Kannan A.M., Srinivasan S., Thavasi V., Ramakrishna P.S., Li A.M., Filipek A.S., Kumar J.D. (2009). Nanomaterials for Energy Conversion Applications.

[20] Santangelo S. (2019). Electrospun Nanomaterials for Energy Applications: Recent Advances. Appl. Sci..

[21] World Energy Outlook 2019. https://www.iea.org/reports/world-energy-outlook-2019.

[22] O’Regan B., Grätzel M., Gr M. (1991). A low-cost, high-efficiency solar cell based on dye-sensitized colloidal TiO_2_ films. Nature.

[23] Kakiage K., Aoyama Y., Yano T., Oya K., Fujisawa J.-I., Hanaya M. (2015). Highly-efficient dye-sensitized solar cells with collaborative sensitization by silyl-anchor and carboxy-anchor dyes. Chem. Commun..

[24] Zhang Q., Cao G. (2011). Nanostructured photoelectrodes for dye-sensitized solar cells. Nano Today.

[25] Iftikhar H., Sonai G.G., Hashmi S.G., Nogueira A.F., Lund P.D. (2019). Progress on Electrolytes Development in Dye-Sensitized Solar Cells. Materials.

[26] Hu L., Dai S., Weng J., Xiao S., Sui Y., Huang Y., Chen S., Kong F., Pan X., Liang L. (2007). Microstructure Design of Nanoporous TiO_2_Photoelectrodes for Dye-Sensitized Solar Cell Modules. J. Phys. Chem. B.

[27] Chen H.-Y., Kuang D.-B., Su C.-Y. (2012). Hierarchically micro/nanostructured photoanode materials for dye-sensitized solar cells. J. Mater. Chem..

[28] Chen T., Qiu L., Cai Z., Gong F., Yang Z., Wang Z., Peng H. (2012). Intertwined Aligned Carbon Nanotube Fiber Based Dye-Sensitized Solar Cells. Nano Lett..

[29] Zhang Q., Christopher S., Zhou X., Cao G. (2009). ZnO Nanostructures for Dye-Sensitized Solar Cells. Adv. Mater..

[30] Feng H.-L., Wu W.-Q., Rao H.-S., Wan Q., Li L.-B., Kuang D., Su C.-Y. (2015). Three-Dimensional TiO_2_/ZnO Hybrid Array as a Heterostructured Anode for Efficient Quantum-Dot-Sensitized Solar Cells. ACS Appl. Mater. Interfaces.

[31] Mukherjee K., Teng T.-H., Jose R., Ramakrishna S. (2009). Electron transport in electrospun TiO_2_ nanofiber dye-sensitized solar cells. Appl. Phys. Lett..

[32] Yang L., Leung W.W.-F. (2013). Electrospun TiO_2_ Nanorods with Carbon Nanotubes for Efficient Electron Collection in Dye-Sensitized Solar Cells. Adv. Mater..

[33] Francis L., Nair A.S., Jose R., Ramakrishna S., Thavasi V., Marsano E. (2011). Fabrication and characterization of dye-sensitized solar cells from rutile nanofibers and nanorods. Energy.

[34] Song M.Y., Kim D.K., Jo S.M., Kim D.Y. (2005). Enhancement of the photocurrent generation in dye-sensitized solar cell based on electrospun TiO_2_ electrode by surface treatment. Synth. Met..

[35] Krishnamoorthy T., Thavasi V.G., Ramakrishna S. (2011). A first report on the fabrication of vertically aligned anatase TiO_2_ nanowires by electrospinning: Preferred architecture for nanostructured solar cells. Energy Environ. Sci..

[36] Elumalai N.K., Jin T.M., Chellappan V., Jose R., Palaniswamy S.K., Jayaraman S., Raut H.K., Ramakrishna S. (2013). Electrospun ZnO Nanowire Plantations in the Electron Transport Layer for High-Efficiency Inverted Organic Solar Cells. ACS Appl. Mater. Interfaces.

[37] Hieu N.T., Baik S.J., Jun Y., Lee M., Chung O.H., Park J.S. (2014). Electrospun coaxial titanium dioxide/carbon nanofibers for use in anodes of dye-sensitized solar cells. Electrochim. Acta.

[38] Joshi P., Zhang L., Davoux D., Zhu Z., Galipeau D., Fong H., Qiao Q. (2010). Composite of TiO_2_ nanofibers and nanoparticles for dye-sensitized solar cells with significantly improved efficiency. Energy Environ. Sci..

[39] Jean J., Chang S., Brown P.R., Cheng J.J., Rekemeyer P.H., Bawendi M.G., Gradečak S., Bulović V. (2013). ZnO Nanowire Arrays for Enhanced Photocurrent in PbS Quantum Dot Solar Cells. Adv. Mater..

[40] Krishnamoorthy T., Tang M.Z., Verma A., Nair A.S., Pliszka D., Subodh G., Ramakrishna S. (2012). A facile route to vertically aligned electrospun SnO_2_ nanowires on a transparent conducting oxide substrate for dye-sensitized solar cells. J. Mater. Chem..

[41] Song M.Y., Kim D.K., Ihn K.J., Jo S.M., Kim D.Y. (2004). Electrospun TiO_2_ electrodes for dye-sensitized solar cells. Nanotechnology.

[42] Mali S.S., Shim C.-S., Kim H., Patil J.V., Ahn D.H., Patil P., Hong C.K. (2015). Evaluation of various diameters of titanium oxide nanofibers for efficient dye sensitized solar cells synthesized by electrospinning technique: A systematic study and their application. Electrochim. Acta.

[43] Motlak M., Hamza A., Hammed M.G., Barakat N.A.M. (2019). Cd-doped TiO_2_ nanofibers as effective working electrode for the dye sensitized solar cells. Mater. Lett..

[44] Yuan H., Jiao Q., Liu J., Liu X., Yang H., Zhao Y., Wu Q., Shi D., Li H. (2016). Ultrathin-walled Co9S8 nanotube/reduced graphene oxide composite as an efficient electrocatalyst for the reduction of triiodide. J. Power Sources.

[45] Chen M., Shao L.-L. (2016). Review on the recent progress of carbon counter electrodes for dye-sensitized solar cells. Chem. Eng. J..

[46] Cho S., Hwang S.H., Kim C., Jang J. (2012). Polyaniline porous counter-electrodes for high performance dye-sensitized solar cells. J. Mater. Chem..

[47] Trevisan R., Döbbelin M., Boix P.P., Barea E.M., Tena-Zaera R., Mora-Seró I., Bisquert J. (2011). PEDOT Nanotube Arrays as High Performing Counter Electrodes for Dye Sensitized Solar Cells. Study of the Interactions Among Electrolytes and Counter Electrodes. Adv. Energy Mater..

[48] Wu M., Zhang Q., Xiao J., Ma C., Lin X., He Y., Gao Y., Hagfeldt A., Ma T., Miao C. (2011). Two flexible counter electrodes based on molybdenum and tungsten nitrides for dye-sensitized solar cells. J. Mater. Chem..

[49] Jiang Q.W., Li G.R., Gao X. (2009). Highly ordered TiN nanotube arrays as counter electrodes for dye-sensitized solar cells. Chem. Commun..

[50] Li G.-R., Wang F., Jiang Q.-W., Gao X., Shen P.-W. (2010). Carbon Nanotubes with Titanium Nitride as a Low-Cost Counter-Electrode Material for Dye-Sensitized Solar Cells. Angew. Chem. Int. Ed..

[51] Huang X., Huang S., Zhang Q., Guo X., Li N., Luo Y., Shen Q., Toyoda T., Meng Q. (2011). A flexible photoelectrode for CdS/CdSe quantum dot-sensitized solar cells (QDSSCs). Chem. Commun..

[52] Wu M., Lin X., Wang Y., Wang L., Guo W., Qi D., Peng X., Hagfeldt A., Grätzel M., Ma T. (2012). Economical Pt-Free Catalysts for Counter Electrodes of Dye-Sensitized Solar Cells. J. Am. Chem. Soc..

[53] Xin X., He M., Han W., Jung J., Lin Z. (2011). Low-Cost Copper Zinc Tin Sulfide Counter Electrodes for High-Efficiency Dye-Sensitized Solar Cells. Angew. Chem. Int. Ed..

[54] Sun H., Qin D., Huang S., Guo X., Li D., Luo Y., Meng Q. (2011). Dye-sensitized solar cells with NiS counter electrodes electrodeposited by a potential reversal technique. Energy Environ. Sci..

[55] Thomas S., Deepak T.G., Anjusree G.S., Arun T.A., Nair S.V., Nair A.S. (2014). A review on counter electrode materials in dye-sensitized solar cells. J. Mater. Chem. A.

[56] Malara F., Manca M., De Marco L., Pareo P., Gigli G. (2011). Flexible Carbon Nanotube-Based Composite Plates as Efficient Monolithic Counter Electrodes for Dye Solar Cells. ACS Appl. Mater. Interfaces.

[57] Dong P., Pint C.L., Hainey M., Mirri F., Zhan Y., Zhang J., Pasquali M., Hauge R.H., Verduzco R., Jiang M. (2011). Vertically Aligned Single-Walled Carbon Nanotubes as Low-cost and High Electrocatalytic Counter Electrode for Dye-Sensitized Solar Cells. ACS Appl. Mater. Interfaces.

[58] Joshi P., Zhang L., Chen Q., Galipeau D., Fong H., Qiao Q. (2010). Electrospun Carbon Nanofibers as Low-Cost Counter Electrode for Dye-Sensitized Solar Cells. ACS Appl. Mater. Interfaces.

[59] Park S.-H., Kim B.-K., Lee W.-J. (2013). Electrospun activated carbon nanofibers with hollow core/highly mesoporous shell structure as counter electrodes for dye-sensitized solar cells. J. Power Sources.

[60] Song L., Chen P., Li Z., Du P., Yang Y., Li N., Xiong J. (2020). Flexible carbon nanotubes/TiO_2_/C nanofibrous film as counter electrode of flexible quasi-solid dye-sensitized solar cells. Thin Solid Films.

[61] Yousef A., Brooks R.M., El-Newehy M.H., Al-Deyab S.S., Kim H.Y. (2017). Electrospun Co-TiC nanoparticles embedded on carbon nanofibers: Active and chemically stable counter electrode for methanol fuel cells and dye-sensitized solar cells. Int. J. Hydrogen Energy.

[62] Best Research-Cell Efficiency Chart—Photovoltaic Research—NREL. https://www.nrel.gov/pv/cell-efficiency.html.

[63] Petridis C., Kakavelakis G., Kymakis E. (2018). Renaissance of graphene-related materials in photovoltaics due to the emergence of metal halide perovskite solar cells. Energy Environ. Sci..

[64] Yang W.S., Noh J.H., Jeon N.J., Kim Y.C., Ryu S., Seo J., Seok S.I. (2015). High-performance photovoltaic perovskite layers fabricated through intramolecular exchange. Science.

[65] Bi D., Tress W., Dar M.I., Gao P., Luo J., Renevier C., Schenk K., Abate A., Giordano F., Baena J.-P.C. (2016). Efficient luminescent solar cells based on tailored mixed-cation perovskites. Sci. Adv..

[66] Kim H.-S., Mora-Sero I., Gonzalez-Pedro V., Fabregat-Santiago F., Pérez E.J.J., Park N.-G., Bisquert J. (2013). Mechanism of carrier accumulation in perovskite thin-absorber solar cells. Nat. Commun..

[67] Stranks S.D., Eperon G.E., Grancini G., Menelaou C., Alcocer M.J.P., Leijtens T., Herz L.M., Petrozza A., Snaith H.J. (2013). Electron-Hole Diffusion Lengths Exceeding 1 Micrometer in an Organometal Trihalide Perovskite Absorber. Science.

[68] Kojima A., Teshima K., Shirai Y., Miyasaka T. (2009). Organometal Halide Perovskites as Visible-Light Sensitizers for Photovoltaic Cells. J. Am. Chem. Soc..

[69] Kim H.S., Lee C.R., Im J.H., Lee K.B., Moehl T., Marchioro A., Moon S.J., Humphry-Baker R., Yum J.H., Moser J.E. (2012). Lead iodide perovskite sensitized all-solidstate submicron thin film mesoscopic solar cell with efficiency exceeding 9%. Sci. Rep..

[70] Dharani S., Mulmudi H.K., Yantara N., Trang P.T.T., Park N.G., Graetzel M., Mhaisalkar S., Mathews N., Boix P.P. (2014). High efficiency electrospun TiO_2_nanofiber based hybrid organic–inorganic perovskite solar cell. Nanoscale.

[71] Lu J., Zhang L., Peng C., Rao L., Wan M. (2016). Preparation and Characterization of CH_3_NH_3_PbI_3_ Perovskite Deposited onto Polyacrylonitrile (PAN) Nanofiber Substrates. Chem. Lett..

[72] Patil J.V., Mali S.S., Patil A.P., Patil P.S., Hong C.K. (2019). Highly efficient mixed-halide mixed-cation perovskite solar cells based on rGO-TiO_2_ composite nanofibers. Energy.

[73] Li Q., Balilonda A., Ali A., Jose R., Zabihi F., Yang S., Ramakrishna S., Zhu M. (2020). Flexible Solar Yarns with 15.7% Power Conversion Efficiency, Based on Electrospun Perovskite Composite Nanofibers. Sol. RRL.

[74] Kabir S., Medina S., Wang G., Bender G., Pylypenko S., Neyerlin K.C. (2020). Improving the bulk gas transport of Fe-N-C platinum group metal-free nanofiber electrodes via electrospinning for fuel cell applications. Nano Energy.

[75] He Y., Guo H., Hwang S., Yang X., He Z., Braaten J., Karakalos S., Shan W., Wang M., Zhou H. (2020). Single Cobalt Sites Dispersed in Hierarchically Porous Nanofiber Networks for Durable and High-Power PGM-Free Cathodes in Fuel Cells. Adv. Mater..

[76] Ponomarev I.I., Skupov K.M., Zhigalina O.M., Naumkin A.V., Modestov A.D., Basu V.G., Sufiyanova A.E., Razorenov D.Y., Ponomarev I.I. (2020). New Carbon Nanofiber Composite Materials Containing Lanthanides and Transition Metals Based on Electrospun Polyacrylonitrile for High Temperature Polymer Electrolyte Membrane Fuel Cell Cathodes. Polymers.

[77] He Y., Wang D., Li Q., Huang L., Bao H. (2020). Composite Polymer Electrolyte Membranes based on Nafion and Modified PVDF Electrospun Nanofiber Mats. J. Wuhan Univ. Technol. Sci. Ed..

[78] Liu G., Tsen W.-C., Jang S.-C., Hu F., Zhong F., Zhang B., Wang J., Liu H., Wang G., Wen S. (2020). Composite membranes from quaternized chitosan reinforced with surface-functionalized PVDF electrospun nanofibers for alkaline direct methanol fuel cells. J. Membr. Sci..

[79] Ikram S., Ahmed S., Ali S.W., Agarwal H. (2017). Chitosan-Based Polymer Electrolyte Membranes for Fuel Cell Applications. Organic-Inorganic Composite Polymer Electrolyte Membranes.

[80] Slate A.J., Whitehead K.A., Brownson D.A., Banks C.E. (2019). Microbial fuel cells: An overview of current technology. Renew. Sustain. Energy Rev..

[81] Gao Y., Mohammadifar M., Choi S. (2019). From Microbial Fuel Cells to Biobatteries: Moving toward On-Demand Micropower Generation for Small-Scale Single-Use Applications. Adv. Mater. Technol..

[82] Li S., Chen G. (2018). Factors Affecting the Effectiveness of Bioelectrochemical System Applications: Data Synthesis and Meta-Analysis. Batteries.

[83] Baptista A.C., Martins J.I., Fortunato E., Martins R., Borges J.P., Ferreira I. (2010). Thin and flexible bio-batteries made of electrospun cellulose-based membranes. Biosens. Bioelectron.

[84] Massaglia G., Frascella F., Chiadò A., Sacco A., Marasso S.L., Cocuzza M., Pirri C.F., Quaglio M. (2020). Electrospun Nanofibers: From Food to Energy by Engineered Electrodes in Microbial Fuel Cells. Nanomaterials.

[85] Thackeray M.M., Wolverton C.M., Isaacs E.D. (2012). Electrical energy storage for transportation—Approaching the limits of, and going beyond, lithium-ion batteries. Energy Environ. Sci..

[86] Aravindan V., Gnanaraj J., Lee Y.-S., Madhavi S. (2013). LiMnPO_4_—A next generation cathode material for lithium-ion batteries. J. Mater. Chem. A.

[87] Obrovac M.N., Chevrier V.L. (2014). Alloy Negative Electrodes for Li-Ion Batteries. Chem. Rev..

[88] Kavan L. (2011). Electrochemistry of titanium dioxide: Some aspects and highlights. Chem. Rec..

[89] Kim C., Yang K.-S., Kojima M., Yoshida K., Kim Y.J., Endo M. (2006). Fabrication of Electrospinning-Derived Carbon Nanofiber Webs for the Anode Material of Lithium-Ion Secondary Batteries. Adv. Funct. Mater..

[90] Zhang B., Yu Y., Xu Z.L., Abouali S., Akbari M., He Y.B., Kang F., Kim J.K. (2014). Correlation between atomic structure and electrochemical performance of anodes made from electrospun carbon nanofiber films. Adv. Energy Mater.

[91] Han W., Xiao Y., Yin J., Gong Y., Tuo X., Cao J. (2020). Fe_3_O_4_@Carbon Nanofibers Synthesized from Cellulose Acetate and Application in Lithium-Ion Battery. Langmuir.

[92] Hu J., Wang H., Qin C., Li Y., Yang Y. (2020). Fabrication of TiO_2_@C/N composite nanofibers and application as stable lithium-ion battery anode. Mater. Lett..

[93] Xu G.-L., Wang Q., Fang J.-C., Xu Y.-F., Li J.-T., Huang L., Sun S.-G. (2014). Tuning the structure and property of nanostructured cathode materials of lithium ion and lithium sulfur batteries. J. Mater. Chem. A.

[94] Scrosati B., Garche J. (2010). Lithium batteries: Status, prospects and future. J. Power Sources.

[95] Gu Y., Chen D., Jiao X. (2005). Synthesis and Electrochemical Properties of Nanostructured LiCoO_2_Fibers as Cathode Materials for Lithium-Ion Batteries. J. Phys. Chem. B.

[96] Gu Y., Chen D., Jiao X., Liu F. (2007). LiCoO_2_–MgO coaxial fibers: Co-electrospun fabrication, characterization and electrochemical properties. J. Mater. Chem..

[97] Liu Y., Taya M. Electrospinning fabrication and electrochemical properties of lithium cobalt nanofibers for lithium battery cathode. Proceedings of the SPIE Smart Structures and Materials + Nondestructive Evaluation and Health Monitoring.

[98] Wang Z., Xu K., Zhang Y., Wu J., Lin X., Liu C., Hua J. (2020). Study on electro-spin performance of different types of cellulose by activation in the solvent of LiCl/DMAc. J. For. Eng..

[99] Zhu C., Yu Y., Gu L., Weichert K., Maier J. (2011). Electrospinning of Highly Electroactive Carbon-Coated Single-Crystalline LiFePO_4_ Nanowires. Angew. Chem..

[100] Cheah Y.L., Gupta N., Pramana S.S., Aravindan V., Wee G., Madhavi S. (2011). Morphology, structure and electrochemical properties of single phase electrospun vanadium pentoxide nanofibers for lithium ion batteries. J. Power Sources.

[101] Toprakci O., Ji L., Lin Z., Toprakci H.A., Zhang X. (2011). Fabrication and electrochemical characteristics of electrospun LiFePO4/carbon composite fibers for lithium-ion batteries. J. Power Sources.

[102] Mai L.-Q., Xu L., Han C., Xu X., Luo Y., Zhao S., Zhao Y. (2010). Electrospun Ultralong Hierarchical Vanadium Oxide Nanowires with High Performance for Lithium Ion Batteries. Nano Lett..

[103] Zheng D., Zhang X., Wang J., Qu D., Yang X., Qu D. (2016). Reduction mechanism of sulfur in lithium–sulfur battery: From elemental sulfur to polysulfide. J. Power Sources.

[104] Pope M.A., Aksay I.A. (2015). Structural Design of Cathodes for Li-S Batteries. Adv. Energy Mater..

[105] Wang J., Yang Y., Kang F. (2015). Porous carbon nanofiber paper as an effective interlayer for high-performance lithium-sulfur batteries. Electrochim. Acta.

[106] Wang H., Zhang C., Chen Z., Liu H.K., Guo Z. (2015). Large-scale synthesis of ordered mesoporous carbon fiber and its application as cathode material for lithium–sulfur batteries. Carbon.

[107] Wu Y., Gao M., Li X., Liu Y.-F., Pan H. (2014). Preparation of mesohollow and microporous carbon nanofiber and its application in cathode material for lithium–sulfur batteries. J. Alloys Compd..

[108] Zhang Y.-Z., Wu Z.-Z., Pan G.-L., Liu S., Gao X. (2017). Microporous Carbon Polyhedrons Encapsulated Polyacrylonitrile Nanofibers as Sulfur Immobilizer for Lithium–Sulfur Battery. ACS Appl. Mater. Interfaces.

[109] Rauloa A., Bandyopadhyaya S., Ahamadb S., Gupta A., Srivastavaa R., Formanekc P., Nandan B. (2019). Bio-inspired poly(3,4-ethylenedioxythiophene): Poly(styrene sulfonate)-sulfur@polyacrylonitrile electrospun nanofibers for lithium-sulfur batteries. J. Power Sources.

[110] Tong Z., Huang L., Lei W., Zhang H., Zhang S. (2021). Carbon-containing electrospun nanofibers for lithium–sulfur battery: Current status and future directions. J. Energy Chem..

[111] Peng S., Ilango P.R. (2020). Electrospinning of Nanofibers for Li-Air Battery. Electrospinning of Nanofibers for Battery Applications.

[112] Perathoner S., Centi G. (2018). Chapter 9—Advanced Nanocarbon Materials for Future Energy Applications. Emerging Materials for Energy Conversion and Storage.

[113] Tsou Y.-H., Chuang Y.-Y., Chen J.-S. (2020). Effect of surface bonding of FePC with electrospun carbon nanofiber on electrocatalytic performance for aprotic Li-O_2_ batteries. J. Colloid Interface Sci..

[114] Zhaoa L., Xingb Y., Chena N., Laia J., Lia L., Wua F., Chena R. (2020). A robust cathode of RuO_2_ nH_2_O clusters anchored on the carbon nanofibers for ultralong-life lithium-oxygen batteries. J. Power Sources.

[115] Sun M., Guo S., Wang Z., Zou L., Chi B., Pu J., Li J. (2020). Novel and highly efficient catalyst for Li–O_2_ battery: Porous LaCo0.6Ni0.4O3 nanofibers decorated with ultrafine Co_3_O_4_ nanoparticles. Electrochim. Acta.

[116] Chen X., Paul R., Dai L. (2017). Carbon-based supercapacitors for efficient energy storage. Natl. Sci. Rev..

[117] Wei K., Kim I.S. (2014). Application of Nanofibers in Supercapacitors. Surf. Eff. Magn. Nanopart..

[118] Liang J., Zhao H., Yue L., Fan G., Li T.S., Lu S., Chen G., Gao S., Asiri A.M., Sun X. (2020). Recent advances in electrospun nanofibers for supercapacitors. J. Mater. Chem. A.

[119] Pant B., Pant H.R., Park M. (2020). Fe_1−x_S Modified TiO_2_ NPs Embedded Carbon Nanofiber Composite via Electrospinning: A Potential Electrode Material for Supercapacitors. Molecules.

[120] Kim J., Heo Y.-J., Hong J.-Y., Kim S.-K. (2020). Preparation of Porous Carbon Nanofibers with Tailored Porosity for Electrochemical Capacitor Electrodes. Materials.

[121] Jeon S., Jeong J.H., Yoo H., Yu H.K., Kim B.-H., Kim M.H. (2020). RuO_2_ Nanorods on Electrospun Carbon Nanofibers for Supercapacitors. ACS Appl. Nano Mater..

[122] Yang S., Ai J., Han Z., Zhang L., Zhao D., Wang J., Yang C., Cao B. (2020). Electrospun ZnFe_2_O_4_/carbon nanofibers as high-rate supercapacitor electrodes. J. Power Sources.

[123] Wang X., Yu J., Sun G., Ding B. (2016). Electrospun nanofibrous materials: A versatile medium for effective oil/water separation. Mater. Today.

[124] Lin J., Shang Y., Ding B., Yang J., Yu J., Al-Deyab S.S. (2012). Nanoporous polystyrene fibers for oil spill cleanup. Mar. Pollut. Bull..

[125] Sarbatly R., Duduku K., Kamin Z. (2016). A review of polymer nanofibres by electrospinning and their application in oil–water separation for cleaning up marine oil spills. Mar. Pollut. Bull..

[126] Liu L., Lin Z., Niu J., Tian D., He J. (2019). Electrospun polysulfone/poly(lactic acid) nanoporous fibrous mats for oil removal from water. Adsorpt. Sci. Technol..

[127] Blinovskaya Y. (2019). Efficiency Sorbents Comparative Analysis for Heavy Oil Products in the Conditions of Low Temperatures. IOP Conf. Ser. Earth Environ. Sci..

[128] Ma W., Zhang Q., Hua D., Xiong R., Zhao J., Rao W., Huang S., Zhan X., Chen F., Huang C. (2016). Electrospun fibers for oil–water separation. RSC Adv..

[129] Lee M.W., An S., Latthe S.S., Lee C., Hong S., Yoon S.S. (2013). Electrospun Polystyrene Nanofiber Membrane with Superhydrophobicity and Superoleophilicity for Selective Separation of Water and Low Viscous Oil. ACS Appl. Mater. Interfaces.

[130] Feng L., Zhang Z., Mai Z., Ma Y., Liu B., Jiang L., Zhu D. (2004). A Super-Hydrophobic and Super-Oleophilic Coating Mesh Film for the Separation of Oil and Water. Angew. Chem. Int. Ed..

[131] Alnaqbi M.A., Al Blooshi A.G., Greish Y.E. (2020). Polyethylene and Polyvinyl Chloride-Blended Polystyrene Nanofibrous Sorbents and Their Application in the Removal of Various Oil Spills. Adv. Polym. Technol..

[132] Sundaran S.P., Sujith A. (2017). Fabrication of superhydrophobic polycaprolactone/beeswax electrospun membranes for high-efficiency oil/water separation. RSC Adv..

[133] Jiang Z., Tijing L.D., Amarjargal A., Park C.H., An K.-J., Shon H.K., Kim C. (2015). Removal of oil from water using magnetic bicomponent composite nanofibers fabricated by electrospinning. Compos. Part. B Eng..

[134] Behera A., Sahu P., Mohapatra S., Ghadei S. (2020). Characterization of carbon based nanofibers in nanocomposites and their applications. Mater. Today: Proc..

[135] Noamani S., Niroomand S., Shakeri A., Sadrzadeh M. (2019). Carbon-based polymer nanocomposite membranes for oily wastewater treatment. NPJ Clean Water.

[136] Liu H., Cao C.-Y., Wei F.-F., Huang P.-P., Sun Y., Jiang L., Song W.-G. (2014). Flexible macroporous carbon nanofiber film with high oil adsorption capacity. J. Mater. Chem. A.

[137] Lin Y.-Z., Zhong L.-B., Dou S., Shao Z.-D., Liu Q., Zheng Y.-M. (2019). Facile synthesis of electrospun carbon nanofiber/graphene oxide composite aerogels for high efficiency oils absorption. Environ. Int..

[138] Kwon G., Kota A.K., Li Y., Sohani A., Mabry J.M., Tuteja A. (2012). On-Demand Separation of Oil-Water Mixtures. Adv. Mater..

[139] Focarete M.L., Gualandi C., Ramakrishna S. (2018). Filtering Media by Electrospinning: Next Generation Membranes for Separation Applications.

[140] Ma W., Zhang M., Liu Z., Kang M., Huang C., Fu G. (2019). Fabrication of highly durable and robust superhydrophobic-superoleophilic nanofibrous membranes based on a fluorine-free system for efficient oil/water separation. J. Membr. Sci..

[141] Ma W., Li Y., Zhang M., Gao S., Cui J., Huang C., Fu G. (2020). Biomimetic Durable Multifunctional Self-Cleaning Nanofibrous Membrane with Outstanding Oil/Water Separation, Photodegradation of Organic Contaminants, and Antibacterial Performances. ACS Appl. Mater. Interfaces.

[142] Gupta R.K., Dunderdale G.J., England M.W., Hozumi A. (2017). Oil/water separation techniques: A review of recent progresses and future directions. J. Mater. Chem. A.

[143] Ding B., Wang X., Yu J. (2019). Electrospinning: Nanofabrication and Applications.

[144] Xue Z., Cao Y., Liu N., Feng L., Jiang L. (2014). Special wettable materials for oil/water separation. J. Mater. Chem. A.

[145] Huang M., Si Y., Tang X., Zhu Z., Ding B., Liu L., Zheng G., Luo W., Yu J. (2013). Gravity driven separation of emulsified oil–water mixtures utilizing in situ polymerized superhydrophobic and superoleophilic nanofibrous membranes. J. Mater. Chem. A.

[146] Moatmed S.M., Khedr M.H., El-Dek S., Kim H.Y., El-Deen A.G. (2019). Highly efficient and reusable superhydrophobic/superoleophilic polystyrene@ Fe_3_O_4_ nanofiber membrane for high-performance oil/water separation. J. Environ. Chem. Eng..

[147] Wang J.-C., Lou H., Cui Z.-H., Hou Y., Li Y., Zhang Y., Jiang K., Shi W., Qu L. (2019). Fabrication of porous polyacrylamide/polystyrene fibrous membranes for efficient oil-water separation. Sep. Purif. Technol..

[148] World Health Organization Chemicals of Major Public Health Concern. https://www.who.int/ipcs/features/chemicals_concern/en/.

[149] Wang M., Hossain F., Sulaiman R., Ren X. (2019). Exposure to Inorganic Arsenic and Lead and Autism Spectrum Disorder in Children: A Systematic Review and Meta-Analysis. Chem. Res. Toxicol..

[150] Nurchi V.M., Djordjevic A.B., Crisponi G., Alexander J., Bjørklund G., Aaseth J. (2020). Arsenic Toxicity: Molecular Targets and Therapeutic Agents. Biomolecules.

[151] Genchi G., Sinicropi M.S., Lauria G., Carocci A., Catalano A. (2020). The Effects of Cadmium Toxicity. Int. J. Environ. Res. Public Health.

[152] Cariccio V.L., Samà A., Bramanti P., Mazzon E. (2018). Mercury Involvement in Neuronal Damage and in Neurodegenerative Diseases. Biol. Trace Elem. Res..

[153] Masindi V., Muedi K.L. (2018). Environmental Contamination by Heavy Metals. Heavy Met..

[154] Zang L., Lin R., Dou T., Wang L.W.L., Ma J., Sun L. (2019). Electrospun superhydrophilic membranes for effective removal of Pb(ii) from water. Nanoscale Adv..

[155] Peng L., Zhang X., Sun Y., Xing Y., Li C. (2020). Heavy metal elimination based on metal organic framework highly loaded on flexible nanofibers. Environ. Res..

[156] Atashgahi S., Shetty S.A., Smidt H., De Vos W.M. (2018). Flux, Impact, and Fate of Halogenated Xenobiotic Compounds in the Gut. Front. Physiol..

[157] El-Shahawi M., Hamza A., Bashammakh A., Al-Saggaf W. (2010). An overview on the accumulation, distribution, transformations, toxicity and analytical methods for the monitoring of persistent organic pollutants. Talanta.

[158] Genuis S.K., Birkholz D., Genuis S.J. (2017). Human Excretion of Polybrominated Diphenyl Ether Flame Retardants: Blood, Urine, and Sweat Study. BioMed Res. Int..

[159] Senthamizhan A., Balusamy B., Uyar T. (2018). Electrospun Filters for Organic Pollutants Removal.

[160] Ligneris E.D., Dumée L.F., Kong L. (2018). Nanofiber-Based Materials for Persistent Organic Pollutants in Water Remediation by Adsorption. Appl. Sci..

[161] Najafi M., Frey M.W. (2020). Electrospun Nanofibers for Chemical Separation. Nanomaterials.

[162] Ehteshami S., Feizbakhsh A., Sarrafi A.H.M., Panahi H.A., Roostaie A., Feiabakhsh A. (2018). An electrospun polyamide/graphene oxide nanocomposite as a novel fiber coating. Anal. Methods.

[163] Celebioglu A., Topuz F., Yildiz Z.I., Uyar T. (2019). Efficient Removal of Polycyclic Aromatic Hydrocarbons and Heavy Metals from Water by Electrospun Nanofibrous Polycyclodextrin Membranes. ACS Omega.

[164] Ardekani R., Borhani S., Rezaei B. (2020). Selective molecularly imprinted polymer nanofiber sorbent for the extraction of bisphenol A in a water sample. Polym. Int..

[165] Yu D., Bai J., Liang H., Ma T., Li C. (2016). AgI-modified TiO_2_ supported by PAN nanofibers: A heterostructured composite with enhanced visible-light catalytic activity in degrading MO. Dyes Pigm..

[166] Xu T., Wu F., Gu Y., Chen Y., Cai J., Lu W., Hu H., Zhu Z., Chen W. (2015). Visible-light responsive electrospun nanofibers based on polyacrylonitrile-dispersed graphitic carbon nitride. RSC Adv..

[167] Panthi G., Gyawali K.R., Park M. (2020). Towards the Enhancement in Photocatalytic Performance of Ag_3_PO_4_ Nanoparticles through Sulfate Doping and Anchoring on Electrospun Nanofibers. Nanomaterials.

[168] Liang H., Bai J., Xu T., Li C. (2021). Enhancing photocatalytic performance of heterostructure MoS_2_/g-C_3_N_4_ embeded in PAN frameworks by electrospining process. Mater. Sci. Semicond. Process..

[169] Sundaran S.P., Reshmi C.R., Sagitha P., Sujith A. (2020). Polyurethane nanofibrous membranes decorated with reduced graphene oxide–TiO_2_ for photocatalytic templates in water purification. J. Mater. Sci..

[170] Lakshmi K., Kadirvelu K., Mohan P.S. (2019). Chemically modified electrospun nanofiber for high adsorption and effective photocatalytic decontamination of organophosphorus compounds. J. Chem. Technol. Biotechnol..

[171] Kumar R., Ahmed M., Bhadrachari G., Thomas J.P. (2018). Desalination for agriculture: Water quality and plant chemistry, technologies and challenges. Water Supply.

[172] Wang Z., Sahadevan R., Crandall C., Menkhaus T.J., Fong H. (2020). Hot-pressed PAN/PVDF hybrid electrospun nanofiber membranes for ultrafiltration. J. Membr. Sci..

[173] Al Aani S., Haroutounian A., Wright C., Hilal N. (2018). Thin Film Nanocomposite (TFN) membranes modified with polydopamine coated metals/carbon-nanostructures for desalination applications. Desalination.

[174] Shahmirzadi M.A.A., Kargari A. (2018). Nanocomposite membranes. Emerging Technologies for Sustainable Desalination Handbook.

[175] Gonzales R.R., Park M.J., Tijing L.D., Han D.S., Phuntsho S., Shon H. (2018). Modification of Nanofiber Support Layer for Thin Film Composite forward Osmosis Membranes via Layer-by-Layer Polyelectrolyte Deposition. Membranes.

[176] Idarraga-Mora J.A., Childress A.S., Friedel P.S., Ladner D.A., Rao A.M., Husson S.M. (2018). Role of Nanocomposite Support Stiffness on TFC Membrane Water Permeance. Membranes.

[177] Chen H., Huang M., Liu Y., Meng L., Ma M. (2020). Functionalized electrospun nanofiber membranes for water treatment: A review. Sci. Total Environ..

[178] Van Der Bruggen B., Vandecasteele C., Van Gestel T., Doyen W., Leysen R. (2003). A review of pressure-driven membrane processes in wastewater treatment and drinking water production. Environ. Prog..

[179] Shirazi M.M.A., Bazgir S., Meshkani F., Abdelrasoul A. (2020). Electrospun Nanofibrous Membranes for Water Treatment. Advances in Membrane Technologies.

[180] Wang Z., Crandall C., Sahadevan R., Menkhaus T.J., Fong H. (2017). Microfiltration performance of electrospun nanofiber membranes with varied fiber diameters and different membrane porosities and thicknesses. Polymers.

[181] Tang N., Si Y., Yu J., Ding B. (2020). Leaf vein-inspired microfiltration membrane based on ultrathin nanonetworks. Environ. Sci. Nano.

[182] Li M., Li J., Zhou M., Xian Y., Shui Y., Wu M., Yao Y. (2020). Super-hydrophilic electrospun PVDF/PVA-blended nanofiber membrane for microfiltration with ultrahigh water flux. J. Appl. Polym. Sci..

[183] Bolto B., Zhang J., Wu X., Xie Z. (2020). A Review on Current Development of Membranes for Oil Removal from Wastewaters. Membranes.

[184] Dobosz K.M., Kuo-Leblanc C.A., Martin T.J., Schiffman J.D. (2017). Ultrafiltration Membranes Enhanced with Electrospun Nanofibers Exhibit Improved Flux and Fouling Resistance. Ind. Eng. Chem. Res..

[185] Bahmani P., Maleki A., Daraei H., Khamforoush M., Rezaee R., Gharibi F., Tkachev A.G., Burakov A.E., Agarwal S., Gupta V.K. (2017). High-flux ultrafiltration membrane based on electrospun polyacrylonitrile nanofibrous scaffolds for arsenate removal from aqueous solutions. J. Colloid Interface Sci..

[186] Modesti M., Boaretti C., Roso M. (2015). Electrospun Nanofibers for Water and Wastewater Treatment Applications. Encyclopedia of Membranes.

[187] Singh R., Bhadouria R., Singh P., Kumar A., Pandey S., Singh V.K. (2020). Nanofiltration technology for removal of pathogens present in drinking water. Waterborne Pathog..

[188] Abdel-Fatah M.A. (2018). Nanofiltration systems and applications in wastewater treatment: Review article. AIN Shams Eng. J..

[189] Kaur S., Sundarrajan S., Rana D., Matsuura T., Ramakrishna S. (2012). Influence of electrospun fiber size on the separation efficiency of thin film nanofiltration composite membrane. J. Membr. Sci..

[190] Liu F., Wang L., Li D., Liu Q., Deng B. (2020). Preparation and characterization of novel thin film composite nanofiltration membrane with PVDF tree-like nanofiber membrane as composite scaffold. Mater. Des..

[191] Nakhowong R. (2020). Fabrication of PVDF/PVP nanofiltration membrane containing chitosan/activated carbon/Ag nanoparticles by electrospinning and their antibacterial activity. SNRUJST.

[192] Zhijiang C., Cong Z., Ping X., Jie G., Kongyin Z. (2018). Calcium alginate-coated electrospun polyhydroxybutyrate/carbon nanotubes composite nanofibers as nanofiltration membrane for dye removal. J. Mater. Sci..

[193] Ziolkowska J.R. (2015). Desalination leaders in the global market—Current trends and future perspectives. Water Supply.

[194] Jones E., Qadir M., Van Vliet M.T., Smakhtin V., Kang S.-M. (2019). The state of desalination and brine production: A global outlook. Sci. Total Environ..

[195] Berenguel F., Galera A.L. (2020). Requirements for the Construction of New Desalination Plants into a Framework of Sustainability. Sustainability.

[196] Lehmann O., Eckhaus O., Lahav O., Birnhack L. (2015). Replenishing Mg(II) to desalinated water by seawater nanofiltration followed by magnetic separation of Mg(OH)_2_(s)Fe_3_O_4_ particles. Desalination Water Treat..

[197] Wang X.-N., Liu Y., Pan X.-H., Han J.-X., Hao J. (2016). Parameters for Seawater Reverse Osmosis Product Water: A Review. Expo. Health.

[198] Raza M.A., Islam A., Sabir A., Gull N., Ali I., Mehmood R., Bae J., Hassan G., Khan M.U. (2019). PVA/TEOS crosslinked membranes incorporating zinc oxide nanoparticles and sodium alginate to improve reverse osmosis performance for desalination. J. Appl. Polym. Sci..

[199] Wang X., Ma H., Chu B., Hsiao B.S. (2017). Thin-film nanofibrous composite reverse osmosis membranes for desalination. Desalination.

[200] Mohammadifakhr M., De Grooth J., Roesink H.D.W., Kemperman A.J. (2020). Forward Osmosis: A Critical Review. Processes.

[201] Johnson D.J., Suwaileh W.A., Mohammed A.W., Hilal N. (2018). Osmotic’s potential: An overview of draw solutes for forward osmosis. Desalination.

[202] Mark P. (2013). General intro to Forward Osmosis Membranes and Processes—Forward OsmosisTech. https://www.forwardosmosistech.com/forward-osmosis-membranes-and-membrane-processes/.

[203] Haupt A., Lerch A. (2018). Forward Osmosis Application in Manufacturing Industries: A Short Review. Membranes.

[204] Lee S., Kim Y., Park J., Shon H., Hong S. (2018). Treatment of medical radioactive liquid waste using Forward Osmosis (FO) membrane process. J. Membr. Sci..

[205] Dou P., Zhao S., Xu S., Li X.-M., He T. (2020). Feasibility of osmotic dilution for recycling spent dialysate: Process performance, scaling, and economic evaluation. Water Res..

[206] Soo K.W., Wong K.C., Goh P.S., Ismail A.F., Othman N. (2020). Efficient heavy metal removal by thin film nanocomposite forward osmosis membrane modified with geometrically different bimetallic oxide. J. Water Process. Eng..

[207] Khulbe K., Matsuura T. (2019). Art to use electrospun nanofibers/nanofiber based membrane in waste water treatment, chiral separation and desalination. J. Membr. Sci. Res..

[208] Pan S.-F., Dong Y., Zheng Y.-M., Zhong L.-B., Yuan Z.-H. (2017). Self-sustained hydrophilic nanofiber thin film composite forward osmosis membranes: Preparation, characterization and application for simulated antibiotic wastewater treatment. J. Membr. Sci..

[209] Al-Furaiji M., Kadhom M., Kalash K., Waisi B., Albayati N. (2020). Preparation of TFC Membranes Supported with Electrospun Nanofibers for Desalination by Forward Osmosis. Drink. Water Eng. Sci. Discuss..

[210] Karanasiou A., Kostoglou M., Karabelas A.J. (2018). An Experimental and Theoretical Study on Separations by Vacuum Membrane Distillation Employing Hollow-Fiber Modules. Water.

[211] Said I.A., Fuentes N., He Z., Xin R., Zuo K., Li Q., Abdallah I. (2020). Low-cost desalination of seawater and hypersaline brine using nanophotonics enhanced solar energy membrane distillation. Environ. Sci. Water Res. Technol..

[212] He J., Zhang L., Zhang K., Qin Y., Liu L. (2015). Concentrating aqueous urea solution by using continuous-effect membrane distillation. Chem. Eng. Res. Des..

[213] Criscuoli A., Capuano A., Andreucci M., Drioli E. (2020). Low-Temperature Direct Contact Membrane Distillation for the Treatment of Aqueous Solutions Containing Urea. Membranes.

[214] Amaya-Vías D., López-Ramírez J.A. (2019). Techno-Economic Assessment of Air and Water Gap Membrane Distillation for Seawater Desalination under Different Heat Source Scenarios. Water.

[215] Membrane Distillation—EMIS. https://emis.vito.be/en/bat/tools-overview/sheets/membrane-distillation.

[216] Teoh M.M., Chung T.-S., Yeo Y.S. (2011). Dual-layer PVDF/PTFE composite hollow fibers with a thin macrovoid-free selective layer for water production via membrane distillation. Chem. Eng. J..

[217] Zuo J., Chung T.-S., O’Brien G.S., Kosar W. (2017). Hydrophobic/hydrophilic PVDF/Ultem^®^ dual-layer hollow fiber membranes with enhanced mechanical properties for vacuum membrane distillation. J. Membr. Sci..

[218] Khayet M.C., García-Payo L. (2018). García-Fernández, J. Contreras-Martínez. Dual-layered electrospun nanofibrous membranes for membrane distillation. Desalination.

[219] Woo Y.C., Tijing L.D., Park M.J., Yao M., Choi J.-S., Lee S., Kim S.-H., An K.-J., Shon H. (2017). Electrospun dual-layer nonwoven membrane for desalination by air gap membrane distillation. Desalination.

[220] Attia H., Johnson D.J., Wright C.J., Hilal N. (2018). Robust superhydrophobic electrospun membrane fabricated by combination of electrospinning and electrospraying techniques for air gap membrane distillation. Desalination.

[221] Deka B.J., Lee E.-J., Guo J., Kharraz J., An A.K. (2019). Electrospun Nanofiber Membranes Incorporating PDMS-Aerogel Superhydrophobic Coating with Enhanced Flux and Improved Antiwettability in Membrane Distillation. Environ. Sci. Technol..

[222] Matsubara M.E., Helwig K., Hunter C., Roberts J., Subtil E.L., Coelho L.H.G. (2020). Amoxicillin removal by pre-denitrification membrane bioreactor (A/O-MBR): Performance evaluation, degradation by-products, and antibiotic resistant bacteria. Ecotoxicol. Environ. Saf..

[223] Pathak N., Tran V.H., Merenda A., Johir M., Phuntsho S., Shon H. (2020). Removal of Organic Micro-Pollutants by Conventional Membrane Bioreactors and High-Retention Membrane Bioreactors. Appl. Sci..

[224] Mao X., Myavagh P.H., Lotfikatouli S., Hsiao B.S., Walker H.W. (2020). Membrane Bioreactors for Nitrogen Removal from Wastewater: A Review. J. Environ. Eng..

[225] Yang J., Gou Y., Fang F., Guo J., Lu L., Zhou Y., Ma H. (2018). Potential of wastewater treatment using a concentrated and suspended algal-bacterial consortium in a photo membrane bioreactor. Chem. Eng. J..

[226] Basile A., Cassano A., Rastogi N. (2015). Advances in Membrane Technologies for Water Treatment.

[227] Aslam M., Charfi A., Lesage G., Heran M., Kim J. (2017). Membrane bioreactors for wastewater treatment: A review of mechanical cleaning by scouring agents to control membrane fouling. Chem. Eng. J..

[228] Di Trapani D., Di Bella G., Mannina G., Torregrossa M., Viviani G. (2014). Comparison between moving bed-membrane bioreactor (MB-MBR) and membrane bioreactor (MBR) systems: Influence of wastewater salinity variation. Bioresour. Technol..

[229] Subtil E.L., Mierzwa J.C., Hespanhol I. (2014). Comparison between a conventional membrane bioreactor (C-MBR) and a biofilm membrane bioreactor (BF-MBR) for domestic wastewater treatment. Braz. J. Chem. Eng..

[230] Yang Y., Shao Z., Du J., He Q., Chai H. (2018). Enhancement of Organic Matter Removal in an Integrated Biofilm-Membrane Bioreactor Treating High-Salinity Wastewater. Archaea.

[231] Pathak N., Tran V., Phuntsho S., Shon H.Y. (2020). Chapter 10—Membrane bioreactors for the removal of micro-pollutants. Current Developments in Biotechnology and Bioengineering.

[232] Bilad M.R.P., Westbroek I.V. (2011). Assessment and optimization of electrospun nanofiber-membranes in a membrane bioreactor (MBR). J. Membr. Sci..

[233] Ren L.-F., Ngo H.H., Bu C., Ge C., Ni S.-Q., Shao J., He Y. (2020). Novel external extractive membrane bioreactor (EMBR) using electrospun polydimethylsiloxane/polymethyl methacrylate membrane for phenol-laden saline wastewater. Chem. Eng. J..

[234] Livingston A.G. (1994). Extractive membrane bioreactors: A new process technology for detoxifying chemical industry wastewaters. J. Chem. Technol. Biotechnol..

[235] Wenten I.G., Friatnasary D.L., Khoiruddin K., Setiadi T., Boopathy R. (2019). Extractive membrane bioreactor (EMBR): Recent advances and applications. Bioresour. Technol..

[236] Moradi G., Zinadini S., Rajabi L., Dadari S. (2018). Fabrication of high flux and antifouling mixed matrix fumarate-alumoxane/PAN membranes via electrospinning for application in membrane bioreactors. Appl. Surf. Sci..

[237] Arslan S., Eyvaz M., Güçlü S., Yüksekdağ A., Koyuncu I., Yuksel E. (2020). Investigation of water and salt flux performances of polyamide coated tubular electrospun nanofiber membrane under pressure. J. Environ. Sci. Health.

[238] Fahimirad S., Fahimirad Z., Sillanpää M. (2021). Efficient removal of water bacteria and viruses using electrospun nanofibers. Sci. Total Environ..

[239] Botes M., Cloete T.E. (2010). The potential of nanofibers and nanobiocides in water purification. Crit. Rev. Microbiol..

[240] Rodríguez-Tobías H., Morales G., Grande D. (2019). Comprehensive review on electrospinning techniques as versatile approaches toward antimicrobial biopolymeric composite fibers. Mater. Sci. Eng. C.

[241] Vineis C., Varesano A. (2018). Natural polymer-based electrospun fibers for antibacterial uses. Electrofluidodyn. Technol. Biomater. Med. Devices.

[242] Schabikowski M., Cichoń A., Németh Z., Kubiak W., Kata D., Graule T. (2019). Electrospun iron and copper oxide fibers for virus retention applications. Text. Res. J..

[243] Ren H., Du Y., Su Y., Guo Y., Zhu Z., Dong A. (2018). A Review on Recent Achievements and Current Challenges in Antibacterial Electrospun N-halamines. Colloid Interface Sci. Commun..

[244] Kwon H.-J., Cha J.-R., Lee D.R., Chin B.D., Kim O.Y., Hwang S.-H. (2020). Preparation and Characterization of Antimicrobial Bilayer Electrospun Nanofiber Membrane for Oily Wastewater Treatment. J. Korean Phys. Soc..

[245] Taheran M., Kumar P., Naghdi M., Brar S.K., Knystautas E.J., Verma M., Surampalli R.Y. (2019). Development of an advanced multifunctional portable water purifier. Nanotechnol. Environ. Eng..

[246] Lan S., Lu Y., Zhang J., Guo Y., Li C., Zhao S., Sheng X., Dong A. (2019). Electrospun Sesbania Gum-Based Polymeric N-Halamines for Antibacterial Applications. Polymers.

[247] Abideen Z.U., Kim J.H., Lee J.-H., Kim J.-Y., Mirzaei A., WooKimab H., Kim S.S. (2017). Electrospun Metal Oxide Composite Nanofibers Gas Sensors: A Review. J. Korean Ceram. Soc..

[248] Nazemi H., Joseph A., Park J., Emadi A. (2019). Advanced Micro- and Nano-Gas Sensor Technology: A Review. Sensors.

[249] Manea L.R., Bertea A.-P., Fedorenko Y. (2019). Sensors from Electrospun Nanostructures. Nanostructures in Energy Generation, Transmission and Storage.

[250] Ding B., Wang M., Yu J., Sun G. (2009). Gas Sensors Based on Electrospun Nanofibers. Sensors.

[251] Roto R., Rianjanu A., Rahmawati A., Fatyadi I.A., Yulianto N., Majid N., Syamsu I., Wasisto H.S., Triyana K. (2020). Quartz Crystal Microbalances Functionalized with Citric Acid-Doped Polyvinyl Acetate Nanofibers for Ammonia Sensing. ACS Appl. Nano Mater..

[252] Al-Hazeem N.Z.A., Ahmed N.M., MatJafri M.Z., Bououdina M. (2020). Hydrogen gas sensor based on nanofibers TiO_2_-PVP thin film at room temperature prepared by electrospinning. Microsyst. Technol..

[253] Schoolaert E., Steyaert I., Vancoillie G., Geltmeyer J., Lava K., Hoogenboom R., de Clerck K. (2016). Blend electrospinning of dye-functionalized chitosan and poly (ε-caprolactone): Towards biocompatible pH-sensors. J. Mater. Chem. B.

[254] Teli M., Nadathur G.T. (2020). Reversible colourimetric sensing of volatile phase by dye doped electrospun silica based nanofibers. J. Environ. Chem. Eng..

[255] van den Broek S., Abegg S.E., Pratsinis A.T. (2019). Güntner. Highly selective detection of methanol over ethanol by a handheld gas sensor. Nat. Commun..

[256] Liu W., Xu L., Sheng K., Zhou X., Dong B., Lu G., Song H. (2018). A highly sensitive and moisture-resistant gas sensor for diabetes diagnosis with Pt@In_2_O_3_ nanowires and a molecular sieve for protection. NPG Asia Mater..

[257] Horne J., McLoughlin L., Bridgers B., Wujcik E.K. (2020). Recent developments in nanofiber-based sensors for disease detection, immunosensing, and monitoring. Sens. Actuators Rep..

[258] Al-Dhahebi A.M., Gopinath S.C.B., Saheed M.S.M. (2020). Graphene impregnated electrospun nanofiber sensing materials: A comprehensive overview on bridging laboratory set-up to industry. Nano Converg..

[259] Krishnan S.K., Singh E., Singh P., Meyyappan M., Nalwa H.S. (2019). A review on graphene-based nanocomposites for electrochemical and fluorescent biosensors. RSC Adv..

[260] Cabral T.S., Sgobbi L.F., Delezuk J., Pessoa R.S., Lobo A.O., Rodrigues B.V. (2019). Glucose sensing via a green and low-cost platform from electrospun poly (vinyl alcohol)/graphene quantum dots fibers. Mater. Today: Proc..

[261] Guan H., Zhang J., Liu Y., Zhao Y., Zhang B. (2019). Rapid quantitative determination of hydrogen peroxide using an electrochemical sensor based on PtNi alloy/CeO_2_ plates embedded in N-doped carbon nanofibers. Electrochim. Acta.

[262] Zhu M., Xiong R., Huang C. (2019). Bio-based and photocrosslinked electrospun antibacterial nanofibrous membranes for air filtration. Carbohydr. Polym..

[263] Lv D., Wang R., Tang G., Mou Z., Lei J., Han J., De Smedt S.C., Xiong R., Huang C. (2019). Ecofriendly Electrospun Membranes Loaded with Visible-Light-Responding Nanoparticles for Multifunctional Usages: Highly Efficient Air Filtration, Dye Scavenging, and Bactericidal Activity. ACS Appl. Mater. Interfaces.

[264] Tebyetekerwa M., Xu Z., Yang S., Ramakrishna S. (2020). Electrospun Nanofibers-Based Face Masks. Adv. Fiber Mater..

[265] He H., Gao M., Illés B., Molnar K. (2020). 3D Printed and Electrospun, Transparent, Hierarchical Polylactic Acid Mask Nanoporous Filter. Int. J. Bioprint..

[266] Das O., Neisiany R.E., Capezza A.J., Hedenqvist M.S., Försth M., Xu Q., Jiang L., Ji D., Ramakrishna S. (2020). The need for fully bio-based facemasks to counter coronavirus outbreaks: A perspective. Sci. Total Environ..

[267] Wang X., Ding B., Sun G., Wang M., Yu J. (2013). Electro-spinning/netting: A strategy for the fabrication of three-dimensional polymer nano-fiber/nets. Prog. Mater. Sci..

[268] Hu J., Wang X., Ding B., Lin J., Yu J., Sun G. (2011). One-step Electro-spinning/netting Technique for Controllably Preparing Polyurethane Nano-fiber/net. Macromol. Rapid Commun..

[269] Liu H., Zhang S., Liu L., Yu J., Ding B. (2020). High-Performance PM0.3 Air Filters Using Self-Polarized Electret Nanofiber/Nets. Adv. Funct. Mater.

[270] Liu H., Zhang S., Liu L., Yu J., Ding B. (2020). High-performance filters from biomimetic wet-adhesive nanoarchitectured networks. J. Mater. Chem. A.

[271] Maddah B., Yavaripour A., Ramedani S.H., Hosseni H., Hasanzadeh M. (2020). Fabrication of electrospun PU nanofiber composites based on carbon nanotubes decorated with nickel-zinc ferrite particles as an adsorbent for removal of hydrogen sulfide from air. Environ. Sci. Pollut. Res..

[272] Bortolassi A.C.C., Nagarajan S., Lima B.D.A., Guerra V.G., Aguiar M.L., Huon V., Soussan L., Cornu D., Miele P., Bechelany M. (2019). Efficient nanoparticles removal and bactericidal action of electrospun nanofibers membranes for air filtration. Mater. Sci. Eng. C.

[273] Lu P., Murray S., Zhu M. (2019). Chapter 23—Electrospun Nanofibers for Catalysts. Electrospinning: Nanofabrication and Applications.

[274] Ogunlaja A.S., Kleyi P., Walmsley R.S., Tshentu Z.R. (2016). Nanofiber-supported metal-based catalysts. Catalysis.

[275] Niu Q., Guo J., Chen B., Nie J., Guo X., Ma G. (2017). Bimetal-organic frameworks/polymer core-shell nanofibers derived heteroatom-doped carbon materials as electrocatalysts for oxygen reduction reaction. Carbon.

[276] Misson M., Jin B., Dai S., Zhang H. (2020). Interfacial Biocatalytic Performance of Nanofiber-Supported β-Galactosidase for Production of Galacto-Oligosaccharides. Catalysts.

[277] Liu G., Bonakdarpour A., Wang X., Bi X., Wilkinson D.P. (2019). Antimony-Doped Tin Oxide Nanofibers as Catalyst Support Structures for the Methanol Oxidation Reaction in Direct Methanol Fuel Cells. Electrocatalysis.

[278] Zhong S. (2020). Preparation of chitosan/poly(methacrylic acid) supported palladium nanofibers as an efficient and stable catalyst for Heck reaction. J. Chem. Sci..

[279] Kamaci U.D., Peksel A. (2020). Enhanced Catalytic Activity of Immobilized Phytase into Polyvinyl Alcohol-Sodium Alginate Based Electrospun Nanofibers. Catal. Lett..

[280] Li X., Qi T., Wang J., She W., Mao G., Yan P., Li W., Li G. (2019). Enhanced catalytic performance of nitrogen-doped carbon supported FeOx-based catalyst derived from electrospun nanofiber crosslinked N, Fe-containing MOFs for efficient hydrogenation of nitroarenes. Mol. Catal..

[281] Jia Z., Zhang M., Liu B., Wang F., Wei G., Su Z. (2020). Graphene Foams for Electromagnetic Interference Shielding: A Review. ACS Appl. Nano Mater..

[282] Yang X., Fan S., Li Y., Guo Y., Li Y., Ruan K., Zhang S., Zhang J., Kong J., Gu J. (2020). Synchronously improved electromagnetic interference shielding and thermal conductivity for epoxy nanocomposites by constructing 3D copper nanowires/thermally annealed graphene aerogel framework. Compos. Part A Appl. Sci. Manuf..

[283] Wanasinghe D., Aslani F., Ma G., Habibi D. (2020). Review of Polymer Composites with Diverse Nanofillers for Electromagnetic Interference Shielding. Nanomaterials.

[284] Sancak E., Ozen M.S., Erdem R., Yilmaz A.C., Yuksek M., Soin N., Shah T. (2018). PA6/silver blends: Investigation of mechanical and electromagnetic shielding behaviour of electrospun nanofibers. Tekst. Konfeksiyon.

[285] Shukla V. (2019). Review of electromagnetic interference shielding materials fabricated by iron ingredients. Nanoscale Adv..

[286] Ji H., Zhao R., Zhang N., Jin C., Lu X., Wang C. (2018). Lightweight and flexible electrospun polymer nanofiber/metal nanoparticle hybrid membrane for high-performance electromagnetic interference shielding. NPG Asia Mater..

[287] Kim J., Lee S., Kim C., Park Y., Kim M.-H., Seol J.H. (2020). Electromagnetic Interference Shield of Highly Thermal-Conducting, Light-Weight, and Flexible Electrospun Nylon 66 Nanofiber-Silver Multi-Layer Film. Polymers.

[288] Yuan Y., Li J., Li Y., He X., Yuan Y. (2019). Lightweight and flexible hybrid film based on delicate design of electrospun nanofibers for high-performance electromagnetic interference shielding. Nanoscale.

[289] IEA Online Data Services CO2 Emissions from Fuel Combustion. http://data.iea.org/payment/products/115-co2-emissions-from-fuelcombustion.aspx.

[290] Styring P. (2015). Carbon Dioxide Capture Agents and Processes.

[291] Knapik E., Kosowski P., Stopa J. (2018). Cryogenic liquefaction and separation of CO2 using nitrogen removal unit cold energy. Chem. Eng. Res. Des..

[292] Iqbal N., Babar A.A., Zainab G., Ding B. (2019). Chapter 20—Electrospun Nanofibers for Carbon Dioxide Capture. Micro and Nano Technologies, Electrospinning: Nanofabrication and Applications.

[293] Abbasi A., Nasef M.M., Babadi F.E., Faridi-Majidi R., Takeshi M., Abouzari-Lotf E., Choong T., Somwangthanaroj A., Kheawhom S. (2019). Carbon Dioxide Adsorption on Grafted Nanofibrous Adsorbents Functionalized Using Different Amines. Front. Energy Res..

[294] Heo Y.-J., Zhang Y., Rhee K.Y., Park S.J. (2019). Synthesis of PAN/PVDF nanofiber composites-based carbon adsorbents for CO_2_ capture. Compos. Part B Eng..

[295] Othman F.E.C., Yusof N., González-Benito J., Fan X., Ismail A.F. (2020). Electrospun Composites Made of Reduced Graphene Oxide and Polyacrylonitrile-Based Activated Carbon Nanofibers (rGO/ACNF) for Enhanced CO_2_ Adsorption. Polymers.

[296] Choi C., Kadam R.L., Gailwad S., Hwang K.-S., Han S. (2020). Metal organic frameworks immobilized polyacrylonitrile fiber mats with polyethyleneimine impregnation for CO_2_ capture. Microporous Mesoporous Mater..

[297] Tran D.N., Marti A.M., Balkus K.J. (2014). Electrospun Zeolite/Cellulose Acetate Fibers for Ion Exchange of Pb^2+^. Fibers.

[298] Jaime-Ferrer J.S., Mosqueda-Quintero M., Toriello V.A.S., Anderson S.M., González-Vargas O.A., Villafaña-López L. (2020). Heterogeneous PVC cation-exchange membrane synthesis by electrospinning for reverse electrodialysis. Int. J. Chem. React. Eng..

[299] Kallem P., Yanar N., Choi H. (2018). Nanofiber-Based Proton Exchange Membranes: Development of Aligned Electrospun Nanofibers for Polymer Electrolyte Fuel Cell Applications. ACS Sustain. Chem. Eng..

[300] Zhang S., Tanioka A., Matsumoto H. (2018). Nanofibers as novel platform for high-functional ion exchangers. J. Chem. Technol. Biotechnol..

[301] Jalal N.M., Jabur A.R., Hamza M.S., Allami S. (2020). Sulfonated electrospun polystyrene as cation exchange membranes for fuel cells. Energy Rep..

[302] Alnaqbi M.A., Samson J.A., Greish Y.E. (2020). Electrospun Polystyrene/LDH Fibrous Membranes for the Removal of Cd^2+^ Ions. J. Nanomater..

[303] Johns A., Qian J., Carolan M.E., Shaikh N., Peroutka A., Seeger A., Cerrato J.M., Forbes T.Z., Cwiertny D.M. (2020). Functionalized electrospun polymer nanofibers for treatment of water contaminated with uranium. Environ. Sci. Water Res. Technol..

